# Network analysis reveals a stress-affected common gene module among seven stress-related diseases/systems which provides potential targets for mechanism research

**DOI:** 10.1038/srep12939

**Published:** 2015-08-06

**Authors:** Liyuan Guo, Yang Du, Jing Wang

**Affiliations:** 1 Key Laboratory of Mental Health, Institute of Psychology, Chinese Academy of Sciences, Beijing, China; 2 University of Chinese Academy of Sciences, Beijing, China

## Abstract

Chronic stress (CS) was reported to associate with many complex diseases and stress-related diseases show strong comorbidity; however, molecular analyses have not been performed to date to evaluate common stress-induced biological processes across these diseases. We utilized networks constructed by genes from seven genetic databases of stress-related diseases or systems to explore the common mechanisms. Genes were connected based on the interaction information of proteins they encode. A common sub-network constructed by 561 overlapping genes and 8863 overlapping edges among seven networks was identified and it provides a common gene module among seven stress-related diseases/systems. This module is significantly overlapped with network that constructed by genes from the CS gene database. 36 genes with high connectivity (hub genes) were identified from seven networks as potential key genes in those diseases/systems, 33 of hub genes were included in the common module. Genes in the common module were enriched in 190 interactive gene ontology (GO) functional clusters which provide potential disease mechanism. In conclusion, by analyzing gene networks we revealed a stress-affected common gene module among seven stress-related diseases/systems which provides insight into the process of stress induction of disease and suggests potential gene and pathway candidates for further research.

Chronic stress (CS) influences multiple systems and affects the generation and development of numerous complex disorders^1^,^2^, such as infectious and autoimmune disorders^3^,^4^,^5^, cardiovascular events^6^,^7^, cancers^8^,^9^, mental disorders^10^,^11^,^12^, and obesity^13^. Results from epidemiological literature show strongly that there is comorbidity among stress-related diseases^14^,^15^, and studies of molecular mechanisms also imply a tight relevance across these diseases^16^. Additionally, recent clinical tests suggest that psychological interventions can affect patients with other stress-related diseases^17^,^18^. Although increasing evidence has hinted at a strong association among different stress-related diseases, it remains unclear whether there is a common stress-induced biological process across these diseases.

In recent years, genetic and expressional studies have identified a significant number of disease-related genes, and the information has been organized into specific data resources^19^,^20^,^21^. Moreover, many gene-based bioinformatic approaches, such as gene network analysis that based on the interaction of proteins encoded by genes, so called protein-protein interaction network analysis^22^ and gene co-expression module analysis^23^, have been employed to explore the biological processes that underlie stress-related diseases. Several key genes, biological pathways, and functional modules have been identified in bioinformatics studies.

In this study, we used genes from seven stress-related disease/system databases and one CS database to construct networks based on the interaction information of proteins they encode. These networks were analyzed as follows: 1) identify nodes with high connectivity (hub genes) of these diseases/systems to obtain key genes in the interactive system; 2) reveal a common gene module among different stress-related diseases/systems to provide molecules potentially related to disease comorbidity; and 3) determine the relationship between CS and disease/system common module. Based on the results of the network analysis, a pathway enrichment analysis was performed to determine potential biological mechanisms through which CS induces disease.

## Materials and Methods

### Gene sets of stress-related disease/system

Genes from genetic and expressional databases of neurodegeneration disease, mental disorders and other stress related diseases or systems were obtained and utilized for network analyses. Genes from Alzgene^24^, BDgene^21^, MK4MDD^19^, CADgene^20^, and NCG4.0^25^ were selected to form gene sets of five stress-related diseases: Alzheimer’s disease(Alz), bipolar disorder(BD), major depressive disorder(MDD), coronary artery disease(CAD)and cancer. The BDgene and MK4MDD databases include genetic factors that are linked to BD and MDD and have positive and negative results; only genes with at least one positive result were selected for the corresponding gene sets. Genes from the Obesity Gene Atlas^26^ and Immunome^27^ were also selected for inclusion in gene sets for fatty metabolism and immune responses. Human CS genes in the database CS-DEGs^28^ were selected to form a gene set that is affected by CS environments. Overlaps were compared among the stress-related diseases and system gene sets.

### Protein-protein interaction networks

**Figure 1:**
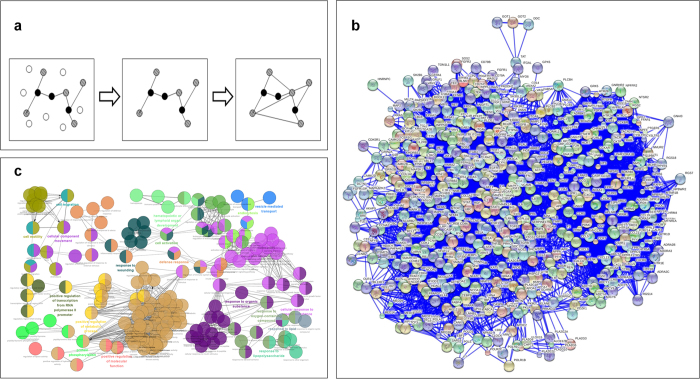
**a**) Flowchart of gene network construction. Black nodes: seed nodes; striated nodes: first neighbors of seed nodes; and white nodes: other nodes in the STRING interactome. (**b**) The common sub-network among seven stress-related diseases/systems. (**c**) Largest functional GO group enriched by genes of stress-related disease/system common module.

The database STRING 9.1^29^ provides a comprehensive protein interactome that includes known and predicted protein-protein interactions scored according to their confidence. Information in this database was utilized to construct the disease/system and CS gene networks. Genes in the stress-related disease/system or CS datasets were considered seed nodes and used to obtain protein-protein interactions with the highest confidence (score >0.9). As shown in Fig. 1a, an extended network that included seed nodes, first neighbor nodes, and the highest confident interactions between these nodes was constructed for each gene set. All of the networks were visualized and analyzed with the visualization software Cytoscape 3.0.2^30^. The node properties, such as the betweenness centrality (BC) and degree, were calculated using the plug-in “Network Analyzer”^31^ in the Cytoscape software. Hub genes were identified according to the following thresholds: BC > 0.05 and degree >50^22^. The statistical significant difference between properties of nodes in disease/system networks and the entire interactome was examined by T-test.

### Common gene module of stress-related diseases/systems

To examine whether a common gene module exists among different stress-related diseases/systems, nodes and edges were compared among the seven stress-related disease and system networks and a common sub-network was constructed using the overlapping nodes and edges. These interactive nodes in the sub-network constructed the common gene module. Three properties of the module were analyzed: 1) network topological parameters; 2) overlapping genes shared between the module and hub genes; and 3) overlapping genes shared between the module and the CS gene set and network. The statistical significance of the overlap between common module and the CS gene set and network was determined by the Fisher’s exact test.

### Gene ontology (GO) pathway cluster enrichment analysis

To identify common biological processes underlying stress-related diseases, a GO pathway cluster enrichment analysis was performed on nodes in the disease/system common module using the online analysis tool DAVID^32^. As recommended in DAVID, the cutoff for pathway cluster enrichment was set at a score >1.3. The representative biological terms associated with significant clusters were manually selected. Because these clusters reflect interactive functional systems, the GO term network of genes in common module was also deciphered using the Cytoscape plug-in ClueGO^33^ to provide a system-wide view.

## Results

### Summary of genes and networks

The seven stress-related disease/system gene sets included 4637 genes (summarized in Supplementary Table S1). The seven gene sets overlapped; however, there were no genes that occurred in all seven sets. The genes that occupied by more than four gene sets are shown in Supplementary Table S2. The CS gene set included 2606 genes (see in Supplementary Table S1). A total of 3941 disease/system or CS genes were found in STRING v9.1, and 8429 nodes were included in the disease/system or CS networks (see in Supplementary Table S1).

### Hub genes

Node properties in each network were analyzed. As shown in Supplementary Table S3, the average degrees of the disease/system nodes were all significantly higher than the average degree of the entire STRING network. With a threshold degree >50 and BC > 0.05, as shown in Table 1, 36 genes were identified as hub genes for seven diseases/systems and the genes *ESR1, TP53, FOS, AKT1*, and *FRN* were hub genes for more than one disease/system.

### Common gene module among stress-related diseases/systems

To explore the common biological modules underlying stress-related diseases/systems, the nodes and edges of the seven disease/system networks were compared. A common sub-network including 561 genes and 8863 edges was observed in the network of all seven diseases/systems (Fig. 1b). The 561 interactive common genes (as shown in Table 2) constructed the common gene module among stress-related diseases/systems, they include 180 members of the CS gene set, and all genes in this module can be found in the CS network. Nodes of the CS network significantly overlapped with the common gene module (Fisher’s Exact Test, p < 2.2E-16). The average degrees of genes in common module were significantly higher than other genes in disease/system networks (as shown in Supplementary Table S4). 33 hub genes were included in the common module; hub genes in Table 1 that were not included in the common module were *ACTN2, CDC42*, and *OR6A2*.

### Functional pathways enriched by genes in common module

Using the recommended threshold enrichment score (>1.3), 190 GO functional pathway clusters were enriched and categorized into three types: *Cellular Component* (see in Supplementary Table S5), *Biological Process* (see in Supplementary Table S6) and *Molecular Function* (see in Supplementary Table S7). Table 3 shows the top 10 enriched clusters and the detailed information of all enriched clusters was shown in Supplementary Table S8. Fifty-four interactive pathway groups were identified with network connectivity (Kappa score) 0.5. The largest group is shown in Fig. 1c, and all groups are shown in Supplementary Figure S1.

## Discussion

In this study we constructed seven stress-related disease/system gene networks based on the interaction information of gene-encoded proteins. The average degrees of the disease/system genes are significantly higher than the average degrees of the entire human interactome, suggesting that disease/system genes and their first neighbors are more highly connected in the human interactome than random genes, so they may play roles in a tighter and more complex manner. The result also supports the hypothesis that disease genes tend to have higher degrees^34^,^35^. A total of 36 disease/system genes were identified as hub genes that occupy central positions in disease/system networks and may possess important biological functions.

Although common genes were not identified among the stress-related diseases/systems compared in this study, a common sub-network was identified among the seven disease/systems. Genes in this sub-network were most enriched in GO pathways that related to chemical homeostasis (as shown in Table 3). This result may imply that there is a common interactive gene module that maintains homeostasis which is related to all stress-related diseases/systems, so this common module provides potential molecular fundaments of the comorbidity. Because most hub genes are included in the common module, the dysfunction of this module may play an important role in disease generation and development. The imbalance of homeostasis induced by aberrant expression of genes in the common module may trigger a pre-disease state^36^ with the potential to develop into different pathological processes because of additional disease/system genes that were not found in the common module. In each disease/system network, the average degrees of nodes in common module were significantly higher than other nodes. Considering the reports that that disease genes tend to have higher degrees^34^,^35^, genes in common module may be more strongly associated to pathological processes than other genes in disease/system networks.

The CS gene set includes human genes whose rodent homologs were differentially expressed in CS rodent models. The significant overlap between the common gene module of seven human diseases/systems and CS network suggests that the stress environment may induce disease by influencing a common homeostasis system. Consequently, a pre-disease state progresses to different disease states as genetic factors and/or other environmental factors are stimulated. This potential mechanism may explain the concomitant strong association between stress and disease and high heterogeneity of pathological processes associated with stress-related diseases. Genes in the common module (as shown in Table 2) could be useful candidates for subsequent experimental study.

The GO pathway clusters enriched by genes in the common module indicate the biological systems that are influenced by stress environments and abnormal in diseases, so they may imply the biological mechanisms by which stress environments induce disease. Beyond that, these pathway clusters also provide specific potential targets for relevant research. As shown in Supplementary Table S5, most of the common nodes are located in the extracellular space and plasma membrane-related cellular components, which suggests candidate targets for disease intervention. The biological processes associated with common module (see in Supplementary Table S6) provide a series of candidates for mechanism research, such as processes related to response, metabolism, cell differentiation and migration, transport, and signaling transduction. The enriched pathway clusters of molecular function, such as peptide receptor activity, and phospholipase activity, suggest potential drug targets (Supplementary Table S7). These functional pathways are interactive systems and could be enriched in several groups (as shown in Supplementary Figure S1). Figure 1c shows the largest enriched interactive group that constructed by pathways of response, regulation, cell migration, transport, and signaling transduction. Besides of the system views, certain enriched biological process clusters provide detailed biological hypotheses for specific diseases. For example, the dysfunction of 37 genes in the function cluster “response to bacterium”(Supplementary Table S6 and Table S8) may directly mediate the process by which stress stimulates infectious disease. This function cluster also provides a potential explanation for the comorbidity among infectious diseases and other stress-related diseases.

In conclusion, we utilized stress-related disease/system genes to construct interactive networks. By analyzing these networks, we identified hub genes which may play roles in the pathological processes of stress-related diseases. We also identified a common sub-network among diseases/systems and the sub-network is significantly overlapped with the CS network. The common sub-network implies that different stress-related diseases/systems share a common gene module that may be influenced by stress environments. By analyzing this common gene module, the potential mechanism underlying the process by which stress induces diseases could be partially revealed.

In spite of above results, this study also has some limitations. First, we constructed network based on existing annotations database which are limited by our current knowledge of biology. Second, limited by the lack of data resource, only seven stress-related diseases or systems were selected to analyze. Third, the CS genes were obtained via homologous analysis on differentially expressed genes from CS rodent models, so result based on these genes need to be further validated in human study.

## Additional Information

**How to cite this article**: Guo, L. *et al.* Network analysis reveals a stress-affected common gene module among seven stress-related diseases/systems which provides potential targets for mechanism research. *Sci. Rep.* **5**, 12939; doi: 10.1038/srep12939 (2015).

## Supplementary Material

Supplementary Information

## Figures and Tables

**Table 1:** Hub genes in seven stress-related disease/system networks.

Network name	Hub genes
Alz	*ESR1, TP53, APP, FOS*
BD	*ESR1, IL6, CREBBP, AKT1, FYN*
MDD	*ESR1, MAPK1, CCND1, EP300, PIK3R1, FOS, CDC42, JUN*
CAD	*ESR1, TP53, EGFR, SRC, PTGS2*
Cancer	*TP53, AKT1, OR6A2, CTNNB1, MYC*
Immune	*MAPK14, STAT3, POMC, LCK, FYN*
Obesity	*APOA1, PPATA, PPARG, ALB, S1PR1, LPL, F2R, GRB2, PKLR, DRD2, ACTN2, RXRA*

**Table 2:** Genes in diseases/systems common module.

Type	Gene symbol
CS gene	CREBBP, TAC1, PAFAH1B1, NOS2, FYN, IGF1, VIM, KITLG, CAMK2B, ERBB3, TNFRSF1A, PRKCZ, DRD4, TFRC, SREBF1, PTGS2, ACTB, IL4R, FGFR1, MAP2, ADCY2, BAX, ITGA6, TF, ESR1, BLNK, MAPK1, HIF1A, HRH3, PDYN, POLA1, HTR2A, KLF5, TGFB1, MAPK8, KPNB1, CCL2, COL1A1, YWHAE, NFKB1, IL2, CCKBR, CDKN1B, OPRM1, VEGFA, IL4, CBLB, POMC, TACR1, FN1, ID2, RGS4, APOA1, CREB1, TTR, CTGF, GOT1, GNA13, TEK, NFYB, PTK2B, PIK3CA, NPY, SDC4, PLCG1, IL1B, C3, PLA2G5, KDR, MCHR1, LDLR, APOE, PPARG, PTPRA, VAV3, GRP, ANXA1, CDK4, TEC, EDNRB, IL6, TLR4, CCL21, ITGAV, CXCL9, RB1, IGF1R, TP53, APP, AKT1, HSPG2, PKLR, GPR17, AGTR1, SNCA, CAMK2D, IGFBP3, IL2RG, GNRH1, GSN, PLCB3, GRIN2B, ITGB1, ADRA2A, PRKCA, EPHB1, ADCY8, SST, KIT, FCER1G, IL6R, CCL5, HCRT, ALOX15, ADCY9, VCAM1, ADORA1, PRKCD, NPY1R, CSF2, COL1A2, ADCY1, CDK5, STAT3, GDNF, AR, RGS14, EFNA1, FABP4, FOS, CD14, NPAS2, KISS1, S1PR1, HDAC3, BCL2, PRL, CEBPB, RELA, ADCY5, LPL, A2M, JUN, HTR1A, OXTR, ADIPOQ, PLCB1, GRIN2A, ADRA2C, NPY2R, SOCS1, MAPK11, SP1, PPARA, CCK, PLA2G2A, AVPR1B, LPAR1, TAT, LTB4R, ADRA2B, TNF, LTF, NCOA1, DNM1, SH3GL2, LRP1, AKAP9, ACTN4, NTS, RAC1, CAMK2G, PAK1, DRD2, TRH, PRKCE, GAL, MAPK3, HTR1B, PENK
First neighbor of CS gene	LCP2, BCAR1, ITGB4, EFNB1, VCL, SIRT1, PLA2G3, IL2RB, SOS2, BDKRB1, TIMP1, POLR2C, RETN, CD79A, EPOR, PIK3R2, HGF, WASL, SERPINE1, RPS6KB1, GNRHR, CCND1, APOC3, PXN, VDR, GAPDH, CHPT1, MAPK14, IL5, APOB, OPRD1, RGS2, IAPP, NR4A1, GOT2, VASP, S1PR4, FFAR1, CCR7, RRAS, CANX, TRAF2, GALR3, MYOD1, MED17, APOC2, SDC1, SSTR4, NMUR2, CHRM3, KRAS, IL5RA, HRH4, MTNR1B, APLNR, PDGFRA, HTR2B, TGS1, MMP7, MMP13, THBS1, PLCB2, LMNB1, SCARB1, PDGFRB, ITGA2B, GNA15, TCF3, PLD2, EP300, NOX4, POLR1B, ELANE, SELP, LRP2, CBL, NMU, SHC2, ITGA9, PPARGC1A, F13A1, SCARB2, KNG1, AMBP, CASP6, EGF, OPRK1, NPFF, SSTR1, TPM1, IQGAP1, NCOR1, ARRB2, ERBB2, REN, ACKR3, RAB5A, PIK3R1, RASA1, EGFR, HTR2C, CD44, VEGFC, MCHR2, CYSLTR2, PTH, MED21, CXCL13, ITGAM, HTR5A, CTBP1, CXCR5, CCR5, PTGER1, SSTR5, CCKAR, MITF, CXCR1, ALB, CXCL5, PPBP, CASR, F2RL2, F2RL1, IL3, ANGPT1, GPER1, FIGF, INPPL1, AVPR1A, FRS2, MMP3, STAT6, CRK, NFATC3, PELP1, CTTN, PNOC, PTAFR, VAV1, MAP2K1, BCL2L1, FPR1, MTNR1A, ITGB2, BCR, LRP8, NTSR2, INSR, RAC3, CXCR6, SH2B2, P2RY1, IRS1, CXCL10, NMUR1, IL8, ADRA1B, SDC2, BDKRB2, HTR1E, NPFFR2, CD36, P2RY14, F2, CLOCK, CCL19, PLG, OLR1, YWHAZ, P2RY6, TRHR, HRAS, ADAM17, P2RY2, PSMC5, CASP3, ADCY6, SEC13, ADCY4, ADRBK1, PLA2G1B, LEP, GNAI2, HTR1D, KHDRBS1, MED29, EDNRA, ALOX12B, MET, CXCR2, HNRNPC, GPR4, CHRM2, PKM, F2R, MAPK9, CDC25C, POLE, MMP1, EBF1, HTR1F, MED14, YES1, CARM1, CCR8, NFATC1, S1PR5, TSPO, GALR2, SSTR3, NPBWR1, PDGFB, GAST, CALCA, CD86, SMAD3, PLA2G6, RGS19, PRKD1, PLCB4, GNG2, ADORA2A, OPRL1, SH2B1, MED19, LCK, FGF1, IL6ST, GRB2, CAV1, CCR6, FPR2, KAT5, GIPC1, B2M, PTPN11, PTK2, SMAD4, FPR3, PDZK1, MED22, JAK1, BRE, GPR18, ESR2, CTNNB1, UBC, E2F1, ANXA2, CRKL, C5AR1, RET, NCOR2, INS-IGF2, CCR3, SSTR2, APOA4, DDC, SMARCA4, S1PR3, SRC, EPHA2, ARNT, MAX, NCOA6, CD2AP, INPP5D, DNM2, COL18A1, NPSR1, PYY, MYLK, STAT1, MTOR, DCTN1, RGS7, FGFR1OP, MAPKAPK2, RGS1, RGS18, PLA2G4A, SERPINC1, FASLG, MED23, LMNA, RXFP4, ADAM12, NRAS, ADORA3, NPBWR2, FLNA, GNAI3, MYO6, GTF2B, LPAR3, PTGFR, PTPN1, VAV2, AGTR2, NCOA3, RAPGEF1, MMP9, ABL1, POLR1C, PLAU, CCND3, CYSLTR1, TACR2, HDAC1, MED18, FGR, NPY4R, KLF4, ABCA1, PLA2G2D, TBXA2R, F7, SYK, HCK, IRS2, NPPA, TLE1, WAS, GNB2L1, NMS, PRKACG, MYC, NPHS1, CD83, EDN1, IL2RA, GRPR, MIS12, JAK2, DRD3, ARNTL, HCAR2, GPR55, SUMO1, CD79B, GRK5, ARRB1, ACTN1, NFATC2, ITGA4, NPS, GCGR, MLLT4, LPAR2, SOS1, ARHGAP35, CEBPD, GCG, PTPN6, JAK3, GFRA1, PLA2G10, NCOA2, SHC1, TBL1XR1, GPX1, HDAC9, TOM1L1, CHRM4, FGFR2, TGFBI, RXRA, LYN, DOCK1, ITGB3, GAB2, GPX5, POLD1, PRIC285, CDK1, GNRHR2, S1PR2, LPAR4, CETP1, SAR1B

**Table 3:** Top 10 pathway clusters enriched by genes of disease/system common module.

Annotation cluster	Representative annotation terms	Enrichment score
1	chemical homeostasis	41.148
2	regulation of cell migration	37.584
3	regulation of phosphate metabolic process(MAPK)	37.228
4	response to wounding	33.609
5	response to endogenous(hormone) stimulus	31.744
6	cell migration	30.073
7	Chemotaxis	29.056
8	regulation of lipase and hydrolase activity	28.855
9	second-messenger-mediated signaling	27.447
10	plasma membrane	25.366
