# Supplementary material for: Network analysis reveals a stress-affected common gene module among seven stress-related diseases/systems which provides potential targets for mechanism research
Source: Sci Rep. 2015 Aug 6;5:12939. doi: 10.1038/srep12939 (PMC4526881; doi:10.1038/srep12939)
Supplement: Supplementary Information [file srep12939-s1.pdf]

Network analysis reveals a stress-affected common gene module among seven stress-related diseases/systems which provide potential targets for mechanism research

Liyuan Guo[1 *], Yang Du[1, 2] and Jing Wang[1*]

[1]Key Laboratory of Mental Health, Institute of Psychology, Chinese Academy of Sciences, Beijing, China

[2]University of Chinese Academy of Sciences, Beijing, China

*Correspondence:

1. Jing Wang, Ph.D., Key Laboratory of Mental Health, Institute of Psychology, Chinese Academy of Sciences, 16 Lincui Road, Chaoyang District, Beijing, 100101, China. Email: wangjing@psych.ac.cn Phone: 86-10-64855841 Fax: 86-10-64855841

2. Guo Liyuan, Ph.D., Key Laboratory of Mental Health, Institute of Psychology, Chinese Academy of Sciences, 16 Lincui Road, Chaoyang District, Beijing, 100101, China. Email: guoly@psych.ac.cn Phone: 86-10-64855841 Fax: 86-10-64855841

Supplementary materials list

Page 3: **Table S1**. Data statistics of the genes in the gene sets and networks.

Page 4: **Table S2.** Genes occupied by more than 4 stress-related diseases/systems.

Page 5: **Table S3**. Average degrees of seven disease/system networks and entire STRING network.

Page 6: **Table S4.** Average degrees of the genes in common nodes and all nodes in disease/system networks.

Page 7: **Table S5.** Common module enriched GO pathway clusters and detail genes in each cluster – category of *Cellular Component*.

Page 8: **Table S6.** Common module enriched GO pathway clusters and detail genes in each cluster – category of *Biological Process*.

Page 15: **Table S7.** Common module enriched GO pathway clusters and detail genes in each cluster – category of *Molecular Function*.

Page 16: **Table S8.** GO pathway clusters enriched by genes of stress-related disease/system common module.

Page 81: **Figure S1.** Functional GO groups enriched by genes of stress-related disease/system common module.

Table S1. Data statistics of the genes in the gene sets and networks.

**Set name**	**Gene number**	**Seed gene number (In STRING v 9.1)**	**Network node number**
Alzheimer's disease (Alz)	679	562	4206
Bipolar disorder (BD)	845	557	3940
Major depressive disorder(MDD)	756	551	4425
Coronary artery disease (CAD)	604	526	3805
Cancer	2000	1214	6021
Immune system (Immune)	874	652	3024
Obesity	166	131	1306
Chronic stress (CS)	2606	1607	6640

Table S2. Genes occupied by more than 4 stress-related diseases/systems.

Gene symbol	Disease/system count
IL10	6
CD36	6
ACE	6
TNF	6
IL1B	6
GSK3B	5
ABCB1	5
IL6	5
IFNG	5
TLR4	5
IL1A	5
CCL2	5

Table S3. Average degrees of seven disease/system networks and entire STRING network.

**Network**	**Seed node average degree**	***P* value (T-test)(compared with STRING average degree)**
Alz	25.135	8.41E-11
BD	22.305	1.19E-06
MDD	22.67	8.88E-08
CAD	27.105	5.71E-08
Cancer	21.793	7.33E-10
Immune	20.633	1.46E-05
Obesity	21.267	0.014
STRING	15.949	--

Table S4. Average degrees of the genes in common nodes and all nodes in disease/system networks.

**Network name**	**Average degree of common nodes**	**Average degree of all nodes**	***P* value (T-test)**
Alz	53.984	22.379	1.07967E-55
BD	52.389	24.421	2.63371E-49
MDD	54.069	24.148	1.40427E-51
CAD	53.783	22.778	6.13458E-54
Cancer	57.005	21.763	1.41473E-58
Immune	48.085	22.217	2.99846E-46
Obesity	37.041	22.504	3.59185E-27

Table S5. Common module enriched GO pathway clusters and detail genes in each cluster – category of *Cellular Component*.

**Enriched functional cluster**	**Enrichment score**	**Gene**
Cell projection	11.41792	GNA13,MCHR1,ADCY4,ADCY2,TACR1,BCAR1,SNCA,CD2AP,ADORA1,TGFB1,IQGAP1,CTNNB1,AKT1,APP,CTTN,HTR1A,GOT1,GRIN2B,GSN,APOE,PIK3CA,PLD2,AR,MYO6,ACTN4,RXRA,ESR1,GRIN2A,ACTN1,ESR2,CDK5,VASP,MAPK1,SDC1,SSTR3,HIF1A,CHRM4,CHRM3,CHRM2,RAB5A,ADAM17,WASL,CCK,DRD3,ADORA2A,DRD2,ERBB2,VIM,OXTR,TAC1,ADRBK1,BDKRB1,GIPC1,TPM1,ITGB1,PXN,VCAM1,CALCA,IGF1R,PTK2,RAC3,PTK2B,RAC1,PAFAH1B1,SH2B2,ADRA2C,SCARB1,NMU,RASA1,ACTB,MAP2K1,MET,ANXA1,RGS19,NPY1R,STAT1,LRP1,MAP2,MTOR,LRP2,PDZK1,HTR2A,CCL2,IAPP,SOS1,SST,GNB2L1
Nerve terminal	3.892563	CCK,MAP2K1,ADORA2A,ESR1,ADORA1,CALCA,MAPK1,GOT1,CHRM4,HTR1A,CHRM3,CHRM2,NMU,CALCA,CCK,GOT1,CHRM4,CHRM3,CHRM2,ESR1,ADORA1,NMU,CALCA,CCK,ESR1,ADORA1,NMU
membrane	10.93979	HRAS,PTGS2,ADCY8,ADCY6,SNCA,RPS6KB1,MED22,CTNNB1,APOB,APP,GOT1,GRIN2B,SERPINE1,INSR,FRS2,EFNB1,GRIN2A,CHPT1,IRS1,MAPK1,SSTR2,CD36,SSTR1,ARRB1,F2,FGFR1,CAV1,GNAI3,DRD3,GNAI2,CYSLTR1,ADORA2A,ADRBK1,BDKRB1,GIPC1,ABCA1,POMC,CCL5,TRH,ITGB1,KRAS,RAC1,SCARB1,SCARB2,EGF,MAP2K1,GNRH1,MET,PTPN11,PLA2G4A,CXCL13,MAP2,ADRA1B,FABP4,PYY,MTOR,PTPN1,GRK5,OPRM1,BCAR1,AKT1,EDNRB,FOS,CTTN,PLCB3,IAPP,NMUR1,PLCB1,NPFF,PRKCA,LYN,CCKBR,TP53,AMBP,CHRM4,CHRM2,RAB5A,PRKCZ,YWHAZ,IGF1R,HRH3,BCL2,HRH4,PAFAH1B1,YES1,ACTB,SELP,IL5,OLR1,LMNA,RGS19,PLG,ANXA2,GCG,NRAS,LRP1,NTS,BAX,NPHS1,JAK2,LRP2,OPRD1
Plasma membrane	25.36551	ADCY4,HRAS,ADCY1,ADCY2,ADORA3,PTGS2,IL6ST,ADCY8,EFNA1,ADCY5,SNCA,ADCY6,LPAR4,LPAR3,LPAR2,LPAR1,CD2AP,ADORA1,CTNNB1,B2M,S1PR2,GOT2,AGTR1,S1PR3,AGTR2,HTR1B,APOB,APP,HTR1A,S1PR1,GRIN2B,CD44,APOE,CTGF,LTB4R,S1PR4,S1PR5,SERPINE1,GNG2,NPSR1,HTR1D,HTR1F,SYK,HTR1E,PLD2,C5AR1,EFNB1,GRIN2A,F7,PDYN,SSTR4,CCR8,SSTR5,CCR7,SSTR2,CCR6,SSTR3,CD36,CCR5,SSTR1,CCR3,F2,PDGFRA,PDGFRB,SH3GL2,ITGA2B,GNAI3,GNAI2,ERBB3,ERBB2,TRHR,BDKRB1,ADRBK1,BCL2L1,BDKRB2,EPHB1,PXN,KRAS,RAC1,FCER1G,BLNK,MAP2K1,SMAD3,EPHA2,KDR,RGS1,AVPR1B,AVPR1A,RGS7,GRK5,IL5RA,GNB2L1,PDZK1,PLAU,OPRM1,CCKAR,MCHR1,MCHR2,LDLR,BCAR1,EDNRA,EDNRB,NMUR1,NMUR2,IL4R,HTR5A,RXFP4,LYN,CCKBR,NPBWR1,NPBWR2,IL6R,FLNA,AMBP,RAB5A,ADAM17,ADAM12,PLA2G3,PLA2G5,TF,NPY2R,OPRK1,FFAR1,FPR1,FPR3,FPR2,KIT,GCGR,PTK2B,SOS1,SERPINC1,NPFFR2,THBS1,MLLT4,RASA1,GNRHR2,SELP,IL6,OPRL1,LMNA,NPY1R,PTGFR,STAT3,NRAS,LRP1,ADCY9,TFRC,FYN,P2RY14,NPHS1,GFRA1,LRP8,LRP2,HTR2B,HTR2C,CD14,DNM2,HTR2A,F2RL2,GNA13,CASR,GNA15,F2RL1,RPS6KB1,TLR4,IQGAP1,VCL,APOA1,GALR3,GALR2,RRAS,INSR,FRS2,EGFR,IRS2,MYO6,ACTN1,NTSR2,IRS1,GPR55,ARRB2,ARRB1,COL1A2,COL1A1,FGFR2,FGFR1,CAV1,CYSLTR1,DRD3,ADORA2A,DRD2,CYSLTR2,DRD4,ITGB4,ITGB2,ITGB3,ABCA1,TRH,ITGB1,ITGAM,SRC,PTK2,P2RY6,P2RY2,ITGAV,TEK,P2RY1,ADRA2A,GRPR,ADRA2C,SCARB1,ADRA2B,SCARB2,EGF,FN1,LPL,VAV3,MET,ITGA4,VAV2,VAV1,ITGA9,ITGA6,PLCG1,MTNR1B,TBXA2R,ADRA1B,CD79B,MTOR,CD79A,PTPN1,MTNR1A,TACR2,TACR1,CXCR1,FASLG,CXCR2,GNRHR,SDC4,SDC2,AKT1,CASP3,CXCR5,CXCR6,SHC1,PAK1,PRKCA,PTGER1,GPR18,ESR1,PRKCE,PRKCD,VASP,PRKD1,CD83,SDC1,CD86,CHRM4,CHRM3,CHRM2,LCK,GPR17,PTAFR,PRKCZ,HCRT,TNF,VIM,OXTR,TAC1,KITLG,TPM1,GPR4,VCAM1,IGF1R,APLNR,TNFRSF1A,HRH3,HRH4,CAMK2D,SH2B2,IL2RG,NOX4,IL2RB,IL2RA,OLR1,CBL,PTPRA,ANXA1,RGS19,ANXA2,EPOR,JAK2,CRK,F2R,OPRD1,PLA2G4A,CANX,SUMO1,BCL2,TSPO,RET,CHPT1,SREBF1,SEC13,KPNB1,HCK,BAX
Membrane-bounded vesicle(secretory granule)	12.36946	HCRT,TF,A2M,GNAI3,PDGFB,F13A1,ITGB3,KIT,TRH,POMC,TGFB1,TIMP1,APP,APOA1,ALB,PLA2G1B,IL1B,THBS1,EGF,FIGF,PRL,FN1,SELP,ACTN4,ACTN1,IGF1,HGF,GAL,VEGFC,PLA2G4A,CD36,PPBP,NTS,VEGFA,PLA2G2A,RAB5A,ITGA2B,APOB,GNA13,LDLR,FASLG,CD2AP,CANX,GRIN2B,EGFR,MYO6,WAS,YWHAZ,DRD3,GRB2,CAMK2G,GIPC1,ABCA1,ITGB1,RAC1,CAMK2D,CAMK2B,RGS19,NPY1R,YWHAE,ANXA2,LRP1,TFRC,LRP2,ARRB2,CAV1,APLNR,SREBF1,PTPN1
Basolateral plasma membrane (adherens junction)	5.633854	OPRM1,TF,CAV1,ERBB3,NPY2R,ERBB2,BCAR1,ITGB4,SDC4,ADORA1,ITGB1,PXN,VCL,CTNNB1,PTK2,P2RY6,CD44,PTK2B,P2RY2,P2RY1,PAK1,EGFR,NOX4,MET,ANXA1,ACTN1,VASP,SDC1,ITGA6,ADAM17,ITGA2B,OXTR,MLLT4,HCRT,PRKCZ,RPS6KB1,GRIN2B,GRIN2A,CHRM4,CHRM3,CHRM2,DNM2
Extracellular space	14.23646	A2M,LDLR,IL6ST,INS-IGF2,MMP9,EDN1,MMP7,FASLG,MMP3,TGFB1,CXCL10,APOA4,TTR,APOB,APOA1,GSN,APOE,TGFBI,PLA2G1B,IL1B,ANGPT1,CTGF,EGFR,KNG1,IL6R,MMP13,GRP,RETN,VEGFC,PPBP,F2,VEGFA,COL1A2,PLA2G2A,PLA2G3,CSF2,TF,CCL2,TNF,CCK,CXCL5,ERBB3,C3,CXCL9,APOC2,KITLG,TAC1,KIT,CCL5,VCAM1,CALCA,ALB,CCL21,REN,APOC3,PTH,SERPINC1,THBS1,EGF,FIGF,FN1,COL18A1,IL4,IL3,SELP,LPL,IL6,IL5,GNRH1,IL8,IGF1,CCL19,ADIPOQ,PLG,LEP,NPY,CXCL13,LRP8,IL5RA,LRP2,IGFBP3,SST,IL2,MMP1,GRIN2B,CD44,CTGF,COL1A1,TIMP1,HSPG2,ANXA2,F2RL2,PDGFB,F13A1,GDNF,VCL,B2M,APP,SERPINE1,ACTN4,ACTN1,F7,PDYN,PLA2G2D,FGFR2,ERBB2,GAST,POMC,TRH,HGF,NPS,PENK,PYY,PLAU,KISS1,IAPP,GPX5,IL4R,LTF,PRL,NPFF,ELANE,GAL,FLNA,AMBP,ADAM12,PLA2G5,NPPA,TNFRSF1A,NMS,NMU,PLA2G10,OLR1,GCG,NTS,PNOC,TFRC,EPOR,CD14,F2R
Endocytic vesicle	3.305173	EGFR,TF,MYO6,DRD3,LDLR,CAMK2G,GIPC1,ABCA1,CD2AP,APOB,APOA1,CAMK2D,RAB5A,CAMK2B,LRP2,SELP,GRB2,ITGB3,WAS,CD36,ITGA2B
Postsynaptic density	2.118827	YWHAZ,CHRM4,GRIN2B,LYN,ADORA2A,PTK2B,SOS1,GRIN2A,ADORA1,DNM2,HCRT,ERBB3,ERBB2,GIPC1,ADRBK1,RPS6KB1,ITGB1,CTNNB1,APP,INSR,EFNB1,NPY1R,CDK5,CHRM3,CHRM2,RAB5A,F2R

Table S6. Common module enriched GO pathway clusters and detail genes in each cluster – category of *Biological Process*.

**Enriched functional cluster**	**Enrichment score**	**Gene**
chemical homeostasis	41.14757	GNA13,GNA15,CASR,IL6ST,SNCA,F2RL1,EDN1,LPAR3,LPAR2,LPAR1,ADORA1,TGFB1,APOA4,S1PR3,AGTR1,APP,APOB,S1PR1,APOA1,GOT1,GRIN2B,APOE,S1PR4,SERPINE1,GALR2,IL1B,INSR,IRS2,C5AR1,GRIN2A,NTSR2,IRS1,PPARGC1A,CCR8,SSTR5,CCR7,HIF1A,CCR6,CCR5,CCR3,JUN,VEGFA,F2,CAV1,CCL2,CYSLTR1,DRD3,ADORA2A,DRD2,ERBB2,TRHR,DRD4,APOC2,BDKRB1,BCL2L1,BDKRB2,ABCA1,CCL5,VDR,P2RY2,APOC3,SCARB1,LPL,PTPN11,KDR,PLA2G4A,CXCL13,AVPR1B,ADRA1B,AVPR1A,FABP4,CCKAR,MCHR1,LDLR,INS-IGF2,TACR1,PPARG,EDNRA,EDNRB,NMUR2,LTF,MYC,KNG1,PRKCA,CCKBR,ELANE,TP53,CDK5,LCK,TF,PRKCZ,HCRT,CCK,FFAR1,TAC1,OXTR,CALCA,HRH3,PTK2B,BCL2,PTH,CAMK2D,OPRL1,IGF1,CCL19,NPY1R,ADIPOQ,STAT3,TFRC,BAX,EPOR,JAK2,F2R,IL2,CTNNB1,EP300,PDGFRB,TIMP1,RAC1,CREBBP,AKT1,GPX1,CASP3,LYN,RB1,SH2B2,IL6,IL2RA,PLG,ID2,SP1,HTR2A,
regulation of cell migration	37.58377	GNA13,PDGFB,IL6ST,INS-IGF2,TACR1,MMP9,BCAR1,F2RL1,EDN1,SNCA,RPS6KB1,ADORA1,TGFB1,CXCL10,VCL,AKT1,AGTR2,S1PR1,APOE,RRAS,INSR,PRKCA,EGFR,IRS2,ELANE,IL6R,F7,IRS1,CDK5,MAPK1,VEGFC,HIF1A,VEGFA,PDGFRA,PDGFRB,ADAM17,DRD3,ADORA2A,DRD2,TAC1,BDKRB1,ITGB3,KIT,TPM1,IGF1R,PTK2B,BCL2,TEK,RAC1,SCARB1,THBS1,PIK3R1,COL18A1,SELP,IL6,MAP2K1,IL8,SMAD3,IGF1,PLG,KDR,JAK2,HDAC9,IGFBP3,HTR2C,SST,F2R,LYN,ACTN4,ACTN1,CDKN1B
regulation of phosphate metabolic process(MAPK)	37.22788	GNA13,ADCY4,ADCY1,GNA15,CASR,ADCY2,ADORA3,PDGFB,ADCY8,EFNA1,ADCY5,ADCY6,EDN1,LPAR3,LPAR2,TLR4,LPAR1,GDNF,ADORA1,TGFB1,PRKACG,APOA4,S1PR2,AGTR1,AGTR2,S1PR1,APOA1,LTB4R,APOE,S1PR4,GALR2,PLA2G1B,IL1B,FRS2,INSR,SYK,EGFR,C5AR1,ACTN4,RELA,IRS1,NTSR2,EP300,HIF1A,F2,SMARCA4,TRAF2,CAV1,GNAI2,DRD3,ADORA2A,DRD2,ERBB2,DRD4,APOC2,BDKRB1,VDR,P2RY6,KRAS,P2RY2,RAC1,P2RY1,ADRA2A,SCARB1,ADRA2C,ADRA2B,EGF,VAV3,MAP2K1,MET,SMAD3,HGF,VAV2,PTPN11,PSMC5,AVPR1B,AVPR1A,ABL1,OPRM1,CCKAR,INS-IGF2,TACR1,CXCR2,SDC4,EDNRA,AKT1,EDNRB,NMUR1,NMUR2,SHC1,PAK1,MYC,PLCB2,CDK1,CCKBR,ELANE,TP53,CDK5,CCND1,NCOA3,CCND3,CHRM2,LCK,UBC,ADAM17,HCRT,PRKCZ,CCK,TNF,FPR1,KITLG,KIT,TPM1,GCGR,CALCA,PTK2B,BCL2,PTH,THBS1,IL4,IL6,IL5,STAT1,LRP1,ADCY9,BAX,LRP8,JAK2,HTR2B,HTR2C,IL2,HTR2A,F2R,IL6ST,APP,SOCS1,VEGFC,JUN,PDGFRB,ITGB2,BDKRB2,SMAD4,CDKN1B,RGS4,FABP4,MTOR,CASP3,PRKCA,LYN,RB1,IL6R,PRKCE,PRKCD,CSF2,IL3,IGF1,CDC25C,ADIPOQ,YWHAE,IGFBP3,PPARG,FGFR1,GRB2,OXTR,MAPKAPK2,RET,MAPK1,CRKL,NPHS1,MAPK9,MAPK8
response to wounding	33.60882	F2RL2,GNA13,A2M,ADORA3,PDGFB,F13A1,F2RL1,RPS6KB1,TLR4,ADORA1,TGFB1,CXCL10,S1PR3,CD44,LTB4R,CTGF,SERPINE1,IL1B,RELA,RXRA,GRIN2A,F7,CCR7,HIF1A,CD36,CCR5,CCR3,F2,PDGFRA,PLA2G2D,CCL2,CYSLTR1,ADORA2A,ERBB3,ERBB2,ITGB2,BDKRB1,ITGB3,BDKRB2,CCL5,P2RY1,RAC1,SCARB1,BLNK,FN1,PTPN6,MAP2K1,SMAD3,CXCL13,HDAC9,PLAU,PPARA,INS-IGF2,TACR1,CXCR1,NFKB1,CXCR2,AKT1,FOS,CASP6,GPX1,CASP3,GSN,KNG1,LYN,IL6R,GAL,CDK5,WAS,SDC1,ALOX15,ADAM17,PTAFR,TF,YWHAZ,TNF,C3,CXCL9,TAC1,FPR2,TPM1,TNFRSF1A,CCL21,BCL2,HRH4,SERPINC1,THBS1,NFATC3,NOX4,SELP,IL6,CEBPB,IL5,IL2RA,IL8,OLR1,ANXA1,CCL19,IGF1,PLG,STAT3,TFRC,BAX,JAK2,CD14,F2R,PPARG,SNCA,APOA4,GRIN2B,LTF,NOS2,C5AR1,ESR2,CD83,CCR6,PPBP,PLA2G2A,CCK,FGR,DRD4,ITGB1,IL4,HCK,PENK
response to endogenous(hormone) stimulus	31.7436	ADCY4,ADCY1,A2M,ADCY2,PDGFB,PTGS2,IL6ST,ADCY8,ADCY5,ADCY6,SNCA,RPS6KB1,TLR4,MMP3,TGFB1,B2M,CTNNB1,GOT2,PRKACG,HTR1B,APOB,GOT1,GRIN2B,CD44,APOE,PLA2G1B,IL1B,GNG2,INSR,EGFR,IRS2,RELA,RXRA,SOCS1,GRIN2A,TAT,IRS1,RETN,MAPK1,EP300,JUN,PDGFRA,COL1A1,CAV1,CCL2,DRD3,ADORA2A,GRB2,ERBB3,DRD2,ERBB2,DRD4,ADRBK1,BDKRB1,BCL2L1,CCL5,TRH,SRC,KRAS,SCARB1,GNRH1,MAP2K1,SMAD3,PTPN11,PLA2G4A,ADRA1B,AVPR1A,FABP4,PTPN1,MTOR,HDAC9,SST,PPARA,LDLR,INS-IGF2,BCAR1,TACR1,PPARG,FASLG,AKT1,EDNRA,FOS,CASP6,CASP3,GSN,NMUR2,SHC1,ANGPT1,MYC,PRKCA,AR,LYN,ESR1,IL6R,ESR2,GAL,MMP13,CDK5,CD83,SDC1,CCND1,NCOA6,ADAM17,PTAFR,PLA2G5,TF,TNF,TAC1,OXTR,GCGR,STAT6,TNFRSF1A,IGF1R,HRH3,PTK2B,BCL2,REN,SH2B2,THBS1,PIK3R1,SELP,IL6,CREB1,PTPRA,NPY1R,STAT1,ADIPOQ,STAT3,GCG,LEP,ADCY9,ID2,TFRC,FYN,MAPK14,JAK2,HTR2C,CD14,F2R
cell migration	30.07342	GNA13,CCKAR,PTGS2,PDGFB,BCAR1,RPS6KB1,CXCR2,GDNF,CD2AP,TGFB1,VCL,EDNRB,GPX1,APOB,APP,APOA1,GAB2,CXCR5,CD44,CTGF,LTB4R,PLA2G1B,IL1B,SYK,PRKCA,RET,EFNB1,ELANE,ESR2,IL6R,CDK5,VASP,VEGFC,CCR6,HIF1A,UBC,PDGFRB,ADAM17,MAPK8,WASL,CCL2,TNF,CCK,DRD2,ERBB2,VIM,FPR1,KITLG,ITGB2,FPR3,GIPC1,FPR2,KIT,CCL5,TPM1,ITGB1,EPHB1,ITGAM,SRC,VCAM1,CALCA,PTK2,PTK2B,RAC1,ADRA2A,PAFAH1B1,SCARB1,SH2B1,THBS1,FN1,ACTB,SELP,IL6,VAV3,PLA2G10,MAP2K1,IL8,MET,ANXA1,IGF1,ITGA4,VAV2,YWHAE,STAT3,KDR,ITGA6,NPY,FYN,MAPK14,BAX,LRP8,JAK2,PYY,PLAU
chemotaxis	29.05627	ADCY1,CASR,HRAS,PTGS2,PDGFB,ADCY8,SNCA,RPS6KB1,GDNF,CXCL10,S1PR2,HTR1B,APP,AGTR2,HTR1A,GRIN2B,GALR3,GALR2,PLA2G1B,IL1B,SYK,MYO6,C5AR1,GRIN2A,MAPK1,CCR8,CCR7,CCR6,PPBP,CCR5,CCR3,JUN,PDGFRB,CCL2,DRD3,CYSLTR1,ADORA2A,DRD2,DRD4,ITGB2,CCL5,TRH,ITGAM,KRAS,RAC1,GRPR,MAP2K1,MET,PENK,NPY,CXCL13,ADRA1B,AVPR1A,PYY,PLAU,MTNR1A,OPRM1,CCKAR,MCHR1,TACR1,CXCR1,CXCR2,EDNRB,FOS,NMUR2,PLCB1,PRKCA,CCKBR,ESR2,IL6R,GAL,CDK5,PTAFR,PRKCZ,HCRT,CCK,CXCL5,OPRK1,NPY2R,FPR1,CXCL9,OXTR,TAC1,FPR3,FPR2,KIT,NPAS2,HRH3,CCL21,REN,BCL2,PAFAH1B1,NMS,NMU,IL4,IL6,IL8,CCL19,NPY1R,STAT3,LEP,GCG,NRAS,FYN,MAPK14,HTR2C,HTR2A,OPRD1
regulation of lipase and hydrolase activity	28.85465	GNA13,HCRT,CCKAR,PRKCZ,GNA15,DRD2,TACR1,EDN1,LPAR3,APOC2,LPAR2,CXCR2,LPAR1,CALCA,APOA4,EDNRA,AGTR1,EDNRB,P2RY6,AGTR2,S1PR1,LTB4R,NMUR1,P2RY2,S1PR4,NMUR2,P2RY1,APOC3,PLA2G1B,PLCB2,EGFR,C5AR1,CCKBR,NTSR2,CHRM2,AVPR1B,AVPR1A,JAK2,HTR2B,HTR2C,HTR2A,F2R,CASR,APOA1,MYC,TP53,LCK,F2,TNF,CCK,TPM1,PTK2B,MAP2K1,SMAD3,STAT1,BAX,SNCA,GPX1,CAV1,ADORA2A,PAFAH1B1,VAV3,NR4A1,SIRT1,VAV1,MTOR,ERBB2,IGF1R,GSN,PIK3CA,IGF1,NPY,PTAFR
second-messenger-mediated signaling	27.44655	OPRM1,CCKAR,MCHR1,ADCY4,CASR,ADCY1,GNA15,ADCY2,ADCY8,ADCY5,TACR1,EDN1,ADCY6,LPAR3,CXCR2,ADORA1,EDNRA,EDNRB,AGTR1,S1PR3,HTR1B,AGTR2,HTR1A,S1PR1,GSN,APOE,NMUR1,LTB4R,NMUR2,S1PR4,GALR2,PIK3CA,NOS2,HTR1D,HTR5A,HTR1F,HTR1E,EGFR,CCKBR,NTSR2,FLNA,SSTR4,SSTR5,SSTR2,SSTR3,CHRM4,CD36,SSTR1,CHRM2,CCR3,PTAFR,HCRT,CCL2,TNF,DRD3,GNAI2,ADORA2A,DRD2,OPRK1,NPY2R,ERBB2,DRD4,FPR1,GCGR,CALCA,IGF

		1R,P2RY6,HRH3,P2RY2,PTH,P2RY1,IL8,OPRL1,IGF1,NPY1R,RGS1,ADCY9,NPY,MTNR1B,TBXA2R,AVPR1A,ADRA1B,GRK5,HTR2B,HTR2C,MTNR1A,HTR2A,F2R,OPRD1,GNAI3,ADORA3,ABCA1,GALR3,ADRA2A,INS-IGF2,SNCA,PPARG,NFKB1,AKT1,GPX1,CASP3,APOA1,IL1B,PRKCA,TP53,RB1,UBC,PRKCZ,CAV1,APOC3,NR4A1,SIRT1,PSMC5,CDKN1B,RGS4,FABP4,INSR,PRKCD,SUMO1,ID2,JAK2
positive regulation of cell communication and signal transduction	22.03047	HRAS,PTGS2,IL6ST,INS-IGF2,TACR1,SNCA,LPAR3,FASLG,LPAR2,TLR4,LPAR1,TGFB1,CTNNB1,GPX1,IL1B,INSR,PRL,SYK,EGFR,LYN,RELA,ESR1,IL6R,F7,IRS1,FLNA,HIF1A,NCOA3,LCK,VEGFA,UBC,ADAM17,CSF2,PRKCZ,HCRT,CAV1,CCL2,TNF,DRD3,ADORA2A,ERBB3,ERBB2,OXTR,TAC1,GIPC1,KIT,SRC,ARNT,TNFRSF1A,KRAS,SOS1,RAC1,PTH,THBS1,EGF,NMU,IL4,IL3,IL6,IL5,SMAD4,IGF1,ADIPOQ,PTPN11,LEP,NRAS,JAK2,MTOR,HTR2B,IL2,F2R,EDN1,AKT1,GRIN2B,PAK1,SOCS1,AMBP,DRD2,PTK2B,REN,HDAC3,NCOR1
regulation of secretion	21.58592	MCHR1,INS-IGF2,TACR1,SNCA,EDN1,LPAR3,GDNF,ADORA1,TGFB1,AKT1,EDNRA,GRIN2B,APOE,PLA2G1B,IL1B,INSR,SYK,PRKCA,IRS2,ACTN4,IRS1,CDK5,FLNA,VEGFC,F2,PRKCZ,HCRT,CAV1,TNF,ADORA2A,DRD2,ERBB3,C3,DRD4,TAC1,OXTR,ADRBK1,BDKRB1,TRH,P2RY1,NMU,PIK3R1,IL6,PLA2G10,CREB1,CBL,SMAD4,SMAD3,ADIPOQ,PTPN11,PLA2G4A,BAX,AVPR1B,AVPR1A,JAK2,HTR2C,PDZK1,F2R,IL2,DRD3,AGTR1,HTR1B,APOA1,HRH3,NOS2,EGF,MYO6,NPY1R,LEP,GCG,SSTR5,HTR2A,KHDRBS1,SIRT1
response to steroid hormone stimulus	21.39311	A2M, PTGS2, PDGFB, LDLR, PPARG, RPS6KB1, TGFB1, CTNNB1, FOS, CASP6, HTR1B, GOT1, IL1B, ANGPT1, INSR, PRKCA, RELA, RXRA, SOCS1, ESR1, IL6R, ESR2, GAL, TAT, MMP13, MAPK1, CCND1, SDC1, EP300, PDGFRA, COL1A1, CAV1, TNF, CCL2, ERBB2, OXTR, TRH, CCL5, SRC, KRAS, BCL2, THBS1, IL6, MAP2K1, GNRH1, NPY1R, ADIPOQ, STAT3, PLA2G4A, AVPR1A, FABP4, SST CAV1, A2M, TNF, CCL2, PTGS2, RPS6KB1, TRH, CCL5, SRC, FOS, HTR1B, KRAS, GOT1, BCL2, IL1B, INSR, PRKCA, IL6, MAP2K1, GNRH1, RXRA, IL6R, TAT, ADIPOQ, SDC1, CCND1, PLA2G4A, EP300, AVPR1A, FABP4, COL1A1 A2M, CAV1, TNF, CCL2, PTGS2, RPS6KB1, TRH, CCL5, FOS, GOT1, KRAS, BCL2, IL1B, INSR, PRKCA, IL6, MAP2K1, RXRA, IL6R, TAT, ADIPOQ, SDC1, CCND1, PLA2G4A, EP300, AVPR1A, FABP4
positive regulation of protein amino acid phosphorylation	21.33245	PDGFB,INS-IGF2,IL6ST,EDN1,TLR4,TGFB1,EDNRA,EDNRB,APOA1,IL1B,PAK1,INSR,SYK,PRKCA,EGFR,LYN,SOCS1,IL6R,PRKCE,PRKCD,VEGFC,CCND1,CCND3,JUN,F2,ADAM17,PDGFRB,CSF2,PRKCZ,CAV1,TNF,CCK,KITLG,BDKRB1,ITGB2,BDKRB2,PTK2B,BCL2,EGF,IL4,IL3,IL6,IL5,SMAD4,IGF1,ADIPOQ,BAX,JAK2,MTOR,IGFBP3,IL2,ADORA1,THBS1,A2M,SNCA,NFKB1,APOA4,AKT1,AGTR1,APP,APOE,CDK1,CDK4,PPARGC1A,CDK5,MAPK1,EP300,UBC,GIPC1,ITGB3,TIMP1,ITGAV,PLA2G10,YWHAE,PSMC5,CBLB
blood circulation(regulation of blood pressure)	19.23596	PPARA,CAV1,PTGS2,DRD3,ADORA2A,DRD2,TACR1,PPARG,EDN1,TAC1,OXTR,BDKRB1,ADRBK1,BDKRB2,POMC,ADORA1,GCGR,TPM1,CXCL10,CALCA,EDNRA,GPX1,AGTR1,EDNRB,AGTR2,HRH3,APOE,REN,CAMK2D,NOS2,KNG1,OLR1,NPY1R,STAT1,ADIPOQ,NPY,NTS,VEGFA,AVPR1B,COL1A2,ADRA1B,AVPR1A,HTR2B,NPPA
regulation of programmed cell death	16.81408	HRAS,PTGS2,MMP9,SNCA,TLR4,GDNF,ADORA1,TGFB1,APP,AGTR2,CD44,APOE,PIK3CA,IL1B,EGFR,ACTN4,RELA,RXRA,GRIN2A,ACTN1,F7,MAPK1,SSTR3,JUN,VEGFA,F2,MAPK9,MAPK8,TRAF2,CCL2,ADORA2A,ERBB3,ERBB2,BCL2L1,BDKRB2,SRC,VDR,KRAS,ALB,RAC1,COL18A1,VAV3,GNRH1,SMAD3,NR4A1,HGF,VAV2,VAV1,PLA2G4A,HDAC3,PSMC5,CDKN1B,HDAC1,ABL1,SST,INS-IGF2,BCAR1,MITF,FASLG,NFKB1,AKT1,EDNRB,CASP6,GPX1,CASP3,MYC,KNG1,PRKCA,CDK1,ESR1,TP53,ESR2,IL6R,PRKCE,GAL,CDK5,LCK,UBC,ADAM17,CSF2,PRKCZ,YWHAZ,CCK,TNF,KIT,IGF1R,SOS1,BCL2,PTH,SOS2,INPP5D,THBS1,RASA1,IL4,IL3,IL2RB,IL6,CEBPB,IL2RA,CREB1,ANXA1,IGF1,STAT1,ADIPOQ,SIRT1,YWHAE,PLG,NRAS,BAX,JAK2,IGFBP3,IL2,F2R,DNM2,E2F1,TSPO,CXCR2,CTNNB1,IAPP,GSN,PAK1,AR,DCTN1,EP300,PLA2G6,ITGB2,TNFRSF1A,DOCK1,PTK2B,PTPN6,OLR1,BRE,CD14,HTR2A
response to nutrient levels	16.12505	CCKAR,PPARA,CAV1,A2M,CCK,CCL2,GNAI2,PTGS2,IL6ST,PPARG,RPS6KB1,GCGR,TGFB1,AKT1,VDR,FOS,CD44,GSN,ALB,BCL2,SERPINC1,IL1B,ANGPT1,INSR,SREBF1,LYN,RELA,RXRA,TP53,STAT1,SIRT1,ADIPOQ,PPARGC1A,LEP,PLA2G4A,CCND1,SSTR2,TFRC,NPY,ITGA6,SSTR1,JUN,AVPR1A,MTOR,COL1A1,LRP2,SST,KLF4
positive regulation of response to stimulus	15.68709	PPARA,MYOD1,A2M,PTGS2,PDGFB,IL6ST,INS-IGF2,F2RL1,EDN1,PPARG,TLR4,ADORA1,TGFB1,EDNRA,AGTR1,GPX1,S1PR1,APOE,SERPINE1,PRKCA,KNG1,ELANE,IL6R,F7,F2,VEGFA,PLA2G2A,ADAM17,CAV1,CCK,DRD3,ADORA2A,DRD2,C3,TAC1,CCL5,TNFRSF1A,SERPINC1,FCER1G,THBS1,SELP,IL6,IL2RA,IL8,SMAD3,ADIPOQ,PLG,KDR,ANXA2,LEP,PLA2G4A,NPY,FABP4,JAK2,MTOR,PLAU,F2R,IL2,TRAF2,TNF,BCAR1,SRC,B2M,PLA2G1B,IL1B,SH2B2,SYK,LYN,RELA,IRS1,MAPK1,FYN,BRE,CD79A
response to bacterium	15.51425	CCL2,PTGS2,SNCA,FASLG,TAC1,BDKRB1,TLR4,RPS6KB1,CCL5,B2M,FOS,TNFRSF1A,IL1B,SCARB1,SELP,IL6,RELA,SOCS1,IL6R,STAT1,MAPK1,PLA2G4A,MAPK14,JUN,ADAM17,CD14,PTAFR,F2R,TNF,FGR,GPX1,LTF,NOS2,HCK,PPBP,PLA2G2A,WASL
regulation of transmission of nerve impulse	14.96775	HRAS,ADORA3,PTGS2,IL6ST,INS-IGF2,TACR1,SNCA,EDN1,RPS6KB1,SDC4,GDNF,ADORA1,APOA4,EDNRB,AGTR1,AGTR2,HTR1B,APOA1,GRIN2B,APOE,NMUR2,IL1B,NOS2,PRKCA,EGFR,MYO6,GRIN2A,CDK5,SSTR2,CHRM3,CHRM2,UBC,RAB5A,TF,PRKCZ,HCRT,CAV1,GNAI3,CCL2,TNF,CCK,DRD3,GNAI2,ADORA2A,DRD2,DRD4,OXTR,TAC1,ADRBK1,GIPC1,KIT,BDKRB2,TPM1,CALCA,KRAS,NMU,MAP2K1,NPY1R,PTPN11,LEP,NRAS,AVPR1B,TBXA2R,AVPR1A,ADRA1B,JAK2,HTR2C,IL2,F2R,HTR2A
vasculature development	14.46437	GNA13,CAV1,EDN1,CTNNB1,CALCA,AKT1,EDNRA,GPX1,AGTR2,APOB,PTK2,S1PR1,CD44,CTGF,PTK2B,APOE,ITGAV,IL1B,ANGPT1,NOS2,ADRA2B,EGF,FGF1,THBS1,FIGF,RASA1,COL18A1,KLF5,SELP,IL8,ITGA4,KDR,ANXA2,VEGFC,HDAC3,CRKL,HIF1A,MAPK14,JUN,BAX,VEGFA,COL1A2,ADRA1B,COL1A1,PLAU,SMARCA4
cell-cell signaling	14.09115	EFNA1,INS-IGF2,EDN1,SNCA,LPAR3,FASLG,GDNF,ADORA1,CXCL10,CTNNB1,HTR1B,APP,GRIN2B,IAPP,APOE,NMUR2,GALR3,GALR2,IL1B,HTR1D,FGF1,FRS2,HTR1F,NPFF,HTR1E,SYK,PRKCA,AR,MYO6,LYN,EFNB1,NPBWR1,GRIN2A,ESR2,PDYN,GAL,CDK5,MAPK1,CD86,SSTR2,SSTR3,CCR5,SSTR1,UBC,HCRT,YWHAZ,CXCL5,DRD3,ADORA2A,DRD2,GRB2,OPRK1,CAMK2G,FFAR1,DRD4,CXCL9,OXTR,TAC1,GIPC1,ITGB2,AKAP9,TRH,CCL5,POMC,EPHB1,CALCA,PTK2,HRH3,CCL21,TEK,PTH,PAFAH1B1,ADRA2C,ADRA2B,MLLT4,IL3,IL6,GNRH1,HGF,LEP,PNOC,NPY,CXCL13,MTNR1B,ADRA1B,PYY,LRP2,HTR2C,SST,IL2,HTR2A,ERBB2,TGFB1,S1PR1,NMUR1,OPRM1,HRAS,ADCY1,PTGS2,ADCY8,TACR1,RPS6KB1,GPX1,FOS,CASP3,AGTR2,TGFBI,PLCB1,PLCB2,MYC,C5AR1,CCKBR,NTSR2,ARRB2,ARRB1,JUN,COL1A1,PRKCZ,BDKRB1,KIT,KRAS,RAC3,GRPR,NMU,COL18A1,OPRL1,NPY1R,NRAS,CDKN1B,PENK,FYN
response to abiotic stimulus(radiation, light)	13.92058	HRAS,TACR1,PPARG,RPS6KB1,TGFB1,AKT1,CASP6,FOS,GPX1,CASP3,APP,GRIN2B,IL1B,MYC,PRKCA,EGFR,LYN,RELA,ELANE,GRIN2A,TP53,IL6R,MMP13,CDK5,CCND1,ARRB1,JUN,MAPK8,COL1A1,CLOCK,CAV1,CCL2,TNF,DRD3,DRD2,TAC1,BCL2L1,KIT,BDKRB2,TRH,CALCA,KRAS,PTK2B,BCL2,RAC1,NPFFR2,THBS1,NMU,COL18A1,STAT1,NRAS,PLA2G4A,FYN,POLD1,BAX,AVPR1B,BRE,AVPR1A,ADRA1B,LRP2,SST

response to inorganic substance(oxidative stress hydrogen peroxide)	11.76315	A2M,CAV1,CASR,PTGS2,SNCA,ADRBK1,BCL2L1,TPM1,PXN,CTNNB1,APOA4,FOS,GPX1,CASP6,APOB,GSN,PTK2B,APOE,BCL2,SERPINE1,THBS1,INSR,ACTB,PRKCA,EGFR,OLR1,RELA,RXRA,STAT1,TAT,PLA2G4A,CCND1,SDC1,EP300,TFRC,JUN,PDGFRA,AVPR1A,MAPK9,MAPK8,COL1A1,TLR4,CCL5,ARNT,GPX5,MAP2K1,HIF1A,JAK2
regulation of lipid metabolic process	10.06774	PPARA,CAV1,TNF,DRD3,PDGFB,INS-IGF2,SNCA,PPARG,APOC2,NFKB1,ADORA1,TGFB1,AKT1,APOA4,AGTR1,APOB,APOA1,APOE,RAC1,APOC3,IL1B,IRS2,VAV3,RB1,VAV2,GAL,IRS1,ADIPOQ,PPARGC1A,LEP,PLA2G4A,AVPR1A,MTOR,INSR
regulation of peptidyl-tyrosine phosphorylation(JAK-STAT)	9.705443	CSF2,PRKCZ,CAV1,CCK,PDGFB,INS-IGF2,IL6ST,KITLG,ITGB2,TLR4,EGF,SYK,IL4,EGFR,PRKCA,IL3,IL6,IL5,LYN,SOCS1,IGF1,IL6R,PRKCE,PRKCD,ADIPOQ,PDGFRB,JAK2,MTOR,IL2,PRL,F2R
vesicle-mediated transport	9.202748	HRAS,CAV1,GNAI3,LDLR,DRD3,ADORA2A,SNCA,BCL2L1,ABCA1,ADORA1,GDNF,APP,KRAS,DOCK1,APOE,ITGAV,BCL2,RAC1,FCER1G,PAFAH1B1,SCARB1,THBS1,SAR1B,EGFR,PLD2,MYO6,VAV3,HCK,ELANE,TP53,LMNA,VAV1,CDK5,NRAS,LRP1,CD36,ARRB2,TFRC,ARRB1,BAX,RAB5A,SEC13,LRP8,LRP2,DNM1,SH3GL2,CD14,DNM2,CTNNB1,GSN,LYN,WAS,FLNA,YWHAZ,CCL5,GCGR,SRC
leukocyte migration	9.200083	PRKCA,SELP,IL6,TNF,CCL2,PDGFB,IL8,ELANE,ITGB2,CXCR2,IL6R,CCL5,ITGAM,VCAM1,EDNRB,ITGA6,PLA2G1B,IL1B,SYK,PDGFRB
regulation of oxidoreductase and monooxygenase activity(NO)	9.016985	EGFR,TNF,INS-IGF2,SNCA,EDN1,NFKB1,GDNF,AKT1,AGTR1,VDR,AGTR2,HIF1A,KRAS,APOE,IL1B,SCARB1,ABL1,JAK2,INSR
positive regulation of DNA metabolic process(replication)	8.821448	IL4,CSF2,IL3,HRAS,PDGFB,INS-IGF2,IGF1,KITLG,TGFB1,STAT6,IGF1R,JUN,PDGFRA,PLA2G1B,BRE,SHC1,INSR,IL2,HCRT,TP53,MYC
immune system development and activation	8.723814	GNA13,CSF2,YWHAZ,TNF,ADORA2A,INS-IGF2,SNCA,EDN1,TLR4,CXCR2,ITGB1,TGFB1,ITGAM,CTNNB1,VCAM1,CXCR5,BCL2,TCF3,PIK3R1,BLNK,SYK,EGFR,IL4,IL6,LYN,IL8,TP53,SMAD3,ITGA4,VAV1,WAS,PRKCD,CD86,CBLB,CCND3,FYN,BAX,LCK,F2,PDGFRA,ADAM17,CD79A,HDAC9,LCP2,F2R,IL2,MMP9,PPARG,KITLG,KIT,TIMP1,RB1,KDR,CRKL,SP1,ID2,CXCL13,VEGFA,NCOA6,JAK2
negative regulation of protein metabolic process	7.970606	PRKCZ,CAV1,A2M,INS-IGF2,SNCA,GIPC1,BDKRB1,NFKB1,ITGB3,BDKRB2,TGFB1,TIMP1,APOA4,ITGAV,THBS1,INSR,PRKCA,RELA,SOCS1,GRIN2A,CDK5,PRKCD,YWHAE,FLNA,PSMC5,JUN,BAX,UBC,IGFBP3,IL2,CDKN1B
wound healing,coagulation,hemostasis	7.795072	F2RL2,GNA13,PPARA,PDGFB,ADORA2A,INS-IGF2,ERBB3,ERBB2,F13A1,F2RL1,ITGB3,TPM1,TGFB1,GPX1,CD44,GSN,P2RY1,SERPINE1,SERPINC1,IL1B,SCARB1,FN1,KNG1,IL6,SMAD3,IGF1,F7,WAS,PLG,SDC1,HIF1A,CD36,F2,PDGFRA,ADAM17,PLAU,F2R,DRD2,EDN1,ANXA2,APLNR
blood coagulation	7.688673	KNG1,SELP,CAV1,PDGFB,F2RL1,EDN1,TLR4,PLG,ANXA2,APOE,SERPINE1,F2,THBS1,PLAU,F2R
response to alkaloid(morphine)	7.465815	DRD3,ADORA2A,DRD2,TACR1,RELA,DRD4,PPARG,OXTR,TAC1,CDK5,EDNRA,HTR1B,PTK2B,BCL2,ADRA1B,IL1B,HTR2C,MYC
response to organic nitrogen	7.331613	CCL2,DRD3,PTGS2,LYN,ADORA2A,DRD2,RELA,DRD4,GRIN2A,OXTR,RPS6KB1,BCL2L1,MMP3,CASP6,CCND1,PLA2G4A,PTK2B,ADRA1B,MTOR,SST,GSN,BCL2,BAX,SRC
regulation of ion transport	7.330547	MCHR1,HCRT,CAV1,CASR,TNF,DRD3,PTGS2,GNAI2,DRD2,ADORA2A,DRD4,LPAR3,BDKRB1,ADORA1,TGFB1,CALCA,EDNRA,AKT1,BCL2,P2RY1,PLA2G1B,ACTN4,LYN,BAX,F2,PDZK1,F2R,HTR2A,CCK,TACR1,TAC1,CCL5,PLA2G4A,AVPR1B,AVPR1A
negative regulation of locomotion	7.056924	PDGFB,ADORA2A,DRD2,ELANE,ADORA1,TPM1,PLG,TGFB1,VCL,AGTR2,APOE,BCL2,RRAS,THBS1,HTR2C,IGFBP3,ACTN4,ACTN1,CDKN1B
neurological system process(related to learning, memory, cognition, sensory perception)	7.014692	PRKCZ,ADCY1,HRAS,DRD3,PTGS2,ADCY8,DRD2,TACR1,OXTR,TAC1,RPS6KB1,KIT,FOS,APP,KRAS,GRIN2B,HRH3,GALR3,GALR2,GRPR,IL1B,PAFAH1B1,PLCB1,PRKCA,GRIN2A,ESR2,CDK5,NRAS,FYN,JUN,ADRA1B,HTR2A,OPRM1,SNCA,LPAR3,ADORA1,TGFB1,CTNNB1,GPX1,HTR1B,CASP3,AGTR2,S1PR1,IAPP,APOE,NMUR1,NMUR2,TGFBI,HTR1D,PLCB2,MYC,HTR1F,NPFF,HTR1E,MYO6,C5AR1,CCKBR,NPBWR1,PDYN,NTSR2,MAPK1,ARRB2,ARRB1,UBC,COL1A1,HCRT,ADORA2A,OPRK1,ERBB2,DRD4,GIPC1,AKAP9,BDKRB1,CALCA,PTK2,RAC3,NMU,COL18A1,OPRL1,NPY1R,CDKN1B,PNOC,NPY,PENK,MTNR1B,SST,HTR2C
regulation of muscle contraction	7.002825	PRKCA,CAV1,PTGS2,MAP2K1,TACR1,EDN1,OXTR,ADRBK1,SDC4,TPM1,CALCA,SSTR2,CHRM3,CHRM2,NMUR2,TBXA2R,NMU,F2R,BDKRB2,NPY1R,AGTR1,AVPR1A,HTR2A
regulation of synaptic plasticity(related to learning and visual behavior)	6.867867	HRAS,DRD3,DRD2,TACR1,GRIN2A,TAC1,KIT,CDK5,FOS,NRAS,APP,KRAS,GRIN2B,HRH3,FYN,JUN,ADRA1B,PLCB1,MYO6,PTGS2,ADORA2A,SNCA,GIPC1,ADORA1,APOE,UBC,RAB5A
lipid homeostasis(transport, location, and secretion)	6.278148	LPL,CAV1,IRS2,LDLR,INS-IGF2,PPARG,APOC2,ABCA1,APOA4,APOB,APOA1,GOT1,APOE,APOC3,FABP4,SCARB1,GOT2,PPARA,CD36,DRD3,DRD2,NMUR2,DRD4,ANXA1,PLA2G1B,BDKRB2,PLA2G5,OXTR,NMU,TNF,IL1B,PLA2G10,PDZK1
regulation of catabolic process（glucose and lipid）	6.217569	PPARA,TNF,INS-IGF2,APOC2,GIPC1,ADORA1,ARNT,TIMP1,AKT1,APOA4,APOE,APOC3,IL1B,INSR,IRS2,RELA,GRIN2A,IGF1,ARNTL,IRS1,FLNA,CBLB,HIF1A,ADRA1B,MTOR,ADIPOQ,PPARGC1A,LEP,GRB2,BCAR1,PTPRA,IGF1R,SHC1,SH2B2,PTPN1,PIK3R1,PRKCA,PRKCZ,ERBB3,NOS2,FFAR1,PPARG,STAT3,PTPN11,SSTR5,SERPINE1
regulation of immune system process(cell activation and proliferation)	6.213931	TRAF2,IL6ST,C3,TACR1,BCAR1,F2RL1,TAC1,TLR4,BDKRB1,TGFB1,B2M,STAT6,VCAM1,IL4R,PLA2G1B,FCER1G,IL1B,IL2RG,SH2B2,INPP5D,NOS2,THBS1,TCF3,SYK,IL4,IL6,IL2RA,IL5,LYN,RELA,EFNB1,F7,IL6R,MAPK1,CD83,CD86,CBLB,FYN,VEGFA,LCK,ADAM17,CD79A,IL2,PDGFB,ADORA2A,ERBB2,SNCA,CASP3,APOE,SELP,PLA2G10,GAL,JAK2,A2M,INS-IGF2,ADORA1,AMBP,GPX1
regulation of growth(cell size)	6.191604	MYOD1,DRD3,INS-IGF2,DRD2,BCAR1,PPARG,RPS6KB1,BDKRB1,TGFB1,AKT1,AGTR1,APP,AGTR2,PTK2,CD44,CTGF,APOE,PTK2B,BCL2,NMUR2,CAMK2D,PIK3CA,SHC1,PRL,INSR,CREB1,SOCS1,SMAD4,TP53,SMAD3,IGF1,RB1,NPY1R,ESR2,KAT5,CHPT1,STAT3,PTPN11,LEP,CDKN1B,EP30

		0,UBC,AVPR1A,ADAM17,IGFBP3,NPPA,IL2,FGFR2,FGFR1,PDGFB,EDN1,AR,CDK4,ADRA1B,MTOR,GSN,RAC1,RASA1
regulation of gene-specific transcription	6.159851	PPARA,TNF,INS-IGF2,PPARG,NFKB1,GDNF,CTNNB1,ARNT,VDR,PLA2G1B,INSR,TCF3,IL4,TBL1XR1,IL6,IL5,CREB1,RXRA,TP53,SMAD3,IGF1,ESR2,KAT5,SIRT1,PPARGC1A,HDAC3,CDKN1B,HIF1A,PSMC5,HDAC1,HDAC9,NCOR1,NCOR2,SMARCA4,E2F1,A2M,EDN1,SNCA,TGFB1,APOA4,MYC,PRKCA,KHDRBS1,CTBP1,RELA,ELANE,SOCS1,GRIN2A,TLE1,RB1,IL6R,PRKCD,CDK5,FLNA,NCOA2,JUN,UBC,PRKCZ,HCRT,CAV1,DRD3,BDKRB1,GIPC1,ITGB3,BCL2L1,BDKRB2,TIMP1,CALCA,SUMO1,ITGAV,INPP5D,THBS1,SMAD4,ADIPOQ,YWHAE,STAT3,ID2,BAX,ADRA1B,FABP4,IGFBP3,KLF4,IL2,PDGFB,EDNRA,HTR1B,APOE,DRD4,APOC3
regulation of hormone secretion(negative)	6.125202	TNF,DRD3,ERBB3,DRD2,INS-IGF2,DRD4,EDN1,NPY1R,TRH,ADORA1,ADIPOQ,PTPN11,APOA1,HRH3,IL1B,EGF,CREB1,LEP,GCG,SSTR5,JAK2,NOS2,NMU
regulation of response to external stimulus(chemotaxis)	6.100395	PDGFB,C3,IL6ST,F2RL1,TAC1,TLR4,CCL5,TGFB1,EDNRA,AGTR1,TNFRSF1A,S1PR1,FCER1G,THBS1,PRKCA,IL6,IL8,SMAD3,F7,IL6R,KDR,PLA2G4A,NPY,VEGFA,PLA2G2A,FABP4,ADAM17,JAK2,DRD3,ELANE,ESR2,HTR1A,SELP,BDKRB1,ADORA1,HIF1A,TNF,IL1B,YWHAZ,LYN,PLA2G1B,INPP5D,PRKCD,RELA,BCL2
neuron projection development	6.04777	CCKAR,PDGFB,LPAR3,LPAR1,CD2AP,GDNF,VCL,AKT1,APP,CD44,GALR2,PRKCA,EGFR,MYO6,RXRA,EFNB1,CDK5,VASP,UBC,FGFR1,CCK,DRD2,ERBB3,ADORA2A,ERBB2,ITGB4,TPM1,EPHB1,IGF1R,PTK2,RAC3,PTK2B,BCL2,RAC1,PAFAH1B1,SH2B1,KLF5,ACTB,IL6,VAV3,MAP2K1,PLA2G10,CREB1,VAV2,PTPN11,ITGA6,MAP2,JAK2,MTOR,RET,LEP,VEGFA,EFNA1,S1PR1,CEBPB,SMAD4,STAT3,EPHA2,TGFB1,CTNNB1,NFATC1,FN1,COL18A1,HGF,HIF1A,ITGB1,NOX4,BAX
muscle contraction(smooth muscle)	5.887009	EDNRA,KNG1,EDNRB,SSTR2,CHRM3,NMUR1,DRD2,TACR1,EDN1,BDKRB2,GAL,GDNF,TACR2,OXTR,IGF1,RPS6KB1,ADRBK1,TPM1,LTB4R,GALR2,CAMK2D,IL1B,
regulation of cell cycle	5.882962	CAV1,CCK,TNF,DRD3,INS-IGF2,C3,TACR1,EDN1,SNCA,APOC2,TAC1,OXTR,KIT,TPM1,TGFB1,AKT1,SUMO1,RAC1,IL1B,EGF,INSR,PIK3R1,PRKCA,ACTN4,CBL,SMAD4,SMAD3,IGF1,PPARGC1A,PLA2G4A,CDKN1B,MTOR,E2F1,PTGS2,ITGB1,APP,CASP3,BCL2,CAMK2D,MYC,TCF3,EGFR,CDK1,TP53,RB1,CDC25C,CDK4,CDK5,PTPN11,CCND1,HDAC3,CCND3,ID2,HDAC1,MAPK14,JUN,BAX,BRE,ADAM17,SMARCA4,AGTR2,JAK2,CTNNB1,S1PR1,GSN,RASA1,MAP2
cell cycle	5.770501	KHDRBS1,E2F1,EGFR,CAMK2G,POLE,POLA1,RB1,CDK4,CDC25C,ITGB1,AKT1,CCND1,APP,CDKN1B,POLD1,BCL2,CAMK2D,ADAM17,CAMK2B,ABL1,TCF3,NFATC1,DNM2,CD2AP,PAFAH1B1,CDK1,MAP2K1,HGF,DCTN1,RGS14,MIS12,PSMC5,UBC,TGFB1,CTNNB1,THBS1,MYC,IL8,TP53,SMAD3,HDAC3,NCOR1,CDK5,MAPK1,EP300,CCND3,MAPK3,ANXA1,RGS2
regulation of lipid storage	5.62488	LPL,PPARA,APOB,CD36,PLA2G10,ITGAV,PPARG,NFKB1,SCARB1,ABCA1,ITGB3
regulation of endothelial cell migration(blood vessel endothelial cell)	5.472484	VEGFC,AGTR2,PDGFB,APOE,VEGFA,EDN1,SCARB1,HDAC9,THBS1,PLG,TGFB1,DRD2,TPM1,ADORA1,VCL,BCL2,RRAS,IGFBP3
regulation of hormone secretion	5.355811	TNF,DRD2,CREB1,EDN1,NPY1R,TRH,ADORA1,ADIPOQ,PTPN11,LEP,GCG,SSTR5,IL1B,JAK2,NOS2,NMU
regulation of myeloid cell differentiation	5.333464	IL4,IL5,TNF,MITF,KITLG,TLR4,RB1,CCL5,ADIPOQ,ARNT,CTNNB1,CALCA,LEP,HIF1A,ID2,MAPK14,JUN,INPP5D
regulation of binding(TF-DNA)	5.33243	IL4,IL6,TNF,IL5,RELA,SMAD4,SMAD3,TLR4,SIRT1,PRKCD,FLNA,TGFB1,SUMO1,KRAS,EP300,NCOA3,ID2,BAX,BCL2,PLA2G1B,IL1B,JAK2,SMARCA4
cell-substrate adhesion	4.920021	ITGB4,ACTN1,ITGB2,ITGB3,CDK5,ITGB1,PXN,CTNNB1,CD44,ITGA6,PTK2B,CTGF,ITGAV,BCL2,TEK,FN1,ITGA2B
regulation of blood vessel size	4.821079	KNG1,CAV1,ADORA2A,EDN1,BDKRB2,EDNRA,AGTR1,EDNRB,GPX1,AGTR2,NTS,APOE,VEGFA,ADRA1B,NPPA
regulation of membrane potential	4.771247	PRKCZ,HCRT,CAV1,CCK,ADORA2A,SNCA,EDN1,GRIN2A,ADORA1,ADIPOQ,CDK5,GRIN2B,BCL2,JUN,ERBB2,TACR1,DRD4,TAC1,BCL2L1,NTSR2,TGFB1,BAX,TF
actin cytoskeleton organization	4.731574	PRKCZ,HRAS,PDGFB,BCAR1,TPM1,ITGB1,KRAS,GSN,RAC3,PTK2B,BCL2,RAC1,ADRA2A,PLA2G1B,SH2B2,PAFAH1B1,ACTN4,ACTN1,WAS,CDK5,FLNA,VASP,NRAS,WASL,ABL1,CRK,MYO6,CAV1,CTNNB1,KISS1,PTK2,APOE,PAK1,PLD2,RET,HDAC3,FGFR1OP,MAP2,PYY,NCOR1
apoptotic mitochondrial changes	4.711283	GPX1,CASP3,CCK,JUN,BCL2,BAX,TP53,SMAD3,BCL2L1,MYC,AKT1,YWHAZ,TSPO,SNCA,PPARGC1A
regulation of lipid catabolic process	4.640964	APOA4,AKT1,PPARA,IRS2,TNF,INS-IGF2,APOC3,IL1B,APOC2,MTOR,ADORA1,IRS1,PLA2G4A,PPARG,AVPR1A,PPARGC1A,ADIPOQ
positive regulation of Ras protein signal transduction	4.501229	NRAS,HRAS,KRAS,ERBB2,RAC1,IGF1,LPAR2,LPAR1
multicellular organismal response to stress	4.498211	CALCA,EDNRB,CCK,GRIN2B,PENK,TACR1,NMUR2,BCL2,DRD4,ADRA2A,TAC1,ESR2
regulation of cellular component biogenesis and organization	4.309742	CAV1,CCK,TNF,DRD3,INS-IGF2,C3,TACR1,EDN1,SNCA,APOC2,TAC1,OXTR,KIT,TPM1,TGFB1,AKT1,SUMO1,RAC1,IL1B,EGF,INSR,PIK3R1,PRKCA,ACTN4,CBL,SMAD4,SMAD3,IGF1,PPARGC1A,PLA2G4A,CDKN1B,MTOR,PRKCZ,PTK2,S1PR1,GSN,UBC,THBS1,RASA1,CTNNB1,MYC,CDC25C,MAP2
regulation of MAPKKK and JNK cascade	4.252365	CAV1,TNF,INS-IGF2,ERBB2,EDN1,LPAR3,LPAR2,TLR4,LPAR1,CTNNB1,AKT1,GRIN2B,PTK2B,REN,IL1B,PAK1,INSR,SYK,IL6,IL6R,AMBP,LEP,HDAC3,NCOR1,F2R,LYN,SIRT1,BRE,MTOR
regulation of lipid biosynthetic process	4.239257	TNF,PDGFB,INS-IGF2,APOC2,NFKB1,AKT1,APOA4,LEP,APOB,PLA2G4A,APOE,APOC3,AVPR1A,AGTR1,APOA1,GAL

regulation of peptidase activity(caspase and endopepetidase)	4.091797	CAV1,TNF,CCK,ADORA2A,SNCA,TP53,SMAD3,NR4A1,STAT1,GPX1,BAX,LCK,F2,MYC,F2R
JAK-STAT cascade(STAT)	4.042498	CCL2,PTK2B,IL6ST,SOCS1,F2,JAK2,STAT1,PRL,CDK5,STAT3,F2R
negative regulation of transmission of nerve impulse	4.031244	HCRT,HTR1B,GNAI3,PTGS2,GNAI2,DRD2,AVPR1A,ADORA1,HTR2A,EGFR,ADORA2A,OXTR,GPX1,IL1B,PPARGC1A,STAT3
skeletal system development	4.027581	EGFR,CASR,PTGS2,INS-IGF2,HSPG2,SMAD3,IGF1,MMP13,TGFB1,ALOX15,SP1,CTGF,BCL2,MAPK8,COL1A1,IGFBP3,FGFR1,MMP9,EDN1,CTNNB1,VDR,PTH,MYC,PRKCA,ANXA2,MAPK14,COL1A2,PDGFRA,PDGFRB
multicellular organismal homeostasis(anatomical structure)	4.021974	DRD2,ADORA1,PPARGC1A,STAT3,CTNNB1,KDR,GPX1,PLA2G4A,BAX,BCL2,VEGFA,RAC1,PTH,IL1B,PDGFRB,F2R,HTR2A,HIF1A,PLG
regulation of lipid kinase activity(PI3K)	3.994777	VAV3,RAC1,RB1,VAV2,IRS1,TGFB1,SH2B1,VCL
positive regulation of fatty acid metabolic process	3.950542	APOA4,PPARA,PLA2G4A,IRS2,PPARG,AVPR1A,APOC2,PPARGC1A,IRS1,ADIPOQ,AKT1,TNF,INS-IGF2,APOC3
regulation of protein transport	3.946114	RKCZ,TNF,DRD3,DRD2,INS-IGF2,ADORA2A,DRD4,TGFB1,AKT1,SUMO1,APOA1,BCL2,PLA2G1B,IL1B,EGF,PIK3R1,IL6,SMAD4,SMAD3,SIRT1,CDK5,FLNA,PTPN11,VEGFC,JAK2,IL2,KHDRBS1
regulation of heart contraction	3.905537	PRKCA,ADORA3,DRD2,EDN1,TAC1,ADRBK1,ADORA1,TPM1,AGTR2,CHRM2,AVPR1B,ADRA1B,AVPR1A,JAK2,NOS2,IL2,CALCA,AGTR1,DRD3,REN
mesenchymal cell and neural crest cell differentiation	3.736995	RET,EFNB1,EDN1,KITLG,HGF,GDNF,TGFB1,CTNNB1,EDNRA,EDNRB,HIF1A,BCL2,NFATC1
lymphocyte activation and proliferation	3.730129	INS-IGF2,ITGB1,TGFB1,ITGAM,CTNNB1,VCAM1,CXCR5,BCL2,TCF3,PIK3R1,BLNK,SYK,IL4,TP53,SMAD3,ITGA4,VAV1,PRKCD,WAS,CD86,CBLB,CCND3,FYN,BAX,LCK,ADAM17,CD79A,HDAC9,IL2
regulation of vesicle-mediated transport(endocytosis)	3.677367	PRKCA,CAV1,ACTN4,C3,SNCA,CBL,APOC2,CD2AP,ADIPOQ,TGFB1,APOC3,RAC1,SCARB1,CDK5,PLA2G4A,SYK
regulation of cell development(neurogenesis)	3.39116	DRD3,EFNA1,DRD2,INS-IGF2,EDN1,PPARG,KIT,TGFB1,CASP6,PTK2,APOE,BCL2,S1PR5,LYN,RELA,SMAD4,TP53,SMAD3,VEGFC,HDAC3,HDAC1,BAX,UBC,HDAC9,IGFBP3,PRKCA,TF,OXTR,IGF1,EGF
placenta development	3.313786	VDR,PLA2G4A,PTGS2,RXRA,IL1B,LRP2,INSR,TGFB1,AKT1,HIF1A,CEBPB,SP1,PPARG,EPOR,ARNT
regulation of lipid storage	3.284811	PPARA,HDAC1,ITGAV,PPARG,ITGB3,ADIPOQ,ABCA1
small GTPase mediated signal transduction(Ras and Rho)	3.24699	HRAS,VAV3,BCR,MAP2K1,ERBB2,IGF1,LPAR2,LPAR1,VAV2,VAV1,IQGAP1,NRAS,KRAS,S1PR1,PTK2B,SOS1,SOS2,RAC1,SH2B2,PAFAH1B1,MTOR,CRK,RAPGEF1,RASA1
transcription, DNA-dependent	3.229878	PPARA,MED23,NFKB1,POLR2C,ARNT,MAX,MYC,NFATC3,NFATC1,KLF5,AR,CEBPB,CEBPD,CREB1,TP53,ESR1,MED14,POLR1C,STAT1,PPARGC1A,GTF2B,HIF1A,PSMC5,MED17,NCOA6,NCOR1
gliogenesis	3.195237	EGFR,LYN,ADORA2A,ERBB3,GSN,PTK2B,ERBB2,PDGFRA,IGF1,CDK5,SMARCA4,CTNNB1
positive regulation of growth	3.155687	MYOD1,DRD2,INS-IGF2,CREB1,RPS6KB1,AKT1,EP300,PTK2B,BCL2,AVPR1A,ADAM17,INSR,IL2,EDN1,CDK4
macromolecular complex subunit organization	3.151449	HRAS,MED23,CD2AP,TGFB1,CTNNB1,APOA4,AGTR1,APOB,APOA1,APOE,GSN,ANGPT1,TGS1,KPNB1,MYC,INSR,SYK,TP53,MED14,GTF2B,WAS,IRS1,CDK5,PPARGC1A,FLNA,MED17,NCOA6,PLA2G2A,WASL,PRKCZ,TRAF2,CAV1,GRB2,APOC2,ABCA1,POLR2C,SRC,PXN,TNFRSF1A,IGF1R,PTK2,PTK2B,APOC3,RAC1,SCARB1,LPL,IL2RB,MAP2K1,CREBBP,SMAD4,SMAD3,ADIPOQ,MIS12,BAX
regulation of protein catabolic process	3.112045	TNF,INS-IGF2,RELA,GRIN2A,GIPC1,ADORA1,FLNA,TIMP1,AKT1,APOC3,ADRA1B,IL1B,MTOR,CBLB,APOE,ARNTL,A2M,EP300,THBS1
regulation of peptidyl-serine phosphorylation and leukocyte homestasis	3.093704	CAV1,IL6,BCL2,BAX,BDKRB2,TGFB1,AKT1,CASP3,IL2RA,SH2B2
secretion	3.012228	YWHAZ,DRD3,ADORA2A,TACR2,DRD2,CAMK2G,TACR1,EDN1,FFAR1,DRD4,OXTR,GIPC1,BDKRB2,ABCA1,CCL5,ADORA1,GCGR,CANX,CXCL10,HRH3,NMUR2,PLA2G1B,NMU,SYK,KNG1,IL6,MYO6,LYN,ANXA1,GAL,CDK5,ANXA2,LEP,NPHS1,LRP2,PLA2G5,LCP2,PTPN11,APOA1,REN,SCARB1
homeostasis of number of cells(leukocyte)	2.980094	IL6,IL2RA,LYN,RB1,TGFB1,TIMP1,AKT1,CASP3,ID2,SP1,BAX,BCL2,VEGFA,SH2B2,F2R,MYC
regulation of organic acid transport	2.959865	AKT1,IRS2,TNF,HRH3,PLA2G10,TRH,THBS1
regulation of phospholipase A2 activity	2.814571	EGFR, AGTR1, NMUR2, PLA2G1B EGFR, AGTR1, NMUR2, PLA2G1B EGFR, NMUR2, PLA2G1B
renal system process(water	2.751401	KNG1,AGTR1,AGTR2,ADORA2A,DRD2,TACR1,BCL2,EDN1,ADORA1,IGF1,TACR2,NPHS1,ANXA2

homeostasis)		
regulation of endothelial cell proliferation	2.746807	CAV1,HIF1A,CCL2,PDGFB,APOE,VEGFA,MTOR,THBS1,ARNT
negative regulation of protein kinase cascade(MPKKK and JNK)	2.721119	AMBP,AKT1,CAV1,HDAC3,DRD3,DRD2,SOCS1,NCOR1,ADIPOQ
embryonic development	2.708074	GNA13,AR,CEBPB,EDN1,TP53,BCL2L1,MED21,ITGB1,TPM1,ARNT,EDNRA,APOB,HIF1A,PLCG1,GRIN2B,SP1,NCOA6,PDGFRA,PDGFRB,SMARCA4,HSPG2,VASP,EP300,COL1A1
glucose transport	2.702817	EDNRA,AKT1,PPBP,INS-IGF2,EDN1,PLA2G1B,YES1,SCARB1
regulation of response to food	2.677256	LEP,PPARA,CCK,NPY,MTOR
regulation of protein kinase activity	2.672091	PRKCA,CAV1,INS-IGF2,ADORA2A,PPARG,RB1,AKT1,CASP3,CDKN1B,APOE,RGS4,IL1B,FABP4
regulation of myeloid leukocyte differentiation	2.667903	CALCA,IL5,ID2,JUN,KITLG,RB1,CCL5,ADIPOQ
rhythmic process and female sex differentiation	2.631356	DRD3,ERBB3,DRD2,ERBB2,OXTR,BCL2L1,NPAS2,GRIN2B,BCL2,ANGPT1,NMS,EGFR,DDC,GNRH1,GRIN2A,ARNTL,CDK4,SIRT1,KDR,LEP,PLA2G4A,BAX,JUN,VEGFA,CLOCK,MTNR1A,PTGS2,RPS6KB1,KIT,AKT1,PRKACG,VDR,IGF1R,PTK2B,PAFAH1B1,INSR,AR,SDC1,CCND1,PDGFRA,EPOR,MTOR,PTH,RAC1,PDGFRB,F2R,CTNNB1
response to carbohydrate stimulus	2.596145	CASP6,APOB,PTGS2,LYN,PTK2B,RPS6KB1,TRH,THBS1,ADIPOQ,TGFB1
regulation of carbohydrate biosynthetic process	2.58226	LEP,AKT1,IRS2,INS-IGF2,MTOR,INSR,PPARGC1A,IRS1,ADIPOQ,TNF,IL6ST,NPY1R,MAPK14,PKLR,PIK3CA,GAPDH,MYC
regulation of immune effector process(adaptive immune response)	2.545232	STAT6,IL4,IL6,IL5,IL2RG,INPP5D,TCF3,TGFB1,IL2,SYK,TRAF2,CD86,C3,IL6ST,IL4R,FCER1G,IL1B,B2M,A2M,TNF,INS-IGF2,APOA1,NOS2,VEGFC,ADORA2A,PLA2G1B
posttranscriptional regulation of gene expression	2.539617	MAPK1,IL6,TNF,PTK2B,MTOR,CDK4,THBS1,PRKCA,SMAD3,GIPC1,MAPKAPK2,PPARGC1A,GDNF,PRKCD,FLNA,AKT1,APP,APOA1,BCL2,VEGFA
negative regulation of cAMP biosynthetic process	2.510679	EDNRA, HTR1B, DRD4, EDN1
regulation of cell-cell adhesion	2.510293	TNF,MAP2K1,ITGA6,IL1B,JAK2,ADIPOQ,TGFB1,INSR
muscle(skeletal muscle) tissue development	2.501259	MYOD1,CAV1,ERBB3,RXRA,ERBB2,HSPG2,TPM1,ITGB1,CDK5,APP,EP300,MAPK14,PDGFRB,F2R,MET,LMNA,IGF1,SIRT1,CXCL10
cellular response to extracellular stimulus(nutrient and starvation)	2.492058	SREBF1,VDR,FOS,CAV1,LYN,ITGA6,ALB,JUN,AVPR1A,TP53,SIRT1,PPARGC1A
behavioral interaction between organisms	2.484316	APP,GRIN2B,DRD3,TACR1,DRD4,AVPR1A,GRPR,OXTR,IL1B,MTNR1A,TAC1
positive regulation of blood pressure	2.484031	TACR1,AVPR1B,AVPR1A,ADRA1B,OXTR,ADORA1,TPM1,ADIPOQ,ADORA2A,TAC1,AGTR1,APP,MTNR1A
morphogenesis of a branching structure(tube, blood vessels)	2.480284	EGFR,AKT1,CCND1,CCND3,ADAM17,CASP3,CDKN1B,CDC25C
tube development	2.47538	GNA13,DRD2,EDN1,SMAD4,IGF1,TGFB1,KDR,CTNNB1,EDNRA,CD44,BCL2,VEGFA,EGF,RET,GDNF,VASP,EP300,SP1,CTGF,PDGFRA,FGF1,CRKL,EFNB1,FRS2
positive regulation of immune response	2.456838	MAPK1,LYN,BCAR1,CD79A,SYK,TRAF2,C3,IL6ST,RELA,TLR4,B2M,FYN,PLA2G1B,IL1B,FCER1G,SH2B2
protein import into nucleus	2.289983	AKT1,MAPK1,TNF,JUN,F2,TP53,JAK2,ARNTL,PRL,KPNB1,TGFB1,F2R,CBLB,YWHAZ,TSPO,MYO6,GIPC1,YWHAE,PRKCZ,BCL2L1,ABCA1,TPM1,APP,APOE,BCL2,PAFAH1B1,SAR1B,MAP2K1,CDK5,WAS,FLNA,TOM1L1,ARRB1,BAX,SEC13,LRP2,SH3GL2,DNM2,CTNNB1,EGFR,CAV1,DRD2,PPARG,CANX,CXCL10,APOB,LYN,ACTN4,HSPG2,GRIN2A,CD36,FGFR1OP,RAB5A,COL1A1,LCP2
central nervous system neuron development	2.257234	PRKCA,LEP,PTK2,DRD2,PAFAH1B1,CDK5,EPHB1
negative regulation of homeostatic process	2.23448	CALCA,IAPP,BCL2,VEGFA,INPP5D,TGFB1,BAX
substrate junction	2.129276	ITGA6,PTK2B,BCL2,ACTN1,ITGB3,FN1,SMAD3,TGFB1,VCL

assembly		
positive regulation of lymphocyte differentiation	2.101457	CD83,CD86,IL2RA,IL4R,IL2RG,INPP5D,IL2,SYK,CBLB,ADORA2A,ERBB2
response to axon injury(axon regeneration)	2.099392	MAP2K1,LYN,ERBB2,BCL2,BAX,RXRA,JAK2
regulation of cytokine biosynthetic process(JNK, NFkB)	2.050941	IL6,TNF,KRAS,RELA,PLA2G1B,IL1B,TLR4,TGFB1,CD86,CEBPB,ELANE,INPP5D,SYK,IL6R,LYN
development and migration of cerebral cortex and hippocampus	1.990627	RAC1,LRP8,PAFAH1B1,CDK5,DRD2,BAX,AVPR1A,OXTR,YWHAE
regulation of hydrolase(caspase and peptidase) activity	1.987702	PRKCZ,GPX1,APOA1,ADORA2A,SNCA,APOC3,TP53,NR4A1,SIRT1
regulation of receptor activity	1.953048	APP,ADAM17,SHC1,EGF,PRKCD,SOS1
biogenic amine metabolic process	1.947883	DDC,DRD3,DRD2,DRD4,SNCA,GRIN2A,CHPT1,APOA4,PLA2G4A,APOA1,PLA2G1B,PAFAH1B1,PLA2G5,GALR2,PLA2G2A,PLA2G6,PIK3CA,PLCB1,PIK3R1,LPL
regulation of cell-matrix adhesion	1.906248	PRKCZ, CD36, BCL2, SMAD3, THBS1, RASA1, PIK3R1 PRKCZ, CD36, BCL2, SMAD3, COL1A1, THBS1, RASA1, PIK3R1 COL1A1, THBS1, RASA1, PIK3R1 PRKCZ, CD36, SMAD3 THBS1, RASA1, PIK3R1 PRKCZ, CD36, SMAD3, THBS1
regulation of collagen biosynthetic process	1.889591	IL6,F2,IL6R,TGFB1,F2R,GNA13
sensory organ development	1.818725	EGFR,MYO6,ERBB3,ERBB2,EDN1,MITF,FASLG,STAT3,EPHB1,TGFB1,CTNNB1,CASP6,CDKN1B,SP1,GRIN2B,BAX,BCL2,VEGFA,PDGFRA,FRS2,MYC,KLF4
fatty acid metabolic process	1.800466	TNFRSF1A,ALOX15,PLA2G4A,PTGS2,PLA2G10,EDN1,PLA2G1B,ALOX12B,PLA2G5,SYK,LPL,PPARA,SNCA,PPARGC1A,ADIPOQ,LEP,MAPK14,FABP4,GOT2,GOT1,CHPT1,APOA1,PLA2G6,SCARB1
Rho protein signal transduction	1.73681	GNA13,APOA1,APOE,PTH,APOC3,COL1A2,ADRA2A,ABCA1,ADORA2A
regulation of intracellular transport	1.663856	TNF,SMAD4,SMAD3,IL1B,JAK2,FLNA,TGFB1,SIRT1,ADAM17,GIPC1,THBS1,PTK2,APOE,EFNA1
tube morphogenesis	1.623933	GNA13,RET,EDN1,SMAD4,IGF1,VASP,KDR,CTNNB1,EDNRA,CD44,BCL2,VEGFA,EGF,COL18A1,MAP2K1,PPARG,ANXA1,GPX1,VEGFC,CASP3,S1PR1,JUN,SMARCA4
calcium ion transport into cytosol	1.607892	DRD2,LCK,F2,F2R
desensitization of G-protein coupled receptor protein signaling pathway	1.60272	PLD2,DRD3,DRD2,ADRBK1
regulation of dephosphorylation	1.58897	AGTR2,SMAD3,JAK2,ADORA1,YWHAE,TGFB1
stem cell differentiation	1.548508	CREBBP,PLA2G2A,IGF1,KIT,KLF4
ion transport	1.534188	CAV1,CYSLTR1,DRD2,CAMK2G,GRIN2A,VDR,GRIN2B,NPY,NMUR1,FYN,NMUR2,LCK,F2,CAMK2D,CAMK2B,F2R,NFATC1,TF,LTF,CDKN1B,PDZK1,TSPO,P2RY6
regulation of synapse structure and activity	1.528191	PRKCA,APP,PTK2,UBC,OXTR
regulation of cytoskeleton organization	1.478682	CAV1,CDKN1B,TACR1,EDN1,RAC1,SMAD3,TAC1,MTOR,TPM1,CTNNB1,S1PR1,GSN,MAP2,RASA1,SUMO1,CCK,PRKCZ
erythrocyte differentiation	1.46164	ID2,SP1,LYN,VEGFA,RB1,TIMP1
neuromuscular process	1.44166	APP,GRIN2B,DRD3,ADORA2A,RAC3,DRD2,GRIN2A,PAFAH1B1,SSTR1
myeloid leukocyte activation	1.428903	CSF2,YWHAZ,IL8,LYN,SNCA,CXCR2,TLR4,TGFB1,LCP2,TP53
viral reproductive process	1.37156	CTBP1,CCL2,CCR5,OPRK1,CXCR6,SMAD3,INSR,TGFB1
response to host defenses	1.311431	SMAD3,TLR4,TGFB1

Table S7. Common module enriched GO pathway clusters and detail genes in each cluster – category of *Molecular Function*.

Enriched functional cluster	Enrichment score	Gene
Peptide receptor activity	24.125991	F2RL2,OPRM1,MCHR1,CCKAR,TACR2,TACR1,F2RL1,CXCR1,CXCR2,EDNRA,AGTR1,EDNRB,AGTR2,CXCR5,NMUR1,GALR3,NMUR2,GALR2,CXCR6,NPSR1,RXFP4,C5AR1,CCKBR,NPBWR1,NPBWR2,NTSR2,SSTR4,CCR8,SSTR5,SSTR2,CCR7,CCR6,SSTR3,CCR5,SSTR1,CCR3,GPR17,NPY2R,OPRK1,FPR1,OXTR,BDKRB1,FPR3,BDKRB2,FPR2,NPFFR2,GRPR,OPRL1,NPY1R,AVPR1B,AVPR1A,F2R,OPRD1,RPS6KB1,GPX1,KPNB1,INSR,IGF1R,PIK3R1,BRE,TSPO,GRIN2B,GRIN2A,CHRM4,CHRM3,CHRM2
amine(serotonin) receptor activity	8.47812729	DRD3,DRD2,DRD4,HTR1B,HTR1A,CHRM4,CHRM3,HRH3,CHRM2,HRH4,ADRA1B,ADRA2A,ADRA2C,ADRA2B,HTR1D,HTR2B,HTR2C,HTR5A,HTR1F,HTR1E,HTR2A,GRIN2A,TAT,YWHAE,APOA4,GRIN2B,NOS2
phospholipase activity	8.2433821	F2RL2,LPL,PLD2,CASR,CCKBR,PLA2G10,BDKRB2,EDNRA,PLCB3,PLA2G4A,PLCB4,PLCG1,CHRM3,CCR5,PLA2G2A,PLA2G1B,PLA2G6,PLCB1,PLA2G3,PLCB2,PLA2G2D,PLA2G5
protein dimerization activity	6.45485979	PDGFB,IL6ST,PPARG,NFKB1,ADORA1,GDNF,TGFB1,APOA4,AGTR1,FOS,MAX,APOB,GRIN2B,APOE,NOS2,EGFR,AR,ACTN4,TP53,POLR1C,IL6R,FLNA,AMBP,HIF1A,JUN,VEGFA,PDGFRA,ADORA2A,ERBB3,ERBB2,TRHR,POLA1,APOC2,GIPC1,BCL2L1,BDKRB2,ITGB1,POLR2C,ITGAM,ARNT,BCL2,PAFAH1B1,FIGF,TCF3,CEBPB,CEBPD,CREB1,SMAD4,SMAD3,NR4A1,HGF,ADIPOQ,STAT3,SP1,BAX,SNCA,AKT1,APP,APOA1,RELA,WAS,MED18,COL1A2,COL1A1,SH3GL2,SMARCA4,ITGA2B,CAV1,TNF,KITLG,ITGB3,IGF1R,HNRNPC,THBS1,ITGA4,SIRT1,HDAC1,FYN,
chemokine binding	5.56948457	A2M,IL6ST,CXCR1,CXCR2,AGTR1,TNFRSF1A,CXCR5,IL4R,CXCR6,IL2RG,THBS1,IL2RB,IL2RA,ELANE,IL6R,CCR8,CCR7,CD36,CCR6,CCR5,CCR3,GFRA1,EPOR,IL5RA,GPR17,CD86
Purinergic receptors	5.47145881	P2RY6,ADORA3,GPR18,ADORA2A,P2RY2,P2RY14,P2RY1,LPAR4,GPR17,ADORA1
polysaccharide binding	4.59194579	FGFR2,KNG1,SELP,FGFR1,LPL,CCL2,ITGAM,APOB,APP,CTGF,APOE,VEGFA,SERPINC1,PAFAH1B1,FGF1,THBS1,PLA2G5,FN1,CD44,CD14,OLR1,CANX,IL2
lipoprotein binding(High)	4.31321908	APOB,CD36,APOA1,LDLR,APOE,APOC3,APOC2,SCARB1,ADIPOQ,LRP1,OLR1,LRP8,THBS1
chemokine activity	3.9893998	CCL2,PPBP,IL8,CXCL5,CCL21,CXCL13,CXCL9,CCL19,CCL5,CXCL10,IL4,CSF2,IL3,IL6,TNF,IL5,FASLG,ADIPOQ,VEGFA,IL1B,IL2
promoter binding	3.31741612	SREBF1,TBL1XR1,RXRA,TP53,ESR1,SMAD4,SMAD3,NFKB1,CTNNB1,FOS,VDR,SP1,JUN,TCF3,EGFR,CREB1,MYC,KLF4
lipoprotein binding(low)	2.8942473	CD36,LRP1,ITGAV,SCARB1,THBS1,APOA1,LDLR,OLR1,APOE,LRP8
adrenoceptor activity	2.48574591	ADRA2A, ADRA1B, ADRA2C, ADRA2B
protein binding, bridging(molecular adaptor)	2.41086357	KHDRBS1,CRKL,VAV3,GRB2,SH2B2,SHC1,CRK,FRS2,SRC,BLNK,COL1A2,ANXA1
lipopolysaccharide binding	2.2016448	SELP,SCARB1,TLR4,PTAFR,CD14
sphingolipid binding	2.09431511	SELP, S1PR1, LYN, IL2
prostanoid receptor activity	1.90733028	PTGER1,PPARG,TBXA2R,PTGFR
carboxylic acid binding	1.41651404	SNCA,FFAR1,PPARG,GRIN2A,YWHAE,TAT,GOT1,GRIN2B,ALB,PLA2G1B,FABP4,NOS2,INSR

Table S8. GO pathway clusters enriched by genes of stress-related disease/system common module.

**Annotation Cluster 1**	**Enrichment Score: 41.147572839534696**										
Category	Term	Count	%	PValue	List Total	Pop Hits	Pop Total	Fold Enrichment	Bonferroni	Benjamini	FDR
GOTERM_BP_FAT	GO:0048878~chemical homeostasis	114	20.57762	4.43E-53	549	512	13528	5.48651	1.59E-49	1.77E-50	8.14E-50
GOTERM_BP_FAT	GO:0042592~homeostatic process	132	23.82671	2.13E-49	549	751	13528	4.33107	7.62E-46	5.45E-47	3.90E-46
GOTERM_BP_FAT	GO:0055074~calcium ion homeostasis	67	12.09386	1.43E-44	549	188	13528	8.781692	5.12E-41	3.01E-42	2.62E-41
GOTERM_BP_FAT	GO:0006874~cellular calcium ion homeostasis	66	11.91336	2.86E-44	549	183	13528	8.886978	1.02E-40	5.39E-42	5.25E-41
GOTERM_BP_FAT	GO:0006875~cellular metal ion homeostasis	67	12.09386	3.06E-43	549	196	13528	8.423256	1.10E-39	5.23E-41	5.62E-40
GOTERM_BP_FAT	GO:0055065~metal ion homeostasis	68	12.27437	6.16E-43	549	205	13528	8.173655	2.21E-39	1.00E-40	1.13E-39
GOTERM_BP_FAT	GO:0030005~cellular di-, tri-valent inorganic cation homeostasis	70	12.63538	7.27E-42	549	227	13528	7.598597	2.61E-38	1.13E-39	1.34E-38
GOTERM_BP_FAT	GO:0055066~di-, tri-valent inorganic cation homeostasis	71	12.81588	2.85E-41	549	239	13528	7.320179	1.02E-37	4.09E-39	5.23E-38
GOTERM_BP_FAT	GO:0055082~cellular chemical homeostasis	87	15.70397	5.45E-41	549	380	13528	5.64153	1.95E-37	6.98E-39	1.00E-37
GOTERM_BP_FAT	GO:0006873~cellular ion homeostasis	86	15.52347	1.17E-40	549	374	13528	5.66615	4.19E-37	1.44E-38	2.14E-37
GOTERM_BP_FAT	GO:0030003~cellular cation homeostasis	72	12.99639	2.36E-40	549	254	13528	6.984897	8.48E-37	2.74E-38	4.34E-37
GOTERM_BP_FAT	GO:0051480~cytosolic calcium ion homeostasis	51	9.205776	1.24E-38	549	118	13528	10.65	4.45E-35	1.24E-36	2.28E-35
GOTERM_BP_FAT	GO:0050801~ion homeostasis	87	15.70397	2.40E-38	549	409	13528	5.241519	8.59E-35	2.32E-36	4.40E-35
GOTERM_BP_FAT	GO:0055080~cation homeostasis	73	13.1769	1.41E-37	549	286	13528	6.289528	5.07E-34	1.21E-35	2.59E-34
GOTERM_BP_FAT	GO:0007204~elevation of cytosolic calcium ion concentration	48	8.66426	1.44E-36	549	110	13528	10.75251	5.18E-33	1.21E-34	2.65E-33
GOTERM_BP_FAT	GO:0019725~cellular homeostasis	90	16.24549	3.18E-36	549	466	13528	4.759023	1.14E-32	2.60E-34	5.85E-33
**Annotation Cluster 2**	**Enrichment Score: 37.583771569797285**										
Category	Term	Count	%	PValue	List Total	Pop Hits	Pop Total	Fold Enrichment	Bonferroni	Benjamini	FDR
GOTERM_BP_FAT	GO:0040012~regulation of locomotion	67	12.09386	6.75E-44	549	192	13528	8.59874	2.42E-40	1.21E-41	1.24E-40
GOTERM_BP_FAT	GO:0051270~regulation of cell motion	64	11.55235	2.37E-40	549	193	13528	8.171164	8.50E-37	2.66E-38	4.35E-37
GOTERM_BP_FAT	GO:0030334~regulation of cell migration	60	10.83032	1.02E-39	549	169	13528	8.748343	3.64E-36	1.10E-37	1.87E-36
GOTERM_BP_FAT	GO:0040017~positive regulation of locomotion	45	8.122744	1.92E-35	549	98	13528	11.31482	6.89E-32	1.53E-33	3.53E-32
GOTERM_BP_FAT	GO:0051272~positive regulation of cell motion	45	8.122744	1.92E-35	549	98	13528	11.31482	6.89E-32	1.53E-33	3.53E-32
GOTERM_BP_FAT	GO:0030335~positive regulation of cell migration	43	7.761733	5.25E-35	549	89	13528	11.90528	1.88E-31	4.00E-33	9.63E-32

**Annotation Cluster 3**	**Enrichment Score: 37.22787657505418**										
Category	Term	Count	%	PValue	List Total	Pop Hits	Pop Total	Fold Enrichment	Bonferroni	Benjamini	FDR
GOTERM_BP_FAT	GO:0044093~positive regulation of molecular function	133	24.00722	1.08E-63	549	586	13528	5.59262	3.87E-60	1.29E-60	1.98E-60
GOTERM_BP_FAT	GO:0043085~positive regulation of catalytic activity	124	22.38267	1.10E-61	549	520	13528	5.87597	3.94E-58	7.89E-59	2.02E-58
GOTERM_BP_FAT	GO:0019220~regulation of phosphate metabolic process	110	19.8556	5.84E-52	549	485	13528	5.588718	2.10E-48	2.10E-49	1.07E-48
GOTERM_BP_FAT	GO:0051174~regulation of phosphorus metabolic process	110	19.8556	5.84E-52	549	485	13528	5.588718	2.10E-48	2.10E-49	1.07E-48
GOTERM_BP_FAT	GO:0042325~regulation of phosphorylation	107	19.31408	5.10E-51	549	466	13528	5.65795	1.83E-47	1.41E-48	9.37E-48
GOTERM_BP_FAT	GO:0033674~positive regulation of kinase activity	70	12.63538	2.63E-41	549	231	13528	7.46702	9.42E-38	3.92E-39	4.82E-38
GOTERM_BP_FAT	GO:0051347~positive regulation of transferase activity	71	12.81588	3.88E-41	549	240	13528	7.289678	1.39E-37	5.35E-39	7.12E-38
GOTERM_BP_FAT	GO:0045860~positive regulation of protein kinase activity	66	11.91336	3.40E-38	549	223	13528	7.292901	1.22E-34	3.21E-36	6.25E-35
GOTERM_BP_FAT	GO:0051338~regulation of transferase activity	83	14.98195	3.74E-38	549	372	13528	5.497895	1.34E-34	3.44E-36	6.86E-35
GOTERM_BP_FAT	GO:0043549~regulation of kinase activity	81	14.62094	9.05E-38	549	357	13528	5.590853	3.25E-34	7.92E-36	1.66E-34
GOTERM_BP_FAT	GO:0045859~regulation of protein kinase activity	77	13.89892	2.49E-35	549	345	13528	5.499623	8.92E-32	1.94E-33	4.57E-32
GOTERM_BP_FAT	GO:0043406~positive regulation of MAP kinase activity	36	6.498195	1.20E-23	549	102	13528	8.696882	4.29E-20	4.47E-22	2.20E-20
GOTERM_BP_FAT	GO:0043405~regulation of MAP kinase activity	40	7.220217	2.47E-22	549	141	13528	6.990402	8.85E-19	8.35E-21	4.53E-19
GOTERM_BP_FAT	GO:0000165~MAPKKK cascade	45	8.122744	2.87E-22	549	184	13528	6.026372	1.03E-18	9.62E-21	5.27E-19
GOTERM_BP_FAT	GO:0032147~activation of protein kinase activity	28	5.054152	5.53E-14	549	114	13528	6.052216	1.98E-10	1.14E-12	1.02E-10
GOTERM_BP_FAT	GO:0000187~activation of MAPK activity	23	4.151625	8.03E-13	549	82	13528	6.911546	2.88E-09	1.52E-11	1.48E-09

Annotation Cluster 4	Enrichment Score: 33.60882322472335										
Category	Term	Count	%	PValue	List Total	Pop Hits	Pop Total	Fold Enrichment	Bonferroni	Benjamini	FDR
GOTERM_BP_FAT	GO:0009611~response to wounding	106	19.13357	1.80E-44	549	530	13528	4.928233	6.44E-41	3.58E-42	3.30E-41
GOTERM_BP_FAT	GO:0006954~inflammatory response	70	12.63538	4.91E-31	549	325	13528	5.307328	1.76E-27	2.71E-29	9.01E-28
GOTERM_BP_FAT	GO:0006952~defense response	91	16.42599	1.69E-27	549	615	13528	3.646091	6.07E-24	7.31E-26	3.11E-24

Annotation Cluster 5	Enrichment Score: 31.743600757203737										
Category	Term	Count	%	PValue	List Total	Pop Hits	Pop Total	Fold Enrichment	Bonferroni	Benjamini	FDR
GOTERM_BP_FAT	GO:0010033~response to organic substance	139	25.09025	2.20E-57	549	721	13528	4.750516	7.88E-54	9.85E-55	4.04E-54
GOTERM_BP_FAT	GO:0009719~response to endogenous stimulus	101	18.23105	1.42E-51	549	405	13528	6.145081	5.09E-48	4.24E-49	2.61E-48
GOTERM_BP_FAT	GO:0009725~response to hormone stimulus	93	16.787	5.91E-48	549	367	13528	6.244219	2.12E-44	1.41E-45	1.08E-44

GOTERM_BP_FAT	GO:0032870~cellular response to hormone stimulus	38	6.859206	2.41E-21	549	133	13528	7.040333	8.65E-18	7.59E-20	4.43E-18
GOTERM_BP_FAT	GO:0043434~response to peptide hormone stimulus	39	7.039711	6.76E-20	549	154	13528	6.240295	2.42E-16	1.86E-18	1.24E-16
GOTERM_BP_FAT	GO:0032868~response to insulin stimulus	30	5.415162	1.53E-17	549	100	13528	7.39235	5.51E-14	3.72E-16	2.82E-14
GOTERM_BP_FAT	GO:0032869~cellular response to insulin stimulus	20	3.610108	1.35E-11	549	68	13528	7.247402	4.85E-08	2.30E-10	2.48E-08

Annotation Cluster 6	Enrichment Score: 30.073418311859644										
Category	Term	Count	%	PValue	List Total	Pop Hits	Pop Total	Fold Enrichment	Bonferroni	Benjamini	FDR
GOTERM_BP_FAT	GO:0006928~cell motion	94	16.96751	8.25E-39	549	475	13528	4.876357	2.96E-35	8.46E-37	1.52E-35
GOTERM_BP_FAT	GO:0016477~cell migration	63	11.37184	2.19E-29	549	276	13528	5.624614	7.85E-26	1.14E-27	4.02E-26
GOTERM_BP_FAT	GO:0048870~cell motility	64	11.55235	1.68E-27	549	307	13528	5.136921	6.02E-24	7.34E-26	3.08E-24
GOTERM_BP_FAT	GO:0051674~localization of cell	64	11.55235	1.68E-27	549	307	13528	5.136921	6.02E-24	7.34E-26	3.08E-24

Annotation Cluster 7	Enrichment Score: 29.056265701966243										
Category	Term	Count	%	PValue	List Total	Pop Hits	Pop Total	Fold Enrichment	Bonferroni	Benjamini	FDR
GOTERM_BP_FAT	GO:0007610~behavior	108	19.49458	1.18E-51	549	469	13528	5.674298	4.24E-48	3.85E-49	2.17E-48
GOTERM_BP_FAT	GO:0007626~locomotory behavior	58	10.46931	2.90E-25	549	274	13528	5.216013	1.04E-21	1.16E-23	5.33E-22
GOTERM_BP_FAT	GO:0042330~taxis	41	7.400722	4.17E-21	549	160	13528	6.314299	1.50E-17	1.28E-19	7.66E-18
GOTERM_BP_FAT	GO:0006935~chemotaxis	41	7.400722	4.17E-21	549	160	13528	6.314299	1.50E-17	1.28E-19	7.66E-18

Annotation Cluster 8	Enrichment Score: 28.854651362029166										
Category	Term	Count	%	PValue	List Total	Pop Hits	Pop Total	Fold Enrichment	Bonferroni	Benjamini	FDR
GOTERM_BP_FAT	GO:0060191~regulation of lipase activity	42	7.581227	3.64E-34	549	87	13528	11.89574	1.31E-30	2.67E-32	6.69E-31
GOTERM_BP_FAT	GO:0010518~positive regulation of phospholipase activity	38	6.859206	1.25E-33	549	69	13528	13.5705	4.49E-30	8.64E-32	2.30E-30
GOTERM_BP_FAT	GO:0060193~positive regulation of lipase activity	39	7.039711	2.86E-33	549	75	13528	12.81341	1.03E-29	1.94E-31	5.25E-30
GOTERM_BP_FAT	GO:0010517~regulation of phospholipase activity	38	6.859206	5.14E-33	549	71	13528	13.18823	1.84E-29	3.41E-31	9.43E-30
GOTERM_BP_FAT	GO:0051345~positive regulation of hydrolase activity	55	9.927798	1.13E-32	549	179	13528	7.571308	4.05E-29	7.37E-31	2.07E-29
GOTERM_BP_FAT	GO:0010863~positive regulation of phospholipase C activity	34	6.137184	4.52E-29	549	65	13528	12.88923	1.62E-25	2.25E-27	8.30E-26
GOTERM_BP_FAT	GO:0007202~activation of phospholipase C activity	34	6.137184	4.52E-29	549	65	13528	12.88923	1.62E-25	2.25E-27	8.30E-26
GOTERM_BP_FAT	GO:0051336~regulation of hydrolase activity	66	11.91336	9.75E-27	549	337	13528	4.825866	3.50E-23	4.02E-25	1.79E-23
GOTERM_BP_FAT	GO:0048015~phosphoinositide-mediated signaling	33	5.956679	1.27E-22	549	88	13528	9.240437	4.54E-19	4.41E-21	2.33E-19
GOTERM_BP_FAT	GO:0007200~activation of phospholipase C activity by G-protein coupled receptor protein signaling pathway coupled to IP3 second	26	4.693141	1.49E-21	549	52	13528	12.32058	5.33E-18	4.81E-20	2.73E-18

	messenger											
Annotation Cluster 9	Enrichment Score: 27.44655063012572											
Category	Term	Count	%	PValue	List Total	Pop Hits	Pop Total	Fold Enrichment	Bonferroni	Benjamini	FDR
GOTERM_BP_FAT	GO:0019932~second-messenger-mediated signaling	89	16.06498	2.31E-62	549	235	13528	9.332186	8.27E-59	2.07E-59	4.24E-59
GOTERM_BP_FAT	GO:0019935~cyclic-nucleotide-mediated signaling	55	9.927798	4.13E-41	549	130	13528	10.42511	1.48E-37	5.49E-39	7.59E-38
GOTERM_BP_FAT	GO:0007187~G-protein signaling, coupled to cyclic nucleotide second messenger	52	9.386282	1.28E-40	549	115	13528	11.14209	4.60E-37	1.53E-38	2.36E-37
GOTERM_BP_FAT	GO:0030817~regulation of cAMP biosynthetic process	43	7.761733	3.52E-32	549	101	13528	10.49079	1.26E-28	2.14E-30	6.47E-29
GOTERM_BP_FAT	GO:0030814~regulation of cAMP metabolic process	43	7.761733	9.37E-32	549	103	13528	10.28709	3.36E-28	5.60E-30	1.72E-28
GOTERM_BP_FAT	GO:0030802~regulation of cyclic nucleotide biosynthetic process	44	7.942238	1.46E-31	549	110	13528	9.856466	5.22E-28	8.42E-30	2.67E-28
GOTERM_BP_FAT	GO:0030808~regulation of nucleotide biosynthetic process	44	7.942238	1.46E-31	549	110	13528	9.856466	5.22E-28	8.42E-30	2.67E-28
GOTERM_BP_FAT	GO:0030799~regulation of cyclic nucleotide metabolic process	44	7.942238	5.55E-31	549	113	13528	9.59479	1.99E-27	3.02E-29	1.02E-27
GOTERM_BP_FAT	GO:0006140~regulation of nucleotide metabolic process	44	7.942238	2.02E-30	549	116	13528	9.346649	7.23E-27	1.08E-28	3.70E-27
GOTERM_BP_FAT	GO:0045761~regulation of adenylate cyclase activity	40	7.220217	1.74E-29	549	96	13528	10.26715	6.25E-26	9.20E-28	3.20E-26
GOTERM_BP_FAT	GO:0051350~negative regulation of lyase activity	32	5.776173	2.90E-29	549	55	13528	14.33668	1.04E-25	1.46E-27	5.32E-26
GOTERM_BP_FAT	GO:0031280~negative regulation of cyclase activity	32	5.776173	2.90E-29	549	55	13528	14.33668	1.04E-25	1.46E-27	5.32E-26
GOTERM_BP_FAT	GO:0007194~negative regulation of adenylate cyclase activity	32	5.776173	2.90E-29	549	55	13528	14.33668	1.04E-25	1.46E-27	5.32E-26
GOTERM_BP_FAT	GO:0031279~regulation of cyclase activity	40	7.220217	7.12E-29	549	99	13528	9.956027	2.55E-25	3.50E-27	1.31E-25
GOTERM_BP_FAT	GO:0051339~regulation of lyase activity	40	7.220217	1.76E-28	549	101	13528	9.758878	6.31E-25	8.20E-27	3.23E-25
GOTERM_BP_FAT	GO:0007188~G-protein signaling, coupled to cAMP nucleotide second messenger	37	6.6787	4.49E-28	549	85	13528	10.72615	1.61E-24	2.01E-26	8.24E-25
GOTERM_BP_FAT	GO:0019933~cAMP-mediated signaling	38	6.859206	3.09E-27	549	95	13528	9.856466	1.11E-23	1.32E-25	5.68E-24
GOTERM_BP_FAT	GO:0007193~inhibition of adenylate cyclase activity by G-protein signaling	27	4.873646	4.69E-27	549	40	13528	16.63279	1.68E-23	1.95E-25	8.61E-24
GOTERM_BP_FAT	GO:0043086~negative regulation of catalytic activity	56	10.1083	2.27E-23	549	277	13528	4.981608	8.12E-20	8.29E-22	4.16E-20
GOTERM_BP_FAT	GO:0044092~negative regulation of molecular function	61	11.01083	4.66E-23	549	334	13528	4.500333	1.67E-19	1.67E-21	8.56E-20
GOTERM_BP_FAT	GO:0007190~activation of adenylate cyclase activity	17	3.068592	2.91E-10	549	55	13528	7.61636	1.04E-06	4.31E-09	5.34E-07
GOTERM_BP_FAT	GO:0045762~positive regulation of adenylate cyclase activity	17	3.068592	3.92E-10	549	56	13528	7.480354	1.41E-06	5.65E-09	7.20E-07
GOTERM_BP_FAT	GO:0031281~positive regulation of cyclase activity	17	3.068592	5.25E-10	549	57	13528	7.34912	1.88E-06	7.44E-09	9.64E-07
GOTERM_BP_FAT	GO:0051349~positive regulation of lyase activity	17	3.068592	9.22E-10	549	59	13528	7.099997	3.31E-06	1.28E-08	1.69E-06
Annotation Cluster 10	Enrichment Score: 25.365509480702663											

Category	Term	Count	%	PValue	List Total	Pop Hits	Pop Total	Fold Enrichment	Bonferroni	Benjamini	FDR
GOTERM_CC_FAT	GO:0005886~plasma membrane	295	53.2491	1.65E-39	520	3777	12782	1.919864	6.44E-37	6.44E-37	2.30E-36
GOTERM_CC_FAT	GO:0005887~integral to plasma membrane	151	27.25632	3.19E-39	520	1188	12782	3.124323	1.24E-36	6.20E-37	4.43E-36
GOTERM_CC_FAT	GO:0044459~plasma membrane part	212	38.26715	3.66E-38	520	2203	12782	2.365467	1.42E-35	4.74E-36	5.08E-35
GOTERM_CC_FAT	GO:0031226~intrinsic to plasma membrane	151	27.25632	4.86E-38	520	1215	12782	3.054894	1.89E-35	4.73E-36	6.75E-35
GOTERM_CC_FAT	GO:0031224~intrinsic to membrane	214	38.62816	0.82236	520	5485	12782	0.959031	1	0.978999	100
GOTERM_CC_FAT	GO:0016021~integral to membrane	206	37.18412	0.832033	520	5297	12782	0.955945	1	0.981042	100
Annotation Cluster 11	Enrichment Score: 24.12599103968822										
Category	Term	Count	%	PValue	List Total	Pop Hits	Pop Total	Fold Enrichment	Bonferroni	Benjamini	FDR
GOTERM_MF_FAT	GO:0001653~peptide receptor activity	53	9.566787	2.13E-42	523	114	12983	11.54103	1.66E-39	1.66E-39	3.26E-39
GOTERM_MF_FAT	GO:0008528~peptide receptor activity, G-protein coupled	53	9.566787	2.13E-42	523	114	12983	11.54103	1.66E-39	1.66E-39	3.26E-39
GOTERM_MF_FAT	GO:0042277~peptide binding	60	10.83032	7.29E-35	523	203	12983	7.33717	5.68E-32	2.84E-32	1.12E-31
GOTERM_MF_FAT	GO:0008188~neuropeptide receptor activity	19	3.429603	2.80E-15	523	40	12983	11.79144	2.16E-12	4.32E-13	4.24E-12
GOTERM_MF_FAT	GO:0042923~neuropeptide binding	19	3.429603	4.82E-15	523	41	12983	11.50385	3.72E-12	6.20E-13	7.31E-12
GOTERM_MF_FAT	GO:0042165~neurotransmitter binding	25	4.512635	1.74E-12	523	103	12983	6.025265	1.36E-09	1.13E-10	2.66E-09
GOTERM_MF_FAT	GO:0030594~neurotransmitter receptor activity	23	4.151625	1.69E-11	523	95	12983	6.010043	1.32E-08	8.24E-10	2.59E-08
Annotation Cluster 12	Enrichment Score: 22.030470940505204										
Category	Term	Count	%	PValue	List Total	Pop Hits	Pop Total	Fold Enrichment	Bonferroni	Benjamini	FDR
GOTERM_BP_FAT	GO:0010647~positive regulation of cell communication	71	12.81588	1.52E-31	549	329	13528	5.317698	5.47E-28	8.68E-30	2.80E-28
GOTERM_BP_FAT	GO:0009967~positive regulation of signal transduction	59	10.64982	2.26E-24	549	295	13528	4.928233	8.09E-21	8.61E-23	4.14E-21
GOTERM_BP_FAT	GO:0010627~regulation of protein kinase cascade	49	8.844765	5.90E-20	549	249	13528	4.849065	2.11E-16	1.65E-18	1.08E-16
GOTERM_BP_FAT	GO:0010740~positive regulation of protein kinase cascade	35	6.31769	3.72E-15	549	167	13528	5.164316	1.35E-11	8.16E-14	6.93E-12
Annotation Cluster 13	Enrichment Score: 21.585915478806648										
Category	Term	Count	%	PValue	List Total	Pop Hits	Pop Total	Fold Enrichment	Bonferroni	Benjamini	FDR
GOTERM_BP_FAT	GO:0051050~positive regulation of transport	59	10.64982	3.57E-31	549	223	13528	6.519412	1.28E-27	2.00E-29	6.56E-28
GOTERM_BP_FAT	GO:0051046~regulation of secretion	48	8.66426	3.59E-23	549	202	13528	5.855327	1.29E-19	1.30E-21	6.60E-20
GOTERM_BP_FAT	GO:0060341~regulation of cellular localization	47	8.483755	1.78E-18	549	248	13528	4.669898	6.40E-15	4.44E-17	3.28E-15
GOTERM_BP_FAT	GO:0051047~positive regulation of secretion	30	5.415162	1.98E-16	549	109	13528	6.781972	7.96E-13	5.00E-15	4.11E-13

Annotation Cluster 14	Enrichment Score: 21.393105074216873										
Category	Term	Count	%	PValue	List Total	Pop Hits	Pop Total	Fold Enrichment	Bonferroni	Benjamini	FDR
GOTERM_BP_FAT	GO:0048545~response to steroid hormone stimulus	52	9.386282	5.64E-28	549	192	13528	6.673649	2.02E-24	2.50E-26	1.03E-24
GOTERM_BP_FAT	GO:0031960~response to corticosteroid stimulus	31	5.595668	7.50E-21	549	85	13528	8.986778	2.69E-17	2.26E-19	1.38E-17
GOTERM_BP_FAT	GO:0051384~response to glucocorticoid stimulus	27	4.873646	1.57E-17	549	78	13528	8.529634	5.61E-14	3.77E-16	2.87E-14

Annotation Cluster 15	Enrichment Score: 21.332446278883356										
Category	Term	Count	%	PValue	List Total	Pop Hits	Pop Total	Fold Enrichment	Bonferroni	Benjamini	FDR
GOTERM_BP_FAT	GO:0001932~regulation of protein amino acid phosphorylation	51	9.205776	2.48E-29	549	173	13528	7.264159	8.90E-26	1.27E-27	4.56E-26
GOTERM_BP_FAT	GO:0010562~positive regulation of phosphorus metabolic process	37	6.6787	3.98E-25	549	100	13528	9.117231	1.43E-21	1.57E-23	7.31E-22
GOTERM_BP_FAT	GO:0045937~positive regulation of phosphate metabolic process	37	6.6787	3.98E-25	549	100	13528	9.117231	1.43E-21	1.57E-23	7.31E-22
GOTERM_BP_FAT	GO:0042327~positive regulation of phosphorylation	36	6.498195	1.68E-24	549	97	13528	9.145175	6.04E-21	6.57E-23	3.09E-21
GOTERM_BP_FAT	GO:0032268~regulation of cellular protein metabolic process	75	13.53791	2.24E-24	549	474	13528	3.898919	8.03E-21	8.63E-23	4.11E-21
GOTERM_BP_FAT	GO:0031399~regulation of protein modification process	59	10.64982	2.26E-24	549	295	13528	4.928233	8.09E-21	8.61E-23	4.14E-21
GOTERM_BP_FAT	GO:0001934~positive regulation of protein amino acid phosphorylation	33	5.956679	1.90E-22	549	89	13528	9.136612	6.82E-19	6.50E-21	3.49E-19
GOTERM_BP_FAT	GO:0050730~regulation of peptidyl-tyrosine phosphorylation	29	5.234657	1.08E-21	549	68	13528	10.50873	3.88E-18	3.53E-20	1.99E-18
GOTERM_BP_FAT	GO:0051247~positive regulation of protein metabolic process	48	8.66426	1.26E-19	549	243	13528	4.867391	4.54E-16	3.44E-18	2.32E-16
GOTERM_BP_FAT	GO:0032270~positive regulation of cellular protein metabolic process	47	8.483755	1.32E-19	549	233	13528	4.970536	4.75E-16	3.57E-18	2.43E-16
GOTERM_BP_FAT	GO:0031401~positive regulation of protein modification process	38	6.859206	6.03E-16	549	187	13528	5.007296	1.99E-12	1.23E-14	1.02E-12
GOTERM_BP_FAT	GO:0050731~positive regulation of peptidyl-tyrosine phosphorylation	18	3.249097	1.48E-12	549	47	13528	9.437042	5.30E-09	2.74E-11	2.71E-09

Annotation Cluster 16	Enrichment Score: 19.23596083328121										
Category	Term	Count	%	PValue	List Total	Pop Hits	Pop Total	Fold Enrichment	Bonferroni	Benjamini	FDR
GOTERM_BP_FAT	GO:0008015~blood circulation	44	7.942238	3.57E-21	549	186	13528	5.829093	1.28E-17	1.10E-19	6.56E-18
GOTERM_BP_FAT	GO:0003013~circulatory system process	44	7.942238	3.57E-21	549	186	13528	5.829093	1.28E-17	1.10E-19	6.56E-18
GOTERM_BP_FAT	GO:0008217~regulation of blood pressure	30	5.415162	1.53E-17	549	100	13528	7.39235	5.51E-14	3.72E-16	2.82E-14

Annotation Cluster 17	Enrichment Score: 16.814083638410125										
Category	Term	Count	%	PValue	List Total	Pop Hits	Pop Total	Fold Enrichment	Bonferroni	Benjamini	FDR
GOTERM_BP_FAT	GO:0043067~regulation of programmed cell death	114	20.57762	1.14E-32	549	812	13528	3.459474	4.08E-29	7.28E-31	2.09E-29
GOTERM_BP_FAT	GO:0010941~regulation of cell death	114	20.57762	1.60E-32	549	815	13528	3.44674	5.75E-29	1.01E-30	2.94E-29

GOTERM_BP_FAT	GO:0042981~regulation of apoptosis	112	20.21661	9.41E-32	549	804	13528	3.4326	3.38E-28	5.54E-30	1.73E-28
GOTERM_BP_FAT	GO:0043069~negative regulation of programmed cell death	60	10.83032	1.09E-20	549	359	13528	4.118301	3.90E-17	3.22E-19	1.99E-17
GOTERM_BP_FAT	GO:0060548~negative regulation of cell death	60	10.83032	1.25E-20	549	360	13528	4.106861	4.49E-17	3.65E-19	2.30E-17
GOTERM_BP_FAT	GO:0043066~negative regulation of apoptosis	58	10.46931	1.38E-19	549	354	13528	4.037253	4.97E-16	3.71E-18	2.54E-16
GOTERM_BP_FAT	GO:0043065~positive regulation of apoptosis	64	11.55235	2.16E-19	549	430	13528	3.667522	7.74E-16	5.74E-18	3.96E-16
GOTERM_BP_FAT	GO:0043068~positive regulation of programmed cell death	64	11.55235	3.09E-19	549	433	13528	3.642112	1.11E-15	8.16E-18	5.68E-16
GOTERM_BP_FAT	GO:0010942~positive regulation of cell death	64	11.55235	3.92E-19	549	435	13528	3.625367	1.41E-15	1.02E-17	7.21E-16
GOTERM_BP_FAT	GO:0006916~anti-apoptosis	37	6.6787	8.15E-14	549	206	13528	4.42584	2.92E-10	1.65E-12	1.50E-10
GOTERM_BP_FAT	GO:0008219~cell death	68	12.27437	1.10E-10	549	719	13528	2.330458	3.96E-07	1.74E-09	2.03E-07
GOTERM_BP_FAT	GO:0012501~programmed cell death	61	11.01083	1.46E-10	549	611	13528	2.460084	5.23E-07	2.26E-09	2.68E-07
GOTERM_BP_FAT	GO:0016265~death	68	12.27437	1.48E-10	549	724	13528	2.314364	5.29E-07	2.28E-09	2.71E-07
GOTERM_BP_FAT	GO:0006915~apoptosis	59	10.64982	6.38E-10	549	602	13528	2.414998	2.29E-06	9.01E-09	1.17E-06
GOTERM_BP_FAT	GO:0006917~induction of apoptosis	39	7.039711	2.51E-09	549	320	13528	3.003142	9.00E-06	3.33E-08	4.61E-06
GOTERM_BP_FAT	GO:0012502~induction of programmed cell death	39	7.039711	2.74E-09	549	321	13528	2.993786	9.82E-06	3.61E-08	5.03E-06
GOTERM_BP_FAT	GO:0008624~induction of apoptosis by extracellular signals	21	3.790614	2.02E-08	549	112	13528	4.620219	7.25E-05	2.31E-07	3.71E-05
Annotation Cluster 18	Enrichment Score: 16.125046564794545										
Category	Term	Count	%	PValue	List Total	Pop Hits	Pop Total	Fold Enrichment	Bonferroni	Benjamini	FDR
GOTERM_BP_FAT	GO:0009991~response to extracellular stimulus	48	8.66426	1.67E-21	549	220	13528	5.376254	5.98E-18	5.34E-20	3.06E-18
GOTERM_BP_FAT	GO:0031667~response to nutrient levels	44	7.942238	3.80E-20	549	197	13528	5.503611	1.36E-16	1.08E-18	6.99E-17
GOTERM_BP_FAT	GO:0007584~response to nutrient	33	5.956679	7.53E-16	549	140	13528	5.808275	2.79E-12	1.71E-14	1.43E-12
GOTERM_BP_FAT	GO:0033273~response to vitamin	18	3.249097	6.62E-10	549	66	13528	6.720318	2.37E-06	9.31E-09	1.22E-06
Annotation Cluster 19	Enrichment Score: 15.687091432005634										
Category	Term	Count	%	PValue	List Total	Pop Hits	Pop Total	Fold Enrichment	Bonferroni	Benjamini	FDR
GOTERM_BP_FAT	GO:0032101~regulation of response to external stimulus	58	10.46931	3.97E-39	549	159	13528	8.988601	1.42E-35	4.18E-37	7.28E-36
GOTERM_BP_FAT	GO:0032103~positive regulation of response to external stimulus	28	5.054152	2.67E-21	549	64	13528	10.78051	9.58E-18	8.33E-20	4.91E-18
GOTERM_BP_FAT	GO:0050727~regulation of inflammatory response	28	5.054152	5.59E-19	549	76	13528	9.078324	2.01E-15	1.43E-17	1.03E-15
GOTERM_BP_FAT	GO:0048584~positive regulation of response to stimulus	45	8.122744	8.26E-18	549	236	13528	4.698527	2.96E-14	2.02E-16	1.52E-14
GOTERM_BP_FAT	GO:0050729~positive regulation of inflammatory response	15	2.707581	2.18E-12	549	30	13528	12.32058	7.80E-09	3.98E-11	4.00E-09

GOTERM_BP_FAT	GO:0002673~regulation of acute inflammatory response	10	1.805054	5.23E-08	549	21	13528	11.73389	1.88E-04	5.70E-07	9.61E-05
GOTERM_BP_FAT	GO:0031349~positive regulation of defense response	16	2.888087	1.77E-07	549	73	13528	5.400803	6.36E-04	1.83E-06	3.26E-04
GOTERM_BP_FAT	GO:0002675~positive regulation of acute inflammatory response	7	1.263538	3.23E-06	549	12	13528	14.37401	0.011502	2.82E-05	0.005923
Annotation Cluster 20	Enrichment Score: 15.514245138159744										
Category	Term	Count	%	PValue	List Total	Pop Hits	Pop Total	Fold Enrichment	Bonferroni	Benjamini	FDR
GOTERM_BP_FAT	GO:0002237~response to molecule of bacterial origin	28	5.054152	2.12E-17	549	86	13528	8.022705	7.62E-14	5.01E-16	3.90E-14
GOTERM_BP_FAT	GO:0032496~response to lipopolysaccharide	26	4.693141	1.34E-16	549	77	13528	8.320394	3.98E-13	2.55E-15	2.00E-13
GOTERM_BP_FAT	GO:0009617~response to bacterium	37	6.6787	1.01E-14	549	193	13528	4.723954	3.62E-11	2.12E-13	1.86E-11
Annotation Cluster 21	Enrichment Score: 14.967751725048533										
Category	Term	Count	%	PValue	List Total	Pop Hits	Pop Total	Fold Enrichment	Bonferroni	Benjamini	FDR
GOTERM_BP_FAT	GO:0044057~regulation of system process	70	12.63538	1.75E-32	549	309	13528	5.582141	6.29E-29	1.09E-30	3.22E-29
GOTERM_BP_FAT	GO:0031644~regulation of neurological system process	40	7.220217	6.19E-21	549	153	13528	6.442135	2.22E-17	1.88E-19	1.14E-17
GOTERM_BP_FAT	GO:0051969~regulation of transmission of nerve impulse	37	6.6787	8.50E-19	549	147	13528	6.202198	3.05E-15	2.15E-17	1.56E-15
GOTERM_BP_FAT	GO:0050804~regulation of synaptic transmission	35	6.31769	4.02E-18	549	136	13528	6.341476	1.44E-14	9.87E-17	7.38E-15
GOTERM_BP_FAT	GO:0031646~positive regulation of neurological system process	14	2.527076	2.12E-09	549	39	13528	8.845547	7.60E-06	2.82E-08	3.89E-06
GOTERM_BP_FAT	GO:0048167~regulation of synaptic plasticity	17	3.068592	3.40E-09	549	64	13528	6.54531	1.22E-05	4.42E-08	6.25E-06
GOTERM_BP_FAT	GO:0051971~positive regulation of transmission of nerve impulse	13	2.34657	1.27E-08	549	37	13528	8.657707	4.55E-05	1.52E-07	2.33E-05
GOTERM_BP_FAT	GO:0050806~positive regulation of synaptic transmission	12	2.166065	5.34E-08	549	34	13528	8.696882	1.92E-04	5.79E-07	9.81E-05
Annotation Cluster 22	Enrichment Score: 14.464370980299549										
Category	Term	Count	%	PValue	List Total	Pop Hits	Pop Total	Fold Enrichment	Bonferroni	Benjamini	FDR
GOTERM_BP_FAT	GO:0001944~vasculature development	46	8.303249	1.66E-17	549	251	13528	4.515911	5.95E-14	3.97E-16	3.05E-14
GOTERM_BP_FAT	GO:0001568~blood vessel development	45	8.122744	3.58E-17	549	245	13528	4.525928	1.28E-13	8.40E-16	6.58E-14
GOTERM_BP_FAT	GO:0048514~blood vessel morphogenesis	39	7.039711	5.87E-15	549	211	13528	4.554528	2.11E-11	1.25E-13	1.08E-11
GOTERM_BP_FAT	GO:0001525~angiogenesis	28	5.054152	3.98E-11	549	148	13528	4.661842	1.43E-07	6.43E-10	7.31E-08
Annotation Cluster 23	Enrichment Score: 14.236456111024996										
Category	Term	Count	%	PValue	List Total	Pop Hits	Pop Total	Fold Enrichment	Bonferroni	Benjamini	FDR
GOTERM_CC_FAT	GO:0005615~extracellular space	83	14.98195	2.16E-19	520	685	12782	2.9784	8.42E-17	1.68E-17	3.01E-16
GOTERM_CC_FAT	GO:0044421~extracellular region part	91	16.42599	2.69E-14	520	960	12782	2.330052	1.05E-11	9.50E-13	3.73E-11

GOTERM_CC_FAT	GO:0005576~extracellular region	140	25.27076	3.35E-11	520	2010	12782	1.712093	1.30E-08	5.43E-10	4.65E-08
Annotation Cluster 24	Enrichment Score: 14.091150476274414										
Category	Term	Count	%	PValue	List Total	Pop Hits	Pop Total	Fold Enrichment	Bonferroni	Benjamini	FDR
GOTERM_BP_FAT	GO:0007267~cell-cell signaling	91	16.42599	2.63E-28	549	600	13528	3.737243	9.43E-25	1.21E-26	4.83E-25
GOTERM_BP_FAT	GO:0019226~transmission of nerve impulse	46	8.303249	5.27E-12	549	350	13528	3.238553	1.89E-08	9.18E-11	9.68E-09
GOTERM_BP_FAT	GO:0007268~synaptic transmission	41	7.400722	2.19E-11	549	298	13528	3.390228	7.85E-08	3.62E-10	4.02E-08
GOTERM_BP_FAT	GO:0050877~neurological system process	87	15.70397	1.42E-07	549	1210	13528	1.77172	5.11E-04	1.49E-06	2.61E-04
Annotation Cluster 25	Enrichment Score: 13.920576035845926										
Category	Term	Count	%	PValue	List Total	Pop Hits	Pop Total	Fold Enrichment	Bonferroni	Benjamini	FDR
GOTERM_BP_FAT	GO:0009628~response to abiotic stimulus	61	11.01083	7.54E-21	549	368	13528	4.084541	2.71E-17	2.25E-19	1.38E-17
GOTERM_BP_FAT	GO:0009314~response to radiation	35	6.31769	9.26E-13	549	200	13528	4.312204	3.32E-09	1.75E-11	1.70E-09
GOTERM_BP_FAT	GO:0009416~response to light stimulus	26	4.693141	2.48E-10	549	138	13528	4.642538	8.89E-07	3.71E-09	4.55E-07
Annotation Cluster 26	Enrichment Score: 12.612262289956451										
Category	Term	Count	%	PValue	List Total	Pop Hits	Pop Total	Fold Enrichment	Bonferroni	Benjamini	FDR
GOTERM_MF_FAT	GO:0004713~protein tyrosine kinase activity	34	6.137184	1.56E-14	523	166	12983	5.084453	1.21E-11	1.51E-12	2.38E-11
GOTERM_BP_FAT	GO:0018108~peptidyl-tyrosine phosphorylation	19	3.429603	6.43E-14	549	46	13528	10.17787	2.31E-10	1.32E-12	1.18E-10
GOTERM_BP_FAT	GO:0018212~peptidyl-tyrosine modification	19	3.429603	1.54E-13	549	48	13528	9.753795	5.54E-10	3.09E-12	2.84E-10
GOTERM_MF_FAT	GO:0004715~non-membrane spanning protein tyrosine kinase activity	16	2.888087	2.30E-11	523	41	12983	9.68745	1.79E-08	1.05E-09	3.51E-08
Annotation Cluster 27	Enrichment Score: 12.369463840236385										
Category	Term	Count	%	PValue	List Total	Pop Hits	Pop Total	Fold Enrichment	Bonferroni	Benjamini	FDR
GOTERM_CC_FAT	GO:0030141~secretory granule	37	6.6787	9.99E-16	520	180	12782	5.052714	3.89E-13	4.32E-14	1.39E-12
GOTERM_CC_FAT	GO:0031091~platelet alpha granule	21	3.790614	2.08E-14	520	56	12782	9.217788	8.12E-12	8.12E-13	2.90E-11
GOTERM_CC_FAT	GO:0031983~vesicle lumen	19	3.429603	6.60E-14	520	46	12782	10.15293	2.57E-11	2.14E-12	9.18E-11
GOTERM_CC_FAT	GO:0031988~membrane-bounded vesicle	64	11.55235	2.29E-13	520	568	12782	2.769664	8.90E-11	6.36E-12	3.18E-10
GOTERM_CC_FAT	GO:0060205~cytoplasmic membrane-bounded vesicle lumen	18	3.249097	4.25E-13	520	44	12782	10.05577	1.65E-10	1.10E-11	5.90E-10
GOTERM_CC_FAT	GO:0016023~cytoplasmic membrane-bounded vesicle	62	11.19134	5.81E-13	520	550	12782	2.770923	2.26E-10	1.41E-11	8.06E-10
GOTERM_CC_FAT	GO:0031982~vesicle	69	12.45487	1.63E-12	520	670	12782	2.531452	6.32E-10	3.72E-11	2.26E-09
GOTERM_CC_FAT	GO:0031093~platelet alpha granule lumen	17	3.068592	1.75E-12	520	41	12782	10.19203	6.81E-10	3.58E-11	2.43E-09

Category	Term	Count	%	PValue	List Total	Pop Hits	Pop Total	Fold Enrichment	Bonferroni	Benjamini	FDR
GOTERM_CC_FAT	GO:0031410~cytoplasmic vesicle	67	12.09386	2.02E-12	520	642	12782	2.565283	7.87E-10	3.93E-11	2.81E-09
GOTERM_CC_FAT	GO:0044433~cytoplasmic vesicle part	30	5.415162	4.52E-10	520	187	12782	3.943439	1.76E-07	6.77E-09	6.29E-07

Annotation Cluster 28	Enrichment Score: 12.027042123458013										
Category	Term	Count	%	PValue	List Total	Pop Hits	Pop Total	Fold Enrichment	Bonferroni	Benjamini	FDR
GOTERM_BP_FAT	GO:0010604~positive regulation of macromolecule metabolic process	125	22.56318	8.79E-38	549	857	13528	3.594102	3.15E-34	7.88E-36	1.61E-34
GOTERM_BP_FAT	GO:0009891~positive regulation of biosynthetic process	107	19.31408	4.13E-34	549	695	13528	3.793676	1.48E-30	2.96E-32	7.58E-31
GOTERM_BP_FAT	GO:0031328~positive regulation of cellular biosynthetic process	106	19.13357	5.66E-34	549	685	13528	3.813086	2.03E-30	3.98E-32	1.04E-30
GOTERM_BP_FAT	GO:0010557~positive regulation of macromolecule biosynthetic process	96	17.32852	8.64E-29	549	654	13528	3.617052	3.10E-25	4.19E-27	1.59E-25
GOTERM_BP_FAT	GO:0051173~positive regulation of nitrogen compound metabolic process	95	17.14801	1.20E-28	549	644	13528	3.634955	4.31E-25	5.67E-27	2.21E-25
GOTERM_BP_FAT	GO:0045935~positive regulation of nucleobase, nucleoside, nucleotide and nucleic acid metabolic process	89	16.06498	1.03E-25	549	624	13528	3.514525	3.69E-22	4.14E-24	1.89E-22
GOTERM_BP_FAT	GO:0045944~positive regulation of transcription from RNA polymerase II promoter	64	11.55235	8.13E-23	549	371	13528	4.250767	2.92E-19	2.86E-21	1.49E-19
GOTERM_BP_FAT	GO:0010628~positive regulation of gene expression	80	14.44043	4.39E-22	549	581	13528	3.392932	1.58E-18	1.46E-20	8.07E-19
GOTERM_BP_FAT	GO:0051254~positive regulation of RNA metabolic process	70	12.63538	1.19E-20	549	481	13528	3.586032	4.27E-17	3.50E-19	2.18E-17
GOTERM_BP_FAT	GO:0045893~positive regulation of transcription, DNA-dependent	69	12.45487	3.28E-20	549	477	13528	3.564445	1.18E-16	9.41E-19	6.02E-17
GOTERM_BP_FAT	GO:0045941~positive regulation of transcription	74	13.3574	3.32E-19	549	564	13528	3.233061	1.19E-15	8.69E-18	6.10E-16
GOTERM_BP_FAT	GO:0006357~regulation of transcription from RNA polymerase II promoter	80	14.44043	4.16E-16	549	727	13528	2.711545	1.59E-12	9.99E-15	8.10E-13
GOTERM_MF_FAT	GO:0016563~transcription activator activity	43	7.761733	2.32E-08	523	410	12983	2.603502	1.80E-05	5.47E-07	3.54E-05
GOTERM_MF_FAT	GO:0008134~transcription factor binding	47	8.483755	2.71E-07	523	513	12983	2.274332	2.11E-04	5.56E-06	4.15E-04
GOTERM_CC_FAT	GO:0005654~nucleoplasm	68	12.27437	4.07E-07	520	882	12782	1.895116	1.58E-04	4.16E-06	5.65E-04
GOTERM_CC_FAT	GO:0044451~nucleoplasm part	48	8.66426	1.36E-06	520	555	12782	2.125904	5.29E-04	1.20E-05	0.00189
GOTERM_CC_FAT	GO:0043233~organelle lumen	107	19.31408	5.35E-05	520	1820	12782	1.445133	0.020606	4.34E-04	0.074327
GOTERM_CC_FAT	GO:0031974~membrane-enclosed lumen	108	19.49458	7.38E-05	520	1856	12782	1.430346	0.028305	5.74E-04	0.102485
GOTERM_MF_FAT	GO:0003712~transcription cofactor activity	31	5.595668	1.51E-04	523	363	12983	2.119964	0.110837	0.001677	0.230407
GOTERM_MF_FAT	GO:0003713~transcription coactivator activity	21	3.790614	4.00E-04	523	214	12983	2.436009	0.267859	0.00389	0.610343
GOTERM_CC_FAT	GO:0031981~nuclear lumen	85	15.34296	4.44E-04	520	1450	12782	1.440942	0.158515	0.002872	0.614424
GOTERM_BP_FAT	GO:0051252~regulation of RNA metabolic process	99	17.87004	0.001465	549	1813	13528	1.345546	0.994791	0.00839	2.65581
GOTERM_BP_FAT	GO:0006355~regulation of transcription, DNA-dependent	97	17.50903	0.001595	549	1773	13528	1.348107	0.996742	0.009077	2.889364
GOTERM_MF_FAT	GO:0030528~transcription regulator activity	82	14.80144	0.004093	523	1512	12983	1.34628	0.959023	0.030252	6.080454

GOTERM_CC_FAT	GO:0070013~intracellular organelle lumen	92	16.6065	0.010662	520	1779	12782	1.271181	0.984544	0.050787	13.83485
GOTERM_MF_FAT	GO:0043565~sequence-specific DNA binding	37	6.6787	0.012721	523	607	12983	1.513165	0.999953	0.077882	17.78488
GOTERM_MF_FAT	GO:0003700~transcription factor activity	50	9.025271	0.059665	523	975	12983	1.27303	1	0.263055	60.97681
GOTERM_BP_FAT	GO:0045449~regulation of transcription	116	20.93863	0.157598	549	2601	13528	1.098952	1	0.512182	95.71296
GOTERM_BP_FAT	GO:0006350~transcription	79	14.25993	0.81969	549	2101	13528	0.926536	1	0.997921	100
GOTERM_MF_FAT	GO:0003677~DNA binding	72	12.99639	0.997328	523	2331	12983	0.766767	1	1	100

Annotation Cluster 29 Enrichment Score: 11.763145356160061

Category	Term	Count	%	PValue	List Total	Pop Hits	Pop Total	Fold Enrichment	Bonferroni	Benjamini	FDR
GOTERM_BP_FAT	GO:0010035~response to inorganic substance	41	7.400722	5.17E-17	549	205	13528	4.928233	1.85E-13	1.20E-15	9.50E-14
GOTERM_BP_FAT	GO:0006979~response to oxidative stress	32	5.776173	5.18E-13	549	164	13528	4.808032	1.86E-09	1.01E-11	9.51E-10
GOTERM_BP_FAT	GO:0000302~response to reactive oxygen species	21	3.790614	1.01E-11	549	75	13528	6.899526	3.62E-08	1.72E-10	1.85E-08
GOTERM_BP_FAT	GO:0042542~response to hydrogen peroxide	15	2.707581	3.28E-08	549	56	13528	6.600312	1.18E-04	3.66E-07	6.02E-05

Annotation Cluster 30 Enrichment Score: 11.417923000970505

Category	Term	Count	%	PValue	List Total	Pop Hits	Pop Total	Fold Enrichment	Bonferroni	Benjamini	FDR
GOTERM_CC_FAT	GO:0042995~cell projection	82	14.80144	2.24E-18	520	697	12782	2.891855	8.72E-16	1.45E-16	3.11E-15
GOTERM_CC_FAT	GO:0043005~neuron projection	48	8.66426	1.50E-13	520	342	12782	3.449933	5.85E-11	4.50E-12	2.09E-10
GOTERM_CC_FAT	GO:0030424~axon	30	5.415162	7.72E-12	520	159	12782	4.637881	3.00E-09	1.36E-10	1.07E-08
GOTERM_CC_FAT	GO:0043025~cell soma	27	4.873646	3.82E-09	520	168	12782	3.950481	1.49E-06	5.12E-08	5.31E-06
GOTERM_CC_FAT	GO:0044463~cell projection part	30	5.415162	8.19E-08	520	234	12782	3.151381	3.18E-05	9.10E-07	1.14E-04

Annotation Cluster 31 Enrichment Score: 10.939793906980762

Category	Term	Count	%	PValue	List Total	Pop Hits	Pop Total	Fold Enrichment	Bonferroni	Benjamini	FDR
GOTERM_CC_FAT	GO:0000267~cell fraction	102	18.41155	6.72E-16	520	1083	12782	2.315086	2.59E-13	3.24E-14	9.21E-13
GOTERM_CC_FAT	GO:0005626~insoluble fraction	74	13.3574	3.51E-10	520	839	12782	2.16803	1.37E-07	5.47E-09	4.88E-07
GOTERM_CC_FAT	GO:0005624~membrane fraction	69	12.45487	6.42E-09	520	809	12782	2.096506	2.50E-06	8.06E-08	8.92E-06

Annotation Cluster 32 Enrichment Score: 10.529441728782434

Category	Term	Count	%	PValue	List Total	Pop Hits	Pop Total	Fold Enrichment	Bonferroni	Benjamini	FDR
GOTERM_BP_FAT	GO:0050727~regulation of inflammatory response	28	5.054152	5.59E-19	549	76	13528	9.078324	2.01E-15	1.43E-17	1.03E-15
GOTERM_BP_FAT	GO:0032102~negative regulation of response to external stimulus	17	3.068592	5.81E-11	549	50	13528	8.377996	2.08E-07	9.26E-10	1.07E-07

GOTERM_BP_FAT	GO:0031348~negative regulation of defense response	12	2.166065	1.04E-07	549	36	13528	8.213722	3.74E-04	1.11E-06	1.91E-04
GOTERM_BP_FAT	GO:0050728~negative regulation of inflammatory response	11	1.98556	2.25E-07	549	31	13528	8.743639	8.08E-04	2.30E-06	4.14E-04

Annotation Cluster 33	Enrichment Score: 10.06774312489739										
Category	Term	Count	%	PValue	List Total	Pop Hits	Pop Total	Fold Enrichment	Bonferroni	Benjamini	FDR
GOTERM_BP_FAT	GO:0019216~regulation of lipid metabolic process	33	5.956679	5.23E-19	549	112	13528	7.260343	1.88E-15	1.35E-17	9.61E-16
GOTERM_BP_FAT	GO:0045834~positive regulation of lipid metabolic process	21	3.790614	2.01E-16	549	46	13528	11.24923	7.96E-13	5.00E-15	4.11E-13
GOTERM_BP_FAT	GO:0019217~regulation of fatty acid metabolic process	16	2.888087	4.73E-10	549	49	13528	8.046095	1.70E-06	6.75E-09	8.68E-07
GOTERM_BP_FAT	GO:0010565~regulation of cellular ketone metabolic process	17	3.068592	5.25E-10	549	57	13528	7.34912	1.88E-06	7.44E-09	9.64E-07
GOTERM_BP_FAT	GO:0050994~regulation of lipid catabolic process	12	2.166065	1.94E-09	549	26	13528	11.37285	6.95E-06	2.60E-08	3.56E-06
GOTERM_BP_FAT	GO:0043255~regulation of carbohydrate biosynthetic process	9	1.624549	8.79E-07	549	21	13528	10.5605	0.003148	8.30E-06	0.001614
GOTERM_BP_FAT	GO:0046320~regulation of fatty acid oxidation	7	1.263538	7.56E-04	549	28	13528	6.160291	0.933593	0.004563	1.378892

Annotation Cluster 34	Enrichment Score: 9.963883187370028										
Category	Term	Count	%	PValue	List Total	Pop Hits	Pop Total	Fold Enrichment	Bonferroni	Benjamini	FDR
GOTERM_BP_FAT	GO:0007243~protein kinase cascade	79	14.25993	7.74E-35	549	370	13528	5.261222	2.78E-31	5.78E-33	1.42E-31
GOTERM_BP_FAT	GO:0006468~protein amino acid phosphorylation	97	17.50903	8.86E-29	549	667	13528	3.583498	3.18E-25	4.24E-27	1.63E-25
GOTERM_BP_FAT	GO:0016310~phosphorylation	99	17.87004	6.92E-24	549	800	13528	3.049344	2.48E-20	2.61E-22	1.27E-20
GOTERM_BP_FAT	GO:0006793~phosphorus metabolic process	104	18.77256	2.95E-20	549	973	13528	2.633794	1.06E-16	8.53E-19	5.41E-17
GOTERM_BP_FAT	GO:0006796~phosphate metabolic process	104	18.77256	2.95E-20	549	973	13528	2.633794	1.06E-16	8.53E-19	5.41E-17
GOTERM_MF_FAT	GO:0004713~protein tyrosine kinase activity	34	6.137184	1.56E-14	523	166	12983	5.084453	1.21E-11	1.51E-12	2.38E-11
GOTERM_MF_FAT	GO:0004672~protein kinase activity	66	11.91336	2.83E-13	523	606	12983	2.703614	2.20E-10	2.20E-11	4.33E-10
GOTERM_MF_FAT	GO:0004674~protein serine/threonine kinase activity	31	5.595668	0.002393	523	430	12983	1.789644	0.845372	0.019458	3.599134
GOTERM_MF_FAT	GO:0001882~nucleoside binding	85	15.34296	0.007014	523	1612	12983	1.308963	0.995843	0.047363	10.20681
GOTERM_MF_FAT	GO:0005524~ATP binding	78	14.07942	0.009681	523	1477	12983	1.310954	0.999488	0.061695	13.82588
GOTERM_MF_FAT	GO:0032559~adenyl ribonucleotide binding	78	14.07942	0.013245	523	1497	12983	1.29344	0.999969	0.079735	18.44983
GOTERM_MF_FAT	GO:0032555~purine ribonucleotide binding	93	16.787	0.01354	523	1836	12983	1.257429	0.999976	0.080217	18.82164
GOTERM_MF_FAT	GO:0032553~ribonucleotide binding	93	16.787	0.01354	523	1836	12983	1.257429	0.999976	0.080217	18.82164
GOTERM_MF_FAT	GO:0001883~purine nucleoside binding	82	14.80144	0.016043	523	1601	12983	1.27144	0.999997	0.091034	21.91678
GOTERM_MF_FAT	GO:0017076~purine nucleotide binding	95	17.14801	0.021402	523	1918	12983	1.229556	1	0.114961	28.17407

GOTERM_MF_FAT	GO:0030554~adenyl nucleotide binding	80	14.44043	0.021898	523	1577	12983	1.259307	1	0.115141	28.72912
GOTERM_MF_FAT	GO:0000166~nucleotide binding	104	18.77256	0.076088	523	2245	12983	1.14998	1	0.316514	70.19621
Annotation Cluster 35	Enrichment Score: 9.70544346565441										
Category	Term	Count	%	PValue	List Total	Pop Hits	Pop Total	Fold Enrichment	Bonferroni	Benjamini	FDR
GOTERM_BP_FAT	GO:0050730~regulation of peptidyl-tyrosine phosphorylation	29	5.234657	1.08E-21	549	68	13528	10.50873	3.88E-18	3.53E-20	1.99E-18
GOTERM_BP_FAT	GO:0050731~positive regulation of peptidyl-tyrosine phosphorylation	18	3.249097	1.48E-12	549	47	13528	9.437042	5.30E-09	2.74E-11	2.71E-09
GOTERM_BP_FAT	GO:0046425~regulation of JAK-STAT cascade	15	2.707581	4.54E-11	549	36	13528	10.26715	1.63E-07	7.30E-10	8.33E-08
GOTERM_BP_FAT	GO:0046427~positive regulation of JAK-STAT cascade	13	2.34657	9.96E-11	549	26	13528	12.32058	3.57E-07	1.58E-09	1.83E-07
GOTERM_BP_FAT	GO:0042509~regulation of tyrosine phosphorylation of STAT protein	12	2.166065	1.77E-08	549	31	13528	9.538516	6.34E-05	2.05E-07	3.24E-05
GOTERM_BP_FAT	GO:0042531~positive regulation of tyrosine phosphorylation of STAT protein	10	1.805054	1.35E-07	549	23	13528	10.71355	4.85E-04	1.42E-06	2.48E-04
GOTERM_BP_FAT	GO:0042522~regulation of tyrosine phosphorylation of Stat5 protein	7	1.263538	5.78E-06	549	13	13528	13.26832	0.020537	4.85E-05	0.010623
GOTERM_BP_FAT	GO:0042523~positive regulation of tyrosine phosphorylation of Stat5 protein	6	1.083032	2.28E-05	549	10	13528	14.7847	0.078497	1.76E-04	0.041846
Annotation Cluster 36	Enrichment Score: 9.202748419077007										
Category	Term	Count	%	PValue	List Total	Pop Hits	Pop Total	Fold Enrichment	Bonferroni	Benjamini	FDR
GOTERM_BP_FAT	GO:0016044~membrane organization	48	8.66426	7.72E-12	549	381	13528	3.104399	2.77E-08	1.33E-10	1.42E-08
GOTERM_BP_FAT	GO:0010324~membrane invagination	35	6.31769	1.47E-11	549	220	13528	3.920185	5.29E-08	2.48E-10	2.71E-08
GOTERM_BP_FAT	GO:0006897~endocytosis	35	6.31769	1.47E-11	549	220	13528	3.920185	5.29E-08	2.48E-10	2.71E-08
GOTERM_BP_FAT	GO:0006909~phagocytosis	14	2.527076	3.60E-08	549	48	13528	7.187007	1.29E-04	3.99E-07	6.60E-05
GOTERM_BP_FAT	GO:0016192~vesicle-mediated transport	49	8.844765	1.60E-06	549	576	13528	2.09621	0.005738	1.47E-05	0.002946
Annotation Cluster 37	Enrichment Score: 9.200082862143061										
Category	Term	Count	%	PValue	List Total	Pop Hits	Pop Total	Fold Enrichment	Bonferroni	Benjamini	FDR
GOTERM_BP_FAT	GO:0050900~leukocyte migration	19	3.429603	4.67E-12	549	57	13528	8.213722	1.67E-08	8.21E-11	8.57E-09
GOTERM_BP_FAT	GO:0060326~cell chemotaxis	15	2.707581	1.61E-10	549	39	13528	9.477371	5.78E-07	2.48E-09	2.96E-07
GOTERM_BP_FAT	GO:0030595~leukocyte chemotaxis	14	2.527076	1.00E-09	549	37	13528	9.323684	3.59E-06	1.38E-08	1.84E-06
GOTERM_BP_FAT	GO:0030593~neutrophil chemotaxis	9	1.624549	2.11E-07	549	18	13528	12.32058	7.55E-04	2.16E-06	3.87E-04
Annotation Cluster 38	Enrichment Score: 9.016985298186977										
Category	Term	Count	%	PValue	List Total	Pop Hits	Pop Total	Fold Enrichment	Bonferroni	Benjamini	FDR

GOTERM_BP_FAT	GO:0051341~regulation of oxidoreductase activity	17	3.068592	1.08E-12	549	40	13528	10.4725	3.88E-09	2.02E-11	1.98E-09
GOTERM_BP_FAT	GO:0032768~regulation of monooxygenase activity	14	2.527076	2.29E-12	549	25	13528	13.79905	8.20E-09	4.16E-11	4.20E-09
GOTERM_BP_FAT	GO:0051353~positive regulation of oxidoreductase activity	14	2.527076	4.41E-12	549	26	13528	13.26832	1.58E-08	7.79E-11	8.09E-09
GOTERM_BP_FAT	GO:0032770~positive regulation of monooxygenase activity	10	1.805054	1.70E-10	549	13	13528	18.95474	6.11E-07	2.60E-09	3.13E-07
GOTERM_BP_FAT	GO:0051000~positive regulation of nitric-oxide synthase activity	7	1.263538	3.26E-07	549	9	13528	19.16535	0.001167	3.24E-06	5.98E-04
GOTERM_BP_FAT	GO:0045429~positive regulation of nitric oxide biosynthetic process	9	1.624549	8.79E-07	549	21	13528	10.5605	0.003148	8.30E-06	0.001614
GOTERM_BP_FAT	GO:0050999~regulation of nitric-oxide synthase activity	8	1.444043	1.43E-06	549	16	13528	12.32058	0.005127	1.33E-05	0.002632
Annotation Cluster 39	Enrichment Score: 8.821448192770333										
Category	Term	Count	%	PValue	List Total	Pop Hits	Pop Total	Fold Enrichment	Bonferroni	Benjamini	FDR
GOTERM_BP_FAT	GO:0051054~positive regulation of DNA metabolic process	18	3.249097	3.76E-11	549	56	13528	7.920375	1.35E-07	6.10E-10	6.90E-08
GOTERM_BP_FAT	GO:0045740~positive regulation of DNA replication	13	2.34657	2.91E-10	549	28	13528	11.44054	1.04E-06	4.30E-09	5.35E-07
GOTERM_BP_FAT	GO:0006275~regulation of DNA replication	16	2.888087	1.71E-08	549	62	13528	6.359011	6.15E-05	2.00E-07	3.15E-05
GOTERM_BP_FAT	GO:0051052~regulation of DNA metabolic process	21	3.790614	2.76E-08	549	114	13528	4.539162	9.90E-05	3.11E-07	5.07E-05
Annotation Cluster 40	Enrichment Score: 8.723813819932504										
Category	Term	Count	%	PValue	List Total	Pop Hits	Pop Total	Fold Enrichment	Bonferroni	Benjamini	FDR
GOTERM_BP_FAT	GO:0001775~cell activation	46	8.303249	3.77E-15	549	287	13528	3.949455	1.35E-11	8.10E-14	6.93E-12
GOTERM_BP_FAT	GO:0045321~leukocyte activation	38	6.859206	2.53E-12	549	242	13528	3.869274	9.07E-09	4.58E-11	4.65E-09
GOTERM_BP_FAT	GO:0048534~hemopoietic or lymphoid organ development	36	6.498195	3.76E-10	549	260	13528	3.411854	1.35E-06	5.43E-09	6.90E-07
GOTERM_BP_FAT	GO:0030097~hemopoiesis	33	5.956679	1.80E-09	549	236	13528	3.445587	6.47E-06	2.45E-08	3.31E-06
GOTERM_BP_FAT	GO:0002520~immune system development	36	6.498195	1.91E-09	549	276	13528	3.214065	6.86E-06	2.58E-08	3.51E-06
GOTERM_BP_FAT	GO:0046649~lymphocyte activation	29	5.234657	8.28E-09	549	199	13528	3.590924	2.97E-05	1.01E-07	1.52E-05
GOTERM_BP_FAT	GO:0002521~leukocyte differentiation	23	4.151625	1.29E-08	549	131	13528	4.326312	4.62E-05	1.54E-07	2.36E-05
GOTERM_BP_FAT	GO:0030183~B cell differentiation	13	2.34657	3.18E-07	549	48	13528	6.673649	0.001138	3.17E-06	5.83E-04
GOTERM_BP_FAT	GO:0030098~lymphocyte differentiation	18	3.249097	7.51E-07	549	103	13528	4.306223	0.002689	7.20E-06	0.001378
GOTERM_BP_FAT	GO:0042113~B cell activation	15	2.707581	1.84E-06	549	76	13528	4.863388	0.006579	1.68E-05	0.00338
Annotation Cluster 41	Enrichment Score: 8.47812729239092										
Category	Term	Count	%	PValue	List Total	Pop Hits	Pop Total	Fold Enrichment	Bonferroni	Benjamini	FDR
GOTERM_MF_FAT	GO:0008227~amine receptor activity	21	3.790614	1.00E-17	523	41	12983	12.71478	7.82E-15	2.61E-15	1.54E-14

GOTERM_MF_FAT	GO:0051378~serotonin binding	8	1.444043	5.48E-09	523	9	12983	22.06586	4.27E-06	1.64E-07	8.38E-06
GOTERM_MF_FAT	GO:0004993~serotonin receptor activity	9	1.624549	6.98E-09	523	13	12983	17.18591	5.44E-06	1.94E-07	1.07E-05
GOTERM_MF_FAT	GO:0043176~amine binding	20	3.610108	7.02E-08	523	110	12983	4.513471	5.47E-05	1.61E-06	1.07E-04
GOTERM_BP_FAT	GO:0007210~serotonin receptor signaling pathway	3	0.541516	0.015098	549	5	13528	14.7847	1	0.071837	24.37517

Annotation Cluster 42 Enrichment Score: 8.243382096856156

Category	Term	Count	%	PValue	List Total	Pop Hits	Pop Total	Fold Enrichment	Bonferroni	Benjamini	FDR
GOTERM_MF_FAT	GO:0004620~phospholipase activity	22	3.971119	7.91E-12	523	83	12983	6.57988	6.16E-09	4.40E-10	1.21E-08
GOTERM_MF_FAT	GO:0016298~lipase activity	22	3.971119	3.48E-10	523	100	12983	5.4613	2.71E-07	1.43E-08	5.32E-07
GOTERM_MF_FAT	GO:0004435~phosphoinositide phospholipase C activity	12	2.166065	1.07E-09	523	25	12983	11.91556	8.32E-07	3.78E-08	1.63E-06
GOTERM_MF_FAT	GO:0004629~phospholipase C activity	12	2.166065	1.63E-08	523	31	12983	9.609326	1.27E-05	4.09E-07	2.49E-05
GOTERM_MF_FAT	GO:0008081~phosphoric diester hydrolase activity	13	2.34657	1.27E-04	523	84	12983	3.841824	0.094055	0.001473	0.193769

Annotation Cluster 43 Enrichment Score: 8.046254952267889

Category	Term	Count	%	PValue	List Total	Pop Hits	Pop Total	Fold Enrichment	Bonferroni	Benjamini	FDR
GOTERM_BP_FAT	GO:0045761~regulation of adenylate cyclase activity	40	7.220217	1.74E-29	549	96	13528	10.26715	6.25E-26	9.20E-28	3.20E-26
GOTERM_BP_FAT	GO:0031279~regulation of cyclase activity	40	7.220217	7.12E-29	549	99	13528	9.956027	2.55E-25	3.50E-27	1.31E-25
GOTERM_BP_FAT	GO:0051339~regulation of lyase activity	40	7.220217	1.76E-28	549	101	13528	9.758878	6.31E-25	8.20E-27	3.23E-25
GOTERM_BP_FAT	GO:0007188~G-protein signaling, coupled to cAMP nucleotide second messenger	37	6.6787	4.49E-28	549	85	13528	10.72615	1.61E-24	2.01E-26	8.24E-25
GOTERM_BP_FAT	GO:0007190~activation of adenylate cyclase activity	17	3.068592	2.91E-10	549	55	13528	7.61636	1.04E-06	4.31E-09	5.34E-07
GOTERM_BP_FAT	GO:0045762~positive regulation of adenylate cyclase activity	17	3.068592	3.92E-10	549	56	13528	7.480354	1.41E-06	5.65E-09	7.20E-07
GOTERM_BP_FAT	GO:0031281~positive regulation of cyclase activity	17	3.068592	5.25E-10	549	57	13528	7.34912	1.88E-06	7.44E-09	9.64E-07
GOTERM_BP_FAT	GO:0051349~positive regulation of lyase activity	17	3.068592	9.22E-10	549	59	13528	7.099997	3.31E-06	1.28E-08	1.69E-06
GOTERM_BP_FAT	GO:0007189~activation of adenylate cyclase activity by G-protein signaling pathway	14	2.527076	3.02E-09	549	40	13528	8.624408	1.08E-05	3.94E-08	5.55E-06
GOTERM_BP_FAT	GO:0010579~positive regulation of adenylate cyclase activity by G-protein signaling pathway	14	2.527076	3.02E-09	549	40	13528	8.624408	1.08E-05	3.94E-08	5.55E-06
GOTERM_BP_FAT	GO:0010578~regulation of adenylate cyclase activity involved in G-protein signaling	14	2.527076	3.02E-09	549	40	13528	8.624408	1.08E-05	3.94E-08	5.55E-06
GOTERM_BP_FAT	GO:0009755~hormone-mediated signaling	15	2.707581	1.16E-08	549	52	13528	7.108029	4.16E-05	1.40E-07	2.13E-05
GOTERM_BP_FAT	GO:0006171~cAMP biosynthetic process	8	1.444043	4.61E-07	549	14	13528	14.08067	0.001654	4.52E-06	8.48E-04
GOTERM_BP_FAT	GO:0046058~cAMP metabolic process	9	1.624549	8.79E-07	549	21	13528	10.5605	0.003148	8.30E-06	0.001614
GOTERM_BP_FAT	GO:0034199~activation of protein kinase A activity	8	1.444043	2.35E-06	549	17	13528	11.59584	0.008398	2.09E-05	0.004318

GOTERM_MF_FAT	GO:0004016~adenylate cyclase activity	7	1.263538	3.08E-06	523	12	12983	14.48072	0.0024	5.11E-05	0.004718
GOTERM_BP_FAT	GO:0009190~cyclic nucleotide biosynthetic process	9	1.624549	4.05E-06	549	25	13528	8.87082	0.014409	3.49E-05	0.007431
GOTERM_BP_FAT	GO:0009187~cyclic nucleotide metabolic process	10	1.805054	1.57E-05	549	38	13528	6.484517	0.054594	1.23E-04	0.028739
GOTERM_MF_FAT	GO:0016849~phosphorus-oxygen lyase activity	7	1.263538	1.77E-04	523	22	12983	7.898575	0.128508	0.001882	0.269725
GOTERM_MF_FAT	GO:0009975~cyclase activity	7	1.263538	2.31E-04	523	23	12983	7.555158	0.164587	0.002427	0.352488
GOTERM_BP_FAT	GO:0009124~nucleoside monophosphate biosynthetic process	9	1.624549	8.23E-04	549	50	13528	4.43541	0.94786	0.004927	1.500942
GOTERM_BP_FAT	GO:0009123~nucleoside monophosphate metabolic process	10	1.805054	0.001298	549	66	13528	3.73351	0.990523	0.007522	2.357093
GOTERM_BP_FAT	GO:0006164~purine nucleotide biosynthetic process	10	1.805054	0.146801	549	148	13528	1.664944	1	0.489088	94.58327
GOTERM_BP_FAT	GO:0006163~purine nucleotide metabolic process	11	1.98556	0.222807	549	186	13528	1.457273	1	0.645443	99.02376
GOTERM_BP_FAT	GO:0044271~nitrogen compound biosynthetic process	17	3.068592	0.246293	549	325	13528	1.288923	1	0.684579	99.44433
GOTERM_BP_FAT	GO:0009165~nucleotide biosynthetic process	10	1.805054	0.340935	549	186	13528	1.324794	1	0.809135	99.95273
GOTERM_BP_FAT	GO:0034654~nucleobase, nucleoside, nucleotide and nucleic acid biosynthetic process	10	1.805054	0.381308	549	193	13528	1.276744	1	0.847444	99.98519
GOTERM_BP_FAT	GO:0034404~nucleobase, nucleoside and nucleotide biosynthetic process	10	1.805054	0.381308	549	193	13528	1.276744	1	0.847444	99.98519
GOTERM_MF_FAT	GO:0000287~magnesium ion binding	16	2.888087	0.813063	523	452	12983	0.878729	1	0.996501	100

Annotation Cluster 44 Enrichment Score: 7.970605575875905

Category	Term	Count	%	PValue	List Total	Pop Hits	Pop Total	Fold Enrichment	Bonferroni	Benjamini	FDR
GOTERM_BP_FAT	GO:0051248~negative regulation of protein metabolic process	30	5.415162	4.40E-10	549	187	13528	3.953128	1.58E-06	6.31E-09	8.07E-07
GOTERM_BP_FAT	GO:0001933~negative regulation of protein amino acid phosphorylation	13	2.34657	1.86E-09	549	32	13528	10.01047	6.68E-06	2.52E-08	3.42E-06
GOTERM_BP_FAT	GO:0010563~negative regulation of phosphorus metabolic process	15	2.707581	3.68E-09	549	48	13528	7.700364	1.32E-05	4.76E-08	6.75E-06
GOTERM_BP_FAT	GO:0045936~negative regulation of phosphate metabolic process	15	2.707581	3.68E-09	549	48	13528	7.700364	1.32E-05	4.76E-08	6.75E-06
GOTERM_BP_FAT	GO:0042326~negative regulation of phosphorylation	14	2.527076	1.52E-08	549	45	13528	7.66614	5.46E-05	1.79E-07	2.79E-05
GOTERM_BP_FAT	GO:0032269~negative regulation of cellular protein metabolic process	27	4.873646	1.63E-08	549	180	13528	3.696175	5.86E-05	1.91E-07	3.00E-05
GOTERM_BP_FAT	GO:0031400~negative regulation of protein modification process	18	3.249097	5.84E-06	549	119	13528	3.727235	0.020723	4.88E-05	0.010721

Annotation Cluster 45 Enrichment Score: 7.795071709471697

Category	Term	Count	%	PValue	List Total	Pop Hits	Pop Total	Fold Enrichment	Bonferroni	Benjamini	FDR
GOTERM_BP_FAT	GO:0042060~wound healing	37	6.6787	7.22E-15	549	191	13528	4.77342	2.59E-11	1.52E-13	1.33E-11
GOTERM_BP_FAT	GO:0050878~regulation of body fluid levels	22	3.971119	2.32E-07	549	141	13528	3.844721	8.33E-04	2.35E-06	4.26E-04
GOTERM_BP_FAT	GO:0050817~coagulation	18	3.249097	6.51E-07	549	102	13528	4.348441	0.002331	6.27E-06	0.001195

GOTERM_BP_FAT	GO:0007596~blood coagulation	18	3.249097	6.51E-07	549	102	13528	4.348441	0.002331	6.27E-06	0.001195
GOTERM_BP_FAT	GO:0007599~hemostasis	18	3.249097	1.49E-06	549	108	13528	4.106861	0.005334	1.38E-05	0.002738
Annotation Cluster 46	Enrichment Score: 7.688672546873157										
Category	Term	Count	%	PValue	List Total	Pop Hits	Pop Total	Fold Enrichment	Bonferroni	Benjamini	FDR
GOTERM_BP_FAT	GO:0030193~regulation of blood coagulation	15	2.707581	4.54E-11	549	36	13528	10.26715	1.63E-07	7.30E-10	8.33E-08
GOTERM_BP_FAT	GO:0050818~regulation of coagulation	15	2.707581	3.49E-10	549	41	13528	9.015061	1.25E-06	5.09E-09	6.41E-07
GOTERM_BP_FAT	GO:0030195~negative regulation of blood coagulation	9	1.624549	1.97E-06	549	23	13528	9.642195	0.007044	1.78E-05	0.003619
GOTERM_BP_FAT	GO:0050819~negative regulation of coagulation	9	1.624549	5.64E-06	549	26	13528	8.529634	0.020022	4.75E-05	0.010354
Annotation Cluster 47	Enrichment Score: 7.465814604199877										
Category	Term	Count	%	PValue	List Total	Pop Hits	Pop Total	Fold Enrichment	Bonferroni	Benjamini	FDR
GOTERM_BP_FAT	GO:0043279~response to alkaloid	18	3.249097	1.95E-11	549	54	13528	8.213722	7.00E-08	3.24E-10	3.58E-08
GOTERM_BP_FAT	GO:0014072~response to isoquinoline alkaloid	8	1.444043	1.43E-06	549	16	13528	12.32058	0.005127	1.33E-05	0.002632
GOTERM_BP_FAT	GO:0043278~response to morphine	8	1.444043	1.43E-06	549	16	13528	12.32058	0.005127	1.33E-05	0.002632
Annotation Cluster 48	Enrichment Score: 7.331612683359194										
Category	Term	Count	%	PValue	List Total	Pop Hits	Pop Total	Fold Enrichment	Bonferroni	Benjamini	FDR
GOTERM_BP_FAT	GO:0010243~response to organic nitrogen	20	3.610108	2.99E-12	549	63	13528	7.822592	1.07E-08	5.39E-11	5.49E-09
GOTERM_BP_FAT	GO:0014075~response to amine stimulus	14	2.527076	2.12E-09	549	39	13528	8.845547	7.60E-06	2.82E-08	3.89E-06
GOTERM_BP_FAT	GO:0001101~response to acid	11	1.98556	1.29E-08	549	24	13528	11.29387	4.62E-05	1.53E-07	2.37E-05
GOTERM_BP_FAT	GO:0043200~response to amino acid stimulus	7	1.263538	3.63E-05	549	17	13528	10.14636	0.122111	2.74E-04	0.066657
GOTERM_BP_FAT	GO:0001975~response to amphetamine	7	1.263538	7.43E-05	549	19	13528	9.078324	0.233861	5.32E-04	0.136297
Annotation Cluster 49	Enrichment Score: 7.330546930917812										
Category	Term	Count	%	PValue	List Total	Pop Hits	Pop Total	Fold Enrichment	Bonferroni	Benjamini	FDR
GOTERM_BP_FAT	GO:0043269~regulation of ion transport	28	5.054152	1.22E-15	549	99	13528	6.969219	4.38E-12	2.68E-14	2.24E-12
GOTERM_BP_FAT	GO:0010959~regulation of metal ion transport	23	4.151625	6.11E-13	549	81	13528	6.996874	2.19E-09	1.17E-11	1.12E-09
GOTERM_BP_FAT	GO:0051924~regulation of calcium ion transport	20	3.610108	1.80E-11	549	69	13528	7.142367	6.44E-08	3.00E-10	3.30E-08
GOTERM_BP_FAT	GO:0043270~positive regulation of ion transport	14	2.527076	5.95E-09	549	42	13528	8.213722	2.13E-05	7.38E-08	1.09E-05
GOTERM_BP_FAT	GO:0032846~positive regulation of homeostatic process	13	2.34657	3.43E-08	549	40	13528	8.008379	1.23E-04	3.82E-07	6.29E-05
GOTERM_BP_FAT	GO:0043271~negative regulation of ion transport	10	1.805054	1.35E-07	549	23	13528	10.71355	4.85E-04	1.42E-06	2.48E-04

GOTERM_BP_FAT	GO:0010522~regulation of calcium ion transport into cytosol	10	1.805054	1.33E-06	549	29	13528	8.496954	0.004768	1.24E-05	0.002447
GOTERM_BP_FAT	GO:0051928~positive regulation of calcium ion transport	10	1.805054	4.44E-06	549	33	13528	7.46702	0.015793	3.80E-05	0.00815
GOTERM_BP_FAT	GO:0051279~regulation of release of sequestered calcium ion into cytosol	6	1.083032	2.29E-04	549	15	13528	9.856466	0.560785	0.001525	0.420361
GOTERM_BP_FAT	GO:0010524~positive regulation of calcium ion transport into cytosol	6	1.083032	0.001001	549	20	13528	7.39235	0.972449	0.005909	1.822105
GOTERM_BP_FAT	GO:0051281~positive regulation of release of sequestered calcium ion into cytosol	4	0.722022	0.004625	549	9	13528	10.95163	1	0.024368	8.160688
Annotation Cluster 50	Enrichment Score: 7.056924432465223										
Category	Term	Count	%	PValue	List Total	Pop Hits	Pop Total	Fold Enrichment	Bonferroni	Benjamini	FDR
GOTERM_BP_FAT	GO:0040013~negative regulation of locomotion	16	2.888087	1.35E-08	549	61	13528	6.463257	4.84E-05	1.60E-07	2.48E-05
GOTERM_BP_FAT	GO:0051271~negative regulation of cell motion	16	2.888087	2.17E-08	549	63	13528	6.258074	7.77E-05	2.46E-07	3.98E-05
GOTERM_BP_FAT	GO:0030336~negative regulation of cell migration	13	2.34657	2.31E-06	549	57	13528	5.619915	0.008252	2.07E-05	0.004242
Annotation Cluster 51	Enrichment Score: 7.01469187443571										
Category	Term	Count	%	PValue	List Total	Pop Hits	Pop Total	Fold Enrichment	Bonferroni	Benjamini	FDR
GOTERM_BP_FAT	GO:0007611~learning or memory	32	5.776173	3.88E-18	549	111	13528	7.103759	1.39E-14	9.60E-17	7.12E-15
GOTERM_BP_FAT	GO:0050877~neurological system process	87	15.70397	1.42E-07	549	1210	13528	1.77172	5.11E-04	1.49E-06	2.61E-04
GOTERM_BP_FAT	GO:0050890~cognition	59	10.64982	3.58E-04	549	909	13528	1.599372	0.722776	0.002292	0.654693
GOTERM_BP_FAT	GO:0007600~sensory perception	35	6.31769	0.442196	549	810	13528	1.064742	1	0.896032	99.99779
Annotation Cluster 52	Enrichment Score: 7.0028247306687215										
Category	Term	Count	%	PValue	List Total	Pop Hits	Pop Total	Fold Enrichment	Bonferroni	Benjamini	FDR
GOTERM_BP_FAT	GO:0006937~regulation of muscle contraction	18	3.249097	2.84E-09	549	72	13528	6.160291	1.02E-05	3.74E-08	5.22E-06
GOTERM_BP_FAT	GO:0019229~regulation of vasoconstriction	13	2.34657	4.20E-09	549	34	13528	9.421622	1.51E-05	5.40E-08	7.72E-06
GOTERM_BP_FAT	GO:0006940~regulation of smooth muscle contraction	13	2.34657	1.79E-08	549	38	13528	8.429872	6.41E-05	2.06E-07	3.28E-05
GOTERM_BP_FAT	GO:0045987~positive regulation of smooth muscle contraction	8	1.444043	3.71E-06	549	18	13528	10.95163	0.013231	3.23E-05	0.006819
GOTERM_BP_FAT	GO:0045933~positive regulation of muscle contraction	8	1.444043	1.22E-05	549	21	13528	9.387111	0.042819	9.77E-05	0.022403
Annotation Cluster 53	Enrichment Score: 6.867866604356317										
Category	Term	Count	%	PValue	List Total	Pop Hits	Pop Total	Fold Enrichment	Bonferroni	Benjamini	FDR
GOTERM_BP_FAT	GO:0007612~learning	18	3.249097	1.28E-10	549	60	13528	7.39235	4.58E-07	1.99E-09	2.35E-07
GOTERM_BP_FAT	GO:0048167~regulation of synaptic plasticity	17	3.068592	3.40E-09	549	64	13528	6.54531	1.22E-05	4.42E-08	6.25E-06
GOTERM_BP_FAT	GO:0008542~visual learning	10	1.805054	6.71E-07	549	27	13528	9.126358	0.002402	6.45E-06	0.001231

GOTERM_BP_FAT	GO:0048169~regulation of long-term neuronal synaptic plasticity	9	1.624549	8.79E-07	549	21	13528	10.5605	0.003148	8.30E-06	0.001614
GOTERM_BP_FAT	GO:0007632~visual behavior	10	1.805054	2.49E-06	549	31	13528	7.948763	0.008907	2.20E-05	0.004581
GOTERM_BP_FAT	GO:0048168~regulation of neuronal synaptic plasticity	10	1.805054	9.72E-06	549	36	13528	6.844768	0.034256	7.90E-05	0.017845
Annotation Cluster 54	Enrichment Score: 6.454859793989394										
Category	Term	Count	%	PValue	List Total	Pop Hits	Pop Total	Fold Enrichment	Bonferroni	Benjamini	FDR
GOTERM_MF_FAT	GO:0046983~protein dimerization activity	55	9.927798	5.57E-10	523	542	12983	2.51905	4.34E-07	2.17E-08	8.52E-07
GOTERM_MF_FAT	GO:0042802~identical protein binding	55	9.927798	1.72E-07	523	640	12983	2.13332	1.34E-04	3.72E-06	2.63E-04
GOTERM_MF_FAT	GO:0042803~protein homodimerization activity	28	5.054152	4.51E-04	523	334	12983	2.081062	0.296526	0.00428	0.688264
Annotation Cluster 55	Enrichment Score: 6.278148281374902										
Category	Term	Count	%	PValue	List Total	Pop Hits	Pop Total	Fold Enrichment	Bonferroni	Benjamini	FDR
GOTERM_BP_FAT	GO:0055088~lipid homeostasis	16	2.888087	8.87E-10	549	51	13528	7.730562	3.18E-06	1.24E-08	1.63E-06
GOTERM_BP_FAT	GO:0015908~fatty acid transport	12	2.166065	5.00E-09	549	28	13528	10.5605	1.80E-05	6.30E-08	9.19E-06
GOTERM_BP_FAT	GO:0046717~acid secretion	10	1.805054	9.65E-09	549	18	13528	13.68954	3.46E-05	1.17E-07	1.77E-05
GOTERM_BP_FAT	GO:0010876~lipid localization	25	4.512635	1.90E-08	549	157	13528	3.923753	6.83E-05	2.18E-07	3.50E-05
GOTERM_BP_FAT	GO:0050482~arachidonic acid secretion	8	1.444043	4.94E-08	549	11	13528	17.92085	1.77E-04	5.40E-07	9.07E-05
GOTERM_BP_FAT	GO:0032309~icosanoid secretion	8	1.444043	4.94E-08	549	11	13528	17.92085	1.77E-04	5.40E-07	9.07E-05
GOTERM_BP_FAT	GO:0015909~long-chain fatty acid transport	10	1.805054	5.23E-08	549	21	13528	11.73389	1.88E-04	5.70E-07	9.61E-05
GOTERM_BP_FAT	GO:0006869~lipid transport	23	4.151625	8.55E-08	549	145	13528	3.908599	3.07E-04	9.13E-07	1.57E-04
GOTERM_BP_FAT	GO:0030301~cholesterol transport	11	1.98556	1.42E-06	549	37	13528	7.325752	0.005082	1.32E-05	0.002609
GOTERM_BP_FAT	GO:0015918~sterol transport	11	1.98556	1.42E-06	549	37	13528	7.325752	0.005082	1.32E-05	0.002609
GOTERM_BP_FAT	GO:0042632~cholesterol homeostasis	11	1.98556	1.86E-06	549	38	13528	7.132969	0.006644	1.68E-05	0.003413
GOTERM_BP_FAT	GO:0055092~sterol homeostasis	11	1.98556	1.86E-06	549	38	13528	7.132969	0.006644	1.68E-05	0.003413
GOTERM_BP_FAT	GO:0015718~monocarboxylic acid transport	12	2.166065	3.87E-06	549	50	13528	5.91388	0.01378	3.36E-05	0.007104
GOTERM_BP_FAT	GO:0046942~carboxylic acid transport	13	2.34657	0.016348	549	147	13528	2.179151	1	0.076989	26.11852
GOTERM_BP_FAT	GO:0015849~organic acid transport	13	2.34657	0.017162	549	148	13528	2.164427	1	0.080486	27.23319
Annotation Cluster 56	Enrichment Score: 6.217568871595432										
Category	Term	Count	%	PValue	List Total	Pop Hits	Pop Total	Fold Enrichment	Bonferroni	Benjamini	FDR
GOTERM_BP_FAT	GO:0009894~regulation of catabolic process	25	4.512635	3.96E-13	549	96	13528	6.41697	1.42E-09	7.76E-12	7.27E-10

GOTERM_BP_FAT	GO:0031329~regulation of cellular catabolic process	19	3.429603	1.70E-11	549	61	13528	7.675117	6.09E-08	2.84E-10	3.12E-08
GOTERM_BP_FAT	GO:0031331~positive regulation of cellular catabolic process	15	2.707581	4.54E-11	549	36	13528	10.26715	1.63E-07	7.30E-10	8.33E-08
GOTERM_BP_FAT	GO:0009896~positive regulation of catabolic process	16	2.888087	4.73E-10	549	49	13528	8.046095	1.70E-06	6.75E-09	8.68E-07
GOTERM_BP_FAT	GO:0050994~regulation of lipid catabolic process	12	2.166065	1.94E-09	549	26	13528	11.37285	6.95E-06	2.60E-08	3.56E-06
GOTERM_BP_FAT	GO:0010906~regulation of glucose metabolic process	13	2.34657	6.16E-09	549	35	13528	9.152433	2.21E-05	7.62E-08	1.13E-05
GOTERM_BP_FAT	GO:0008286~insulin receptor signaling pathway	13	2.34657	1.27E-08	549	37	13528	8.657707	4.55E-05	1.52E-07	2.33E-05
GOTERM_BP_FAT	GO:0010907~positive regulation of glucose metabolic process	10	1.805054	1.77E-08	549	19	13528	12.96903	6.34E-05	2.05E-07	3.25E-05
GOTERM_BP_FAT	GO:0010675~regulation of cellular carbohydrate metabolic process	13	2.34657	1.79E-08	549	38	13528	8.429872	6.41E-05	2.06E-07	3.28E-05
GOTERM_BP_FAT	GO:0006109~regulation of carbohydrate metabolic process	13	2.34657	2.49E-08	549	39	13528	8.213722	8.93E-05	2.82E-07	4.57E-05
GOTERM_BP_FAT	GO:0045913~positive regulation of carbohydrate metabolic process	10	1.805054	3.10E-08	549	20	13528	12.32058	1.11E-04	3.47E-07	5.69E-05
GOTERM_BP_FAT	GO:0010676~positive regulation of cellular carbohydrate metabolic process	10	1.805054	3.10E-08	549	20	13528	12.32058	1.11E-04	3.47E-07	5.69E-05
GOTERM_BP_FAT	GO:0010827~regulation of glucose transport	12	2.166065	5.34E-08	549	34	13528	8.696882	1.92E-04	5.79E-07	9.81E-05
GOTERM_BP_FAT	GO:0043467~regulation of generation of precursor metabolites and energy	11	1.98556	1.58E-07	549	30	13528	9.035094	5.68E-04	1.65E-06	2.91E-04
GOTERM_BP_FAT	GO:0042593~glucose homeostasis	13	2.34657	4.05E-07	549	49	13528	6.537452	0.001452	3.99E-06	7.44E-04
GOTERM_BP_FAT	GO:0033500~carbohydrate homeostasis	13	2.34657	4.05E-07	549	49	13528	6.537452	0.001452	3.99E-06	7.44E-04
GOTERM_BP_FAT	GO:0046324~regulation of glucose import	11	1.98556	4.37E-07	549	33	13528	8.213722	0.001566	4.29E-06	8.02E-04
GOTERM_BP_FAT	GO:0043255~regulation of carbohydrate biosynthetic process	9	1.624549	8.79E-07	549	21	13528	10.5605	0.003148	8.30E-06	0.001614
GOTERM_BP_FAT	GO:0010828~positive regulation of glucose transport	9	1.624549	1.33E-06	549	22	13528	10.08048	0.004768	1.24E-05	0.002447
GOTERM_BP_FAT	GO:0046326~positive regulation of glucose import	9	1.624549	1.33E-06	549	22	13528	10.08048	0.004768	1.24E-05	0.002447
GOTERM_BP_FAT	GO:0032881~regulation of polysaccharide metabolic process	7	1.263538	1.57E-05	549	15	13528	11.49921	0.054892	1.23E-04	0.028901
GOTERM_BP_FAT	GO:0045821~positive regulation of glycolysis	5	0.902527	8.46E-05	549	7	13528	17.60083	0.261624	6.00E-04	0.155166
GOTERM_BP_FAT	GO:0032885~regulation of polysaccharide biosynthetic process	6	1.083032	1.58E-04	549	14	13528	10.5605	0.432952	0.001084	0.290035
GOTERM_BP_FAT	GO:0005979~regulation of glycogen biosynthetic process	6	1.083032	1.58E-04	549	14	13528	10.5605	0.432952	0.001084	0.290035
GOTERM_BP_FAT	GO:0010962~regulation of glucan biosynthetic process	6	1.083032	1.58E-04	549	14	13528	10.5605	0.432952	0.001084	0.290035
GOTERM_BP_FAT	GO:0031998~regulation of fatty acid beta-oxidation	5	0.902527	1.64E-04	549	8	13528	15.40073	0.44411	0.00112	0.30018
GOTERM_BP_FAT	GO:0043470~regulation of carbohydrate catabolic process	6	1.083032	3.23E-04	549	16	13528	9.240437	0.685617	0.002087	0.590693
GOTERM_BP_FAT	GO:0043471~regulation of cellular carbohydrate catabolic process	6	1.083032	3.23E-04	549	16	13528	9.240437	0.685617	0.002087	0.590693
GOTERM_BP_FAT	GO:0045725~positive regulation of glycogen biosynthetic process	5	0.902527	4.60E-04	549	10	13528	12.32058	0.808125	0.002897	0.841685

GOTERM_BP_FAT	GO:0046320~regulation of fatty acid oxidation	7	1.263538	7.56E-04	549	28	13528	6.160291	0.933593	0.004563	1.378892
GOTERM_BP_FAT	GO:0006110~regulation of glycolysis	5	0.902527	0.001017	549	12	13528	10.26715	0.973961	0.005982	1.850479
GOTERM_BP_FAT	GO:0032148~activation of protein kinase B activity	3	0.541516	0.022043	549	6	13528	12.32058	1	0.100612	33.59233

Annotation Cluster 57	Enrichment Score: 6.213931495432237										
Category	Term	Count	%	PValue	List Total	Pop Hits	Pop Total	Fold Enrichment	Bonferroni	Benjamini	FDR
GOTERM_BP_FAT	GO:0002684~positive regulation of immune system process	43	7.761733	4.76E-16	549	238	13528	4.451975	1.59E-12	9.88E-15	8.10E-13
GOTERM_BP_FAT	GO:0050865~regulation of cell activation	35	6.31769	1.61E-14	549	175	13528	4.928233	5.77E-11	3.36E-13	2.96E-11
GOTERM_BP_FAT	GO:0050867~positive regulation of cell activation	26	4.693141	1.62E-12	549	111	13528	5.771805	5.82E-09	2.98E-11	2.98E-09
GOTERM_BP_FAT	GO:0002694~regulation of leukocyte activation	30	5.415162	2.27E-11	549	166	13528	4.453223	8.13E-08	3.73E-10	4.16E-08
GOTERM_BP_FAT	GO:0002696~positive regulation of leukocyte activation	23	4.151625	1.96E-10	549	106	13528	5.346668	7.02E-07	2.96E-09	3.59E-07
GOTERM_BP_FAT	GO:0051249~regulation of lymphocyte activation	27	4.873646	2.19E-10	549	148	13528	4.495348	7.86E-07	3.30E-09	4.03E-07
GOTERM_BP_FAT	GO:0051251~positive regulation of lymphocyte activation	22	3.971119	2.21E-10	549	97	13528	5.588718	7.92E-07	3.31E-09	4.06E-07
GOTERM_BP_FAT	GO:0050670~regulation of lymphocyte proliferation	19	3.429603	4.21E-09	549	83	13528	5.640749	1.51E-05	5.39E-08	7.73E-06
GOTERM_BP_FAT	GO:0070663~regulation of leukocyte proliferation	19	3.429603	5.16E-09	549	84	13528	5.573597	1.85E-05	6.45E-08	9.47E-06
GOTERM_BP_FAT	GO:0032944~regulation of mononuclear cell proliferation	19	3.429603	5.16E-09	549	84	13528	5.573597	1.85E-05	6.45E-08	9.47E-06
GOTERM_BP_FAT	GO:0002683~negative regulation of immune system process	17	3.068592	1.76E-07	549	83	13528	5.046986	6.32E-04	1.83E-06	3.24E-04
GOTERM_BP_FAT	GO:0050863~regulation of T cell activation	20	3.610108	2.19E-07	549	117	13528	4.212165	7.86E-04	2.24E-06	4.02E-04
GOTERM_BP_FAT	GO:0050671~positive regulation of lymphocyte proliferation	13	2.34657	1.54E-06	549	55	13528	5.824276	0.005519	1.42E-05	0.002834
GOTERM_BP_FAT	GO:0050870~positive regulation of T cell activation	15	2.707581	1.84E-06	549	76	13528	4.863388	0.006579	1.68E-05	0.00338
GOTERM_BP_FAT	GO:0070665~positive regulation of leukocyte proliferation	13	2.34657	1.89E-06	549	56	13528	5.720271	0.006765	1.71E-05	0.003476
GOTERM_BP_FAT	GO:0032946~positive regulation of mononuclear cell proliferation	13	2.34657	1.89E-06	549	56	13528	5.720271	0.006765	1.71E-05	0.003476
GOTERM_BP_FAT	GO:0050871~positive regulation of B cell activation	10	1.805054	4.44E-06	549	33	13528	7.46702	0.015793	3.80E-05	0.00815
GOTERM_BP_FAT	GO:0042129~regulation of T cell proliferation	13	2.34657	5.86E-06	549	62	13528	5.166696	0.020818	4.89E-05	0.01077
GOTERM_BP_FAT	GO:0050866~negative regulation of cell activation	12	2.166065	2.89E-05	549	61	13528	4.847442	0.09851	2.21E-04	0.053082
GOTERM_BP_FAT	GO:0050864~regulation of B cell activation	11	1.98556	3.13E-05	549	51	13528	5.314761	0.106056	2.37E-04	0.057384
GOTERM_BP_FAT	GO:0042102~positive regulation of T cell proliferation	9	1.624549	1.39E-04	549	39	13528	5.686423	0.393669	9.62E-04	0.255835
GOTERM_BP_FAT	GO:0070664~negative regulation of leukocyte proliferation	8	1.444043	2.40E-04	549	32	13528	6.160291	0.576666	0.001588	0.439135
GOTERM_BP_FAT	GO:0032945~negative regulation of mononuclear cell proliferation	8	1.444043	2.40E-04	549	32	13528	6.160291	0.576666	0.001588	0.439135

GOTERM_BP_FAT	GO:0050672~negative regulation of lymphocyte proliferation	8	1.444043	2.40E-04	549	32	13528	6.160291	0.576666	0.001588	0.439135
GOTERM_BP_FAT	GO:0051250~negative regulation of lymphocyte activation	10	1.805054	2.87E-04	549	54	13528	4.563179	0.642506	0.001868	0.525265
GOTERM_BP_FAT	GO:0002695~negative regulation of leukocyte activation	10	1.805054	4.36E-04	549	57	13528	4.323012	0.790391	0.002752	0.796796
GOTERM_BP_FAT	GO:0030888~regulation of B cell proliferation	6	1.083032	0.004857	549	28	13528	5.28025	1	0.025502	8.552828
GOTERM_BP_FAT	GO:0050868~negative regulation of T cell activation	7	1.263538	0.00652	549	42	13528	4.106861	1	0.033385	11.3192
GOTERM_BP_FAT	GO:0046006~regulation of activated T cell proliferation	4	0.722022	0.011062	549	12	13528	8.213722	1	0.054129	18.47696
GOTERM_BP_FAT	GO:0042130~negative regulation of T cell proliferation	5	0.902527	0.022298	549	27	13528	4.563179	1	0.101598	33.9094
GOTERM_BP_FAT	GO:0030890~positive regulation of B cell proliferation	4	0.722022	0.045138	549	20	13528	4.928233	1	0.188759	57.18375
Annotation Cluster 58	Enrichment Score: 6.1916037062181415										
Category	Term	Count	%	PValue	List Total	Pop Hits	Pop Total	Fold Enrichment	Bonferroni	Benjamini	FDR
GOTERM_BP_FAT	GO:0040008~regulation of growth	47	8.483755	5.35E-13	549	341	13528	3.39629	1.92E-09	1.03E-11	9.82E-10
GOTERM_BP_FAT	GO:0008361~regulation of cell size	29	5.234657	1.79E-08	549	206	13528	3.468902	6.43E-05	2.06E-07	3.29E-05
GOTERM_BP_FAT	GO:0001558~regulation of cell growth	27	4.873646	7.77E-08	549	194	13528	3.429441	2.79E-04	8.32E-07	1.43E-04
GOTERM_BP_FAT	GO:0032535~regulation of cellular component size	32	5.776173	1.79E-07	549	271	13528	2.909658	6.41E-04	1.84E-06	3.28E-04
GOTERM_BP_FAT	GO:0045792~negative regulation of cell size	14	2.527076	1.74E-04	549	99	13528	3.484609	0.464233	0.001179	0.319
GOTERM_BP_FAT	GO:0030308~negative regulation of cell growth	12	2.166065	0.001199	549	92	13528	3.214065	0.986496	0.006998	2.179894
GOTERM_BP_FAT	GO:0045926~negative regulation of growth	13	2.34657	0.001639	549	110	13528	2.912138	0.997219	0.009312	2.968039
Annotation Cluster 59	Enrichment Score: 6.159851029931304										
Category	Term	Count	%	PValue	List Total	Pop Hits	Pop Total	Fold Enrichment	Bonferroni	Benjamini	FDR
GOTERM_BP_FAT	GO:0032583~regulation of gene-specific transcription	34	6.137184	2.05E-17	549	134	13528	6.252236	7.36E-14	4.88E-16	3.77E-14
GOTERM_BP_FAT	GO:0010605~negative regulation of macromolecule metabolic process	72	12.99639	4.94E-12	549	734	13528	2.417117	1.77E-08	8.65E-11	9.08E-09
GOTERM_BP_FAT	GO:0010551~regulation of specific transcription from RNA polymerase II promoter	21	3.790614	8.27E-10	549	94	13528	5.504941	2.96E-06	1.16E-08	1.52E-06
GOTERM_BP_FAT	GO:0032582~negative regulation of gene-specific transcription	15	2.707581	3.68E-09	549	48	13528	7.700364	1.32E-05	4.76E-08	6.75E-06
GOTERM_BP_FAT	GO:0010553~negative regulation of specific transcription from RNA polymerase II promoter	14	2.527076	4.26E-09	549	41	13528	8.414057	1.53E-05	5.44E-08	7.83E-06
GOTERM_BP_FAT	GO:0009890~negative regulation of biosynthetic process	53	9.566787	3.86E-08	549	573	13528	2.2792	1.38E-04	4.26E-07	7.08E-05
GOTERM_BP_FAT	GO:0031327~negative regulation of cellular biosynthetic process	49	8.844765	7.54E-07	549	561	13528	2.152259	0.002701	7.21E-06	0.001385
GOTERM_BP_FAT	GO:0010558~negative regulation of macromolecule biosynthetic process	45	8.122744	1.09E-05	549	547	13528	2.027153	0.038292	8.81E-05	0.019988
GOTERM_BP_FAT	GO:0051172~negative regulation of nitrogen compound metabolic process	41	7.400722	7.23E-05	549	519	13528	1.946605	0.228343	5.18E-04	0.132627

GOTERM_BP_FAT	GO:0045934~negative regulation of nucleobase, nucleoside, nucleotide and nucleic acid metabolic process	40	7.220217	1.13E-04	549	512	13528	1.925091	0.332679	7.85E-04	0.206877
GOTERM_BP_FAT	GO:0010629~negative regulation of gene expression	39	7.039711	1.70E-04	549	504	13528	1.906757	0.456871	0.001158	0.312034
GOTERM_BP_FAT	GO:0000122~negative regulation of transcription from RNA polymerase II promoter	25	4.512635	2.10E-04	549	266	13528	2.315899	0.528936	0.001406	0.384663
GOTERM_BP_FAT	GO:0016481~negative regulation of transcription	35	6.31769	5.13E-04	549	459	13528	1.878956	0.841066	0.003199	0.937264
GOTERM_BP_FAT	GO:0045892~negative regulation of transcription, DNA-dependent	29	5.234657	6.36E-04	549	356	13528	2.007286	0.897868	0.003892	1.161299
GOTERM_BP_FAT	GO:0051253~negative regulation of RNA metabolic process	29	5.234657	8.13E-04	549	362	13528	1.974016	0.945859	0.004873	1.481952
GOTERM_MF_FAT	GO:0016564~transcription repressor activity	19	3.429603	0.086407	523	316	12983	1.492588	1	0.347312	74.90079
Annotation Cluster 60	Enrichment Score: 6.125201502997545										
Category	Term	Count	%	PValue	List Total	Pop Hits	Pop Total	Fold Enrichment	Bonferroni	Benjamini	FDR
GOTERM_BP_FAT	GO:0051048~negative regulation of secretion	16	2.888087	2.85E-09	549	55	13528	7.168339	1.02E-05	3.73E-08	5.23E-06
GOTERM_BP_FAT	GO:0046883~regulation of hormone secretion	16	2.888087	4.25E-08	549	66	13528	5.973616	1.53E-04	4.67E-07	7.81E-05
GOTERM_BP_FAT	GO:0046888~negative regulation of hormone secretion	6	1.083032	0.003474	549	26	13528	5.686423	0.999996	0.018682	6.191788
Annotation Cluster 61	Enrichment Score: 6.100394632183115										
Category	Term	Count	%	PValue	List Total	Pop Hits	Pop Total	Fold Enrichment	Bonferroni	Benjamini	FDR
GOTERM_BP_FAT	GO:0032103~positive regulation of response to external stimulus	28	5.054152	2.67E-21	549	64	13528	10.78051	9.58E-18	8.33E-20	4.91E-18
GOTERM_BP_FAT	GO:0050795~regulation of behavior	18	3.249097	9.79E-13	549	46	13528	9.642195	3.51E-09	1.84E-11	1.80E-09
GOTERM_BP_FAT	GO:0050920~regulation of chemotaxis	15	2.707581	3.82E-12	549	31	13528	11.92314	1.37E-08	6.79E-11	7.02E-09
GOTERM_BP_FAT	GO:0050921~positive regulation of chemotaxis	14	2.527076	2.57E-11	549	29	13528	11.89574	9.22E-08	4.21E-10	4.72E-08
GOTERM_BP_FAT	GO:0048520~positive regulation of behavior	14	2.527076	2.92E-10	549	34	13528	10.14636	1.05E-06	4.29E-09	5.36E-07
GOTERM_BP_FAT	GO:0002685~regulation of leukocyte migration	10	1.805054	3.10E-08	549	20	13528	12.32058	1.11E-04	3.47E-07	5.69E-05
GOTERM_BP_FAT	GO:0002687~positive regulation of leukocyte migration	8	1.444043	8.35E-07	549	15	13528	13.14196	0.002991	7.92E-06	0.001534
GOTERM_BP_FAT	GO:0050926~regulation of positive chemotaxis	8	1.444043	5.67E-06	549	19	13528	10.37523	0.020149	4.77E-05	0.010421
GOTERM_BP_FAT	GO:0050927~positive regulation of positive chemotaxis	8	1.444043	5.67E-06	549	19	13528	10.37523	0.020149	4.77E-05	0.010421
GOTERM_BP_FAT	GO:0032642~regulation of chemokine production	7	1.263538	1.57E-05	549	15	13528	11.49921	0.054892	1.23E-04	0.028901
GOTERM_BP_FAT	GO:0002690~positive regulation of leukocyte chemotaxis	6	1.083032	2.28E-05	549	10	13528	14.7847	0.078497	1.76E-04	0.041846
GOTERM_BP_FAT	GO:0002688~regulation of leukocyte chemotaxis	6	1.083032	4.04E-05	549	11	13528	13.44064	0.134876	3.00E-04	0.074151
GOTERM_BP_FAT	GO:0032722~positive regulation of chemokine production	5	0.902527	8.46E-05	549	7	13528	17.60083	0.261624	6.00E-04	0.155166
GOTERM_BP_FAT	GO:0002444~myeloid leukocyte mediated immunity	6	1.083032	2.29E-04	549	15	13528	9.856466	0.560785	0.001525	0.420361

GOTERM_MF_FAT	GO:0005138~interleukin-6 receptor binding	4	0.722022	0.002004	523	7	12983	14.1852	0.790371	0.016484	3.021368
GOTERM_BP_FAT	GO:0002446~neutrophil mediated immunity	4	0.722022	0.004625	549	9	13528	10.95163	1	0.024368	8.160688
GOTERM_BP_FAT	GO:0032755~positive regulation of interleukin-6 production	5	0.902527	0.006353	549	19	13528	6.484517	1	0.032637	11.04587
GOTERM_BP_FAT	GO:0002443~leukocyte mediated immunity	10	1.805054	0.007917	549	86	13528	2.865252	1	0.040025	13.5822
GOTERM_BP_FAT	GO:0002252~immune effector process	12	2.166065	0.020216	549	134	13528	2.206672	1	0.09306	31.27649
GOTERM_BP_FAT	GO:0050930~induction of positive chemotaxis	3	0.541516	0.059419	549	10	13528	7.39235	1	0.238358	67.53433

Annotation Cluster 62 Enrichment Score: 6.047770212784773

Category	Term	Count	%	PValue	List Total	Pop Hits	Pop Total	Fold Enrichment	Bonferroni	Benjamini	FDR
GOTERM_BP_FAT	GO:0030030~cell projection organization	49	8.844765	5.79E-13	549	368	13528	3.281025	2.08E-09	1.11E-11	1.06E-09
GOTERM_BP_FAT	GO:0031175~neuron projection development	35	6.31769	9.68E-10	549	256	13528	3.368909	3.47E-06	1.34E-08	1.78E-06
GOTERM_BP_FAT	GO:0048666~neuron development	38	6.859206	3.96E-08	549	339	13528	2.762137	1.42E-04	4.36E-07	7.27E-05
GOTERM_BP_FAT	GO:0030182~neuron differentiation	44	7.942238	6.89E-08	549	438	13528	2.475368	2.47E-04	7.42E-07	1.26E-04
GOTERM_BP_FAT	GO:0000904~cell morphogenesis involved in differentiation	30	5.415162	1.97E-07	549	244	13528	3.029652	7.06E-04	2.02E-06	3.61E-04
GOTERM_BP_FAT	GO:0048812~neuron projection morphogenesis	26	4.693141	1.74E-06	549	213	13528	3.007842	0.006214	1.59E-05	0.003192
GOTERM_BP_FAT	GO:0032989~cellular component morphogenesis	37	6.6787	5.26E-06	549	397	13528	2.296532	0.018696	4.45E-05	0.009662
GOTERM_BP_FAT	GO:0000902~cell morphogenesis	34	6.137184	8.32E-06	549	356	13528	2.35337	0.029415	6.82E-05	0.015285
GOTERM_BP_FAT	GO:0007409~axonogenesis	23	4.151625	1.16E-05	549	193	13528	2.936512	0.04075	9.37E-05	0.021298
GOTERM_BP_FAT	GO:0032990~cell part morphogenesis	27	4.873646	1.56E-05	549	256	13528	2.598873	0.054378	1.22E-04	0.028622
GOTERM_BP_FAT	GO:0048858~cell projection morphogenesis	26	4.693141	2.09E-05	549	245	13528	2.614981	0.072134	1.62E-04	0.038324
GOTERM_BP_FAT	GO:0048667~cell morphogenesis involved in neuron differentiation	23	4.151625	4.04E-05	549	209	13528	2.711707	0.134769	3.00E-04	0.074087
GOTERM_BP_FAT	GO:0007411~axon guidance	9	1.624549	0.068679	549	107	13528	2.072621	1	0.270273	72.9285

Annotation Cluster 63 Enrichment Score: 5.887009055169513

Category	Term	Count	%	PValue	List Total	Pop Hits	Pop Total	Fold Enrichment	Bonferroni	Benjamini	FDR
GOTERM_BP_FAT	GO:0006939~smooth muscle contraction	12	2.166065	3.75E-08	549	33	13528	8.960424	1.34E-04	4.15E-07	6.89E-05
GOTERM_BP_FAT	GO:0003012~muscle system process	22	3.971119	4.31E-06	549	168	13528	3.226819	0.015355	3.70E-05	0.007922
GOTERM_BP_FAT	GO:0006936~muscle contraction	20	3.610108	1.35E-05	549	153	13528	3.221067	0.047246	1.07E-04	0.024776

Annotation Cluster 64 Enrichment Score: 5.882961757998917

Category	Term	Count	%	PValue	List Total	Pop Hits	Pop Total	Fold Enrichment	Bonferroni	Benjamini	FDR

GOTERM_BP_FAT	GO:0051130~positive regulation of cellular component organization	32	5.776173	7.82E-12	549	181	13528	4.356449	2.81E-08	1.34E-10	1.44E-08
GOTERM_BP_FAT	GO:0051726~regulation of cell cycle	40	7.220217	1.92E-09	549	331	13528	2.977784	6.90E-06	2.59E-08	3.53E-06
GOTERM_BP_FAT	GO:0045787~positive regulation of cell cycle	16	2.888087	4.90E-09	549	57	13528	6.916818	1.76E-05	6.23E-08	9.00E-06
GOTERM_BP_FAT	GO:0010638~positive regulation of organelle organization	18	3.249097	2.82E-08	549	83	13528	5.343867	1.01E-04	3.18E-07	5.19E-05
GOTERM_BP_FAT	GO:0045428~regulation of nitric oxide biosynthetic process	10	1.805054	6.71E-07	549	27	13528	9.126358	0.002402	6.45E-06	0.001231
GOTERM_BP_FAT	GO:0007346~regulation of mitotic cell cycle	22	3.971119	8.36E-07	549	152	13528	3.566485	0.002993	7.91E-06	0.001535
GOTERM_BP_FAT	GO:0045429~positive regulation of nitric oxide biosynthetic process	9	1.624549	8.79E-07	549	21	13528	10.5605	0.003148	8.30E-06	0.001614
GOTERM_BP_FAT	GO:0033043~regulation of organelle organization	26	4.693141	2.45E-06	549	217	13528	2.952398	0.008739	2.17E-05	0.004494
GOTERM_BP_FAT	GO:0051785~positive regulation of nuclear division	8	1.444043	4.38E-05	549	25	13528	7.885173	0.145357	3.24E-04	0.080386
GOTERM_BP_FAT	GO:0045840~positive regulation of mitosis	8	1.444043	4.38E-05	549	25	13528	7.885173	0.145357	3.24E-04	0.080386
GOTERM_BP_FAT	GO:0007088~regulation of mitosis	9	1.624549	0.001761	549	56	13528	3.960187	0.998206	0.009971	3.185494
GOTERM_BP_FAT	GO:0051783~regulation of nuclear division	9	1.624549	0.001761	549	56	13528	3.960187	0.998206	0.009971	3.185494
GOTERM_BP_FAT	GO:0010564~regulation of cell cycle process	13	2.34657	0.002225	549	114	13528	2.809957	0.999661	0.012349	4.008087

Annotation Cluster 65 Enrichment Score: 5.770500942461988

Category	Term	Count	%	PValue	List Total	Pop Hits	Pop Total	Fold Enrichment	Bonferroni	Benjamini	FDR
GOTERM_BP_FAT	GO:0051329~interphase of mitotic cell cycle	23	4.151625	1.08E-10	549	103	13528	5.502396	3.89E-07	1.71E-09	1.99E-07
GOTERM_BP_FAT	GO:0051325~interphase	23	4.151625	1.96E-10	549	106	13528	5.346668	7.02E-07	2.96E-09	3.59E-07
GOTERM_BP_FAT	GO:0000082~G1/S transition of mitotic cell cycle	14	2.527076	2.62E-07	549	56	13528	6.160291	9.38E-04	2.62E-06	4.80E-04
GOTERM_BP_FAT	GO:0000278~mitotic cell cycle	33	5.956679	4.48E-05	549	370	13528	2.197726	0.148471	3.30E-04	0.082254
GOTERM_BP_FAT	GO:0022402~cell cycle process	42	7.581227	2.13E-04	549	565	13528	1.831733	0.533858	0.001423	0.390021
GOTERM_BP_FAT	GO:0022403~cell cycle phase	33	5.956679	3.53E-04	549	414	13528	1.964151	0.718252	0.002268	0.646458
GOTERM_BP_FAT	GO:0007049~cell cycle	49	8.844765	0.002165	549	776	13528	1.55595	0.99958	0.012074	3.902262

Annotation Cluster 66 Enrichment Score: 5.633853698993006

Category	Term	Count	%	PValue	List Total	Pop Hits	Pop Total	Fold Enrichment	Bonferroni	Benjamini	FDR
GOTERM_CC_FAT	GO:0016323~basolateral plasma membrane	31	5.595668	7.48E-10	520	203	12782	3.753714	2.91E-07	1.08E-08	1.04E-06
GOTERM_CC_FAT	GO:0005925~focal adhesion	18	3.249097	6.66E-07	520	102	12782	4.337783	2.59E-04	6.65E-06	9.26E-04
GOTERM_CC_FAT	GO:0005924~cell-substrate adherens junction	18	3.249097	1.17E-06	520	106	12782	4.174093	4.54E-04	1.11E-05	0.00162
GOTERM_CC_FAT	GO:0005912~adherens junction	22	3.971119	1.19E-06	520	155	12782	3.488883	4.62E-04	1.10E-05	0.001651

GOTERM_CC_FAT	GO:0030055~cell-substrate junction	18	3.249097	2.56E-06	520	112	12782	3.950481	9.96E-04	2.21E-05	0.003559
GOTERM_CC_FAT	GO:0070161~anchoring junction	22	3.971119	6.44E-06	520	172	12782	3.144052	0.0025	5.44E-05	0.00894
GOTERM_CC_FAT	GO:0030054~cell junction	31	5.595668	0.032066	520	518	12782	1.47105	0.999997	0.12744	36.41194

Annotation Cluster 67	Enrichment Score: 5.6325751725469955										
Category	Term	Count	%	PValue	List Total	Pop Hits	Pop Total	Fold Enrichment	Bonferroni	Benjamini	FDR
GOTERM_MF_FAT	GO:0004620~phospholipase activity	22	3.971119	7.91E-12	523	83	12983	6.57988	6.16E-09	4.40E-10	1.21E-08
GOTERM_MF_FAT	GO:0016298~lipase activity	22	3.971119	3.48E-10	523	100	12983	5.4613	2.71E-07	1.43E-08	5.32E-07
GOTERM_BP_FAT	GO:0016042~lipid catabolic process	24	4.33213	5.07E-07	549	173	13528	3.418428	0.001816	4.94E-06	9.30E-04
GOTERM_BP_FAT	GO:0006644~phospholipid metabolic process	22	3.971119	2.92E-05	549	190	13528	2.853188	0.099322	2.22E-04	0.053543
GOTERM_MF_FAT	GO:0004623~phospholipase A2 activity	8	1.444043	5.51E-05	523	26	12983	7.638182	0.042008	7.15E-04	0.084234
GOTERM_BP_FAT	GO:0019637~organophosphate metabolic process	22	3.971119	6.23E-05	549	200	13528	2.710528	0.200395	4.51E-04	0.114434
GOTERM_MF_FAT	GO:0047498~calcium-dependent phospholipase A2 activity	5	0.902527	1.59E-04	523	8	12983	15.51506	0.116462	0.001718	0.242838
GOTERM_MF_FAT	GO:0004091~carboxylesterase activity	9	1.624549	0.03922	523	96	12983	2.327259	1	0.186499	45.77369

Annotation Cluster 68	Enrichment Score: 5.624879859386411										
Category	Term	Count	%	PValue	List Total	Pop Hits	Pop Total	Fold Enrichment	Bonferroni	Benjamini	FDR
GOTERM_BP_FAT	GO:0010883~regulation of lipid storage	11	1.98556	2.59E-09	549	21	13528	12.90728	9.28E-06	3.42E-08	4.75E-06
GOTERM_BP_FAT	GO:0010885~regulation of cholesterol storage	7	1.263538	1.67E-06	549	11	13528	15.68074	0.005972	1.53E-05	0.003067
GOTERM_BP_FAT	GO:0010884~positive regulation of lipid storage	6	1.083032	1.18E-05	549	9	13528	16.42744	0.041402	9.48E-05	0.021646
GOTERM_BP_FAT	GO:0010886~positive regulation of cholesterol storage	4	0.722022	6.22E-04	549	5	13528	19.71293	0.892632	0.003814	1.135995

Annotation Cluster 69	Enrichment Score: 5.569484567425592										
Category	Term	Count	%	PValue	List Total	Pop Hits	Pop Total	Fold Enrichment	Bonferroni	Benjamini	FDR
GOTERM_MF_FAT	GO:0019955~cytokine binding	25	4.512635	6.43E-12	523	109	12983	5.693599	5.01E-09	3.85E-10	9.83E-09
GOTERM_MF_FAT	GO:0019956~chemokine binding	12	2.166065	2.90E-09	523	27	12983	11.03293	2.26E-06	9.43E-08	4.44E-06
GOTERM_MF_FAT	GO:0004950~chemokine receptor activity	11	1.98556	1.92E-08	523	25	12983	10.9226	1.50E-05	4.68E-07	2.94E-05
GOTERM_MF_FAT	GO:0019958~C-X-C chemokine binding	6	1.083032	1.14E-05	523	9	12983	16.54939	0.008808	1.70E-04	0.017371
GOTERM_MF_FAT	GO:0016494~C-X-C chemokine receptor activity	5	0.902527	1.59E-04	523	8	12983	15.51506	0.116462	0.001718	0.242838
GOTERM_MF_FAT	GO:0019957~C-C chemokine binding	6	1.083032	3.11E-04	523	16	12983	9.309034	0.215393	0.003145	0.47518
GOTERM_MF_FAT	GO:0016493~C-C chemokine receptor activity	6	1.083032	3.11E-04	523	16	12983	9.309034	0.215393	0.003145	0.47518

GOTERM_MF_FAT	GO:0015026~coreceptor activity	4	0.722022	0.044298	523	20	12983	4.964818	1	0.204825	49.99581
Annotation Cluster 70	Enrichment Score: 5.47248431859889										
Category	Term	Count	%	PValue	List Total	Pop Hits	Pop Total	Fold Enrichment	Bonferroni	Benjamini	FDR
GOTERM_BP_FAT	GO:0010594~regulation of endothelial cell migration	11	1.98556	2.07E-08	549	25	13528	10.84211	7.42E-05	2.36E-07	3.80E-05
GOTERM_BP_FAT	GO:0043535~regulation of blood vessel endothelial cell migration	9	1.624549	6.65E-08	549	16	13528	13.86066	2.39E-04	7.19E-07	1.22E-04
GOTERM_BP_FAT	GO:0010595~positive regulation of endothelial cell migration	8	1.444043	2.39E-07	549	13	13528	15.16379	8.57E-04	2.42E-06	4.39E-04
GOTERM_BP_FAT	GO:0030336~negative regulation of cell migration	13	2.34657	2.31E-06	549	57	13528	5.619915	0.008252	2.07E-05	0.004242
GOTERM_BP_FAT	GO:0043536~positive regulation of blood vessel endothelial cell migration	6	1.083032	1.18E-05	549	9	13528	16.42744	0.041402	9.48E-05	0.021646
GOTERM_BP_FAT	GO:0043537~negative regulation of blood vessel endothelial cell migration	5	0.902527	2.85E-04	549	9	13528	13.68954	0.640544	0.001862	0.522478
GOTERM_BP_FAT	GO:0010596~negative regulation of endothelial cell migration	5	0.902527	0.001927	549	14	13528	8.800416	0.999011	0.01082	3.480288
Annotation Cluster 71	Enrichment Score: 5.471458812147765										
Category	Term	Count	%	PValue	List Total	Pop Hits	Pop Total	Fold Enrichment	Bonferroni	Benjamini	FDR
GOTERM_MF_FAT	GO:0001608~nucleotide receptor activity, G-protein coupled	10	1.805054	1.25E-06	523	29	12983	8.560032	9.73E-04	2.21E-05	0.001911
GOTERM_MF_FAT	GO:0045028~purinergic nucleotide receptor activity, G-protein coupled	10	1.805054	1.25E-06	523	29	12983	8.560032	9.73E-04	2.21E-05	0.001911
GOTERM_MF_FAT	GO:0001614~purinergic nucleotide receptor activity	10	1.805054	9.13E-06	523	36	12983	6.895581	0.007087	1.39E-04	0.013964
GOTERM_MF_FAT	GO:0016502~nucleotide receptor activity	10	1.805054	9.13E-06	523	36	12983	6.895581	0.007087	1.39E-04	0.013964
Annotation Cluster 72	Enrichment Score: 5.355811158525234										
Category	Term	Count	%	PValue	List Total	Pop Hits	Pop Total	Fold Enrichment	Bonferroni	Benjamini	FDR
GOTERM_BP_FAT	GO:0046883~regulation of hormone secretion	16	2.888087	4.25E-08	549	66	13528	5.973616	1.53E-04	4.67E-07	7.81E-05
GOTERM_BP_FAT	GO:0002791~regulation of peptide secretion	11	1.98556	1.20E-05	549	46	13528	5.892453	0.042008	9.60E-05	0.02197
GOTERM_BP_FAT	GO:0050796~regulation of insulin secretion	9	1.624549	1.68E-04	549	40	13528	5.544262	0.453116	0.001147	0.308518
Annotation Cluster 73	Enrichment Score: 5.333463773198416										
Category	Term	Count	%	PValue	List Total	Pop Hits	Pop Total	Fold Enrichment	Bonferroni	Benjamini	FDR
GOTERM_BP_FAT	GO:0045637~regulation of myeloid cell differentiation	18	3.249097	1.78E-09	549	70	13528	6.3363	6.39E-06	2.43E-08	3.27E-06
GOTERM_BP_FAT	GO:0002761~regulation of myeloid leukocyte differentiation	14	2.527076	4.26E-09	549	41	13528	8.414057	1.53E-05	5.44E-08	7.83E-06
GOTERM_BP_FAT	GO:0045639~positive regulation of myeloid cell differentiation	12	2.166065	3.75E-08	549	33	13528	8.960424	1.34E-04	4.15E-07	6.89E-05
GOTERM_BP_FAT	GO:0045670~regulation of osteoclast differentiation	8	1.444043	1.73E-05	549	22	13528	8.960424	0.060075	1.35E-04	0.031715
GOTERM_BP_FAT	GO:0002763~positive regulation of myeloid leukocyte differentiation	7	1.263538	5.26E-05	549	18	13528	9.582676	0.17196	3.86E-04	0.096563

GOTERM_BP_FAT	GO:0045671~negative regulation of osteoclast differentiation	5	0.902527	2.85E-04	549	9	13528	13.68954	0.640544	0.001862	0.522478
GOTERM_BP_FAT	GO:0002762~negative regulation of myeloid leukocyte differentiation	6	1.083032	4.42E-04	549	17	13528	8.696882	0.795075	0.002787	0.808273
GOTERM_BP_FAT	GO:0045638~negative regulation of myeloid cell differentiation	6	1.083032	0.006589	549	30	13528	4.928233	1	0.033641	11.43349

Annotation Cluster 74	Enrichment Score: 5.332429596533481										
Category	Term	Count	%	PValue	List Total	Pop Hits	Pop Total	Fold Enrichment	Bonferroni	Benjamini	FDR
GOTERM_BP_FAT	GO:0051098~regulation of binding	23	4.151625	2.26E-07	549	153	13528	3.704228	8.10E-04	2.29E-06	4.15E-04
GOTERM_BP_FAT	GO:0051091~positive regulation of transcription factor activity	14	2.527076	6.16E-07	549	60	13528	5.749605	0.002208	5.97E-06	0.001131
GOTERM_BP_FAT	GO:0051090~regulation of transcription factor activity	18	3.249097	7.51E-07	549	103	13528	4.306223	0.002689	7.20E-06	0.001378
GOTERM_BP_FAT	GO:0043388~positive regulation of DNA binding	14	2.527076	3.90E-06	549	70	13528	4.928233	0.013905	3.38E-05	0.007169
GOTERM_BP_FAT	GO:0051101~regulation of DNA binding	18	3.249097	7.34E-06	549	121	13528	3.665628	0.02599	6.05E-05	0.013482
GOTERM_BP_FAT	GO:0051099~positive regulation of binding	14	2.527076	1.35E-05	549	78	13528	4.422773	0.047197	1.07E-04	0.02475
GOTERM_BP_FAT	GO:0051092~positive regulation of NF-kappaB transcription factor activity	8	1.444043	0.001168	549	41	13528	4.808032	0.984862	0.006824	2.122645

Annotation Cluster 75	Enrichment Score: 4.920021413355671										
Category	Term	Count	%	PValue	List Total	Pop Hits	Pop Total	Fold Enrichment	Bonferroni	Benjamini	FDR
GOTERM_BP_FAT	GO:0007160~cell-matrix adhesion	17	3.068592	4.80E-07	549	89	13528	4.70674	0.001722	4.70E-06	8.82E-04
GOTERM_BP_FAT	GO:0031589~cell-substrate adhesion	17	3.068592	1.84E-06	549	98	13528	4.274488	0.006582	1.67E-05	0.003381
GOTERM_BP_FAT	GO:0007044~cell-substrate junction assembly	6	1.083032	0.001964	549	23	13528	6.42813	0.999136	0.011012	3.546871

Annotation Cluster 76	Enrichment Score: 4.892935329798202										
Category	Term	Count	%	PValue	List Total	Pop Hits	Pop Total	Fold Enrichment	Bonferroni	Benjamini	FDR
GOTERM_BP_FAT	GO:0030522~intracellular receptor-mediated signaling pathway	18	3.249097	5.56E-09	549	75	13528	5.91388	1.99E-05	6.92E-08	1.02E-05
GOTERM_MF_FAT	GO:0051427~hormone receptor binding	19	3.429603	9.96E-09	523	88	12983	5.359747	7.76E-06	2.59E-07	1.52E-05
GOTERM_BP_FAT	GO:0030518~steroid hormone receptor signaling pathway	15	2.707581	5.32E-08	549	58	13528	6.372715	1.91E-04	5.78E-07	9.77E-05
GOTERM_MF_FAT	GO:0035257~nuclear hormone receptor binding	13	2.34657	5.32E-05	523	77	12983	4.19108	0.040619	7.03E-04	0.081391
GOTERM_BP_FAT	GO:0030521~androgen receptor signaling pathway	9	1.624549	7.64E-05	549	36	13528	6.160291	0.239579	5.44E-04	0.140126
GOTERM_MF_FAT	GO:0003712~transcription cofactor activity	31	5.595668	1.51E-04	523	363	12983	2.119964	0.110837	0.001677	0.230407
GOTERM_MF_FAT	GO:0003713~transcription coactivator activity	21	3.790614	4.00E-04	523	214	12983	2.436009	0.267859	0.00389	0.610343
GOTERM_MF_FAT	GO:0050681~androgen receptor binding	6	1.083032	0.003363	523	26	12983	5.728637	0.927475	0.025897	5.021655
GOTERM_MF_FAT	GO:0035258~steroid hormone receptor binding	7	1.263538	0.003787	523	38	12983	4.572859	0.947968	0.028842	5.638943

Annotation Cluster 77	Enrichment Score: 4.82107877685285										
Category	Term	Count	%	PValue	List Total	Pop Hits	Pop Total	Fold Enrichment	Bonferroni	Benjamini	FDR
GOTERM_BP_FAT	GO:0003018~vascular process in circulatory system	15	2.707581	5.32E-08	549	58	13528	6.372715	1.91E-04	5.78E-07	9.77E-05
GOTERM_BP_FAT	GO:0050880~regulation of blood vessel size	13	2.34657	1.01E-06	549	53	13528	6.04406	0.003618	9.49E-06	0.001855
GOTERM_BP_FAT	GO:0035150~regulation of tube size	13	2.34657	1.01E-06	549	53	13528	6.04406	0.003618	9.49E-06	0.001855
GOTERM_BP_FAT	GO:0042311~vasodilation	6	1.083032	0.003474	549	26	13528	5.686423	0.999996	0.018682	6.191788
GOTERM_BP_FAT	GO:0042310~vasoconstriction	5	0.902527	0.00416	549	17	13528	7.247402	1	0.022102	7.369469
Annotation Cluster 78	Enrichment Score: 4.771247074650847										
Category	Term	Count	%	PValue	List Total	Pop Hits	Pop Total	Fold Enrichment	Bonferroni	Benjamini	FDR
GOTERM_BP_FAT	GO:0051899~membrane depolarization	14	2.527076	8.21E-09	549	43	13528	8.022705	2.95E-05	1.00E-07	1.51E-05
GOTERM_BP_FAT	GO:0042391~regulation of membrane potential	22	3.971119	9.53E-08	549	134	13528	4.045565	3.42E-04	1.01E-06	1.75E-04
GOTERM_BP_FAT	GO:0060078~regulation of postsynaptic membrane potential	8	1.444043	2.40E-05	549	23	13528	8.57084	0.082376	1.84E-04	0.044005
GOTERM_BP_FAT	GO:0060079~regulation of excitatory postsynaptic membrane potential	7	1.263538	7.43E-05	549	19	13528	9.078324	0.233861	5.32E-04	0.136297
GOTERM_MF_FAT	GO:0046873~metal ion transmembrane transporter activity	4	0.722022	0.999863	523	328	12983	0.302733	1	1	100
Annotation Cluster 79	Enrichment Score: 4.731574303055107										
Category	Term	Count	%	PValue	List Total	Pop Hits	Pop Total	Fold Enrichment	Bonferroni	Benjamini	FDR
GOTERM_BP_FAT	GO:0030036~actin cytoskeleton organization	26	4.693141	5.10E-06	549	226	13528	2.834824	0.018128	4.34E-05	0.009366
GOTERM_BP_FAT	GO:0030029~actin filament-based process	27	4.873646	5.25E-06	549	241	13528	2.760629	0.018656	4.45E-05	0.009641
GOTERM_BP_FAT	GO:0007010~cytoskeleton organization	39	7.039711	7.23E-06	549	436	13528	2.204141	0.025599	5.98E-05	0.013276
GOTERM_BP_FAT	GO:0007015~actin filament organization	11	1.98556	6.12E-04	549	72	13528	3.764623	0.888795	0.003773	1.118223
Annotation Cluster 80	Enrichment Score: 4.71128278655516										
Category	Term	Count	%	PValue	List Total	Pop Hits	Pop Total	Fold Enrichment	Bonferroni	Benjamini	FDR
GOTERM_BP_FAT	GO:0001836~release of cytochrome c from mitochondria	10	1.805054	5.23E-08	549	21	13528	11.73389	1.88E-04	5.70E-07	9.61E-05
GOTERM_BP_FAT	GO:0008637~apoptotic mitochondrial changes	11	1.98556	2.25E-07	549	31	13528	8.743639	8.08E-04	2.30E-06	4.14E-04
GOTERM_BP_FAT	GO:0007005~mitochondrion organization	15	2.707581	0.001418	549	138	13528	2.678388	0.993836	0.008148	2.571839
GOTERM_BP_FAT	GO:0046902~regulation of mitochondrial membrane permeability	4	0.722022	0.008551	549	11	13528	8.960424	1	0.04287	14.58998
Annotation Cluster 81	Enrichment Score: 4.640963991896973										
Category	Term	Count	%	PValue	List Total	Pop Hits	Pop Total	Fold Enrichment	Bonferroni	Benjamini	FDR

GOTERM_BP_FAT	GO:0050994~regulation of lipid catabolic process	12	2.166065	1.94E-09	549	26	13528	11.37285	6.95E-06	2.60E-08	3.56E-06
GOTERM_BP_FAT	GO:0045923~positive regulation of fatty acid metabolic process	10	1.805054	5.23E-08	549	21	13528	11.73389	1.88E-04	5.70E-07	9.61E-05
GOTERM_BP_FAT	GO:0050996~positive regulation of lipid catabolic process	6	1.083032	1.05E-04	549	13	13528	11.37285	0.314233	7.34E-04	0.192944
GOTERM_BP_FAT	GO:0031998~regulation of fatty acid beta-oxidation	5	0.902527	1.64E-04	549	8	13528	15.40073	0.44411	0.00112	0.30018
GOTERM_BP_FAT	GO:0046321~positive regulation of fatty acid oxidation	5	0.902527	1.64E-04	549	8	13528	15.40073	0.44411	0.00112	0.30018
GOTERM_BP_FAT	GO:0046320~regulation of fatty acid oxidation	7	1.263538	7.56E-04	549	28	13528	6.160291	0.933593	0.004563	1.378892
GOTERM_BP_FAT	GO:0032000~positive regulation of fatty acid beta-oxidation	3	0.541516	0.015098	549	5	13528	14.7847	1	0.071837	24.37517
Annotation Cluster 82	Enrichment Score: 4.5919457889582915										
Category	Term	Count	%	PValue	List Total	Pop Hits	Pop Total	Fold Enrichment	Bonferroni	Benjamini	FDR
GOTERM_MF_FAT	GO:0008201~heparin binding	18	3.249097	6.72E-07	523	103	12983	4.338191	5.23E-04	1.28E-05	0.001028
GOTERM_MF_FAT	GO:0005539~glycosaminoglycan binding	20	3.610108	3.24E-06	523	140	12983	3.546299	0.002519	5.25E-05	0.004953
GOTERM_MF_FAT	GO:0030247~polysaccharide binding	20	3.610108	1.33E-05	523	154	12983	3.223908	0.010277	1.88E-04	0.020283
GOTERM_MF_FAT	GO:0001871~pattern binding	20	3.610108	1.33E-05	523	154	12983	3.223908	0.010277	1.88E-04	0.020283
GOTERM_MF_FAT	GO:0030246~carbohydrate binding	23	4.151625	0.028667	523	354	12983	1.612865	1	0.144665	35.9119
Annotation Cluster 83	Enrichment Score: 4.501228896207419										
Category	Term	Count	%	PValue	List Total	Pop Hits	Pop Total	Fold Enrichment	Bonferroni	Benjamini	FDR
GOTERM_BP_FAT	GO:0046579~positive regulation of Ras protein signal transduction	8	1.444043	1.43E-06	549	16	13528	12.32058	0.005127	1.33E-05	0.002632
GOTERM_BP_FAT	GO:0051057~positive regulation of small GTPase mediated signal transduction	8	1.444043	2.35E-06	549	17	13528	11.59584	0.008398	2.09E-05	0.004318
GOTERM_BP_FAT	GO:0035025~positive regulation of Rho protein signal transduction	3	0.541516	0.009307	549	4	13528	18.48087	1	0.046271	15.77949
Annotation Cluster 84	Enrichment Score: 4.498210995417102										
Category	Term	Count	%	PValue	List Total	Pop Hits	Pop Total	Fold Enrichment	Bonferroni	Benjamini	FDR
GOTERM_BP_FAT	GO:0033555~multicellular organismal response to stress	12	2.166065	2.60E-07	549	39	13528	7.581897	9.33E-04	2.61E-06	4.78E-04
GOTERM_BP_FAT	GO:0042596~fear response	7	1.263538	7.43E-05	549	19	13528	9.078324	0.233861	5.32E-04	0.136297
GOTERM_BP_FAT	GO:0002209~behavioral defense response	6	1.083032	2.29E-04	549	15	13528	9.856466	0.560785	0.001525	0.420361
GOTERM_BP_FAT	GO:0001662~behavioral fear response	6	1.083032	2.29E-04	549	15	13528	9.856466	0.560785	0.001525	0.420361
Annotation Cluster 85	Enrichment Score: 4.3132190831057535										
Category	Term	Count	%	PValue	List Total	Pop Hits	Pop Total	Fold Enrichment	Bonferroni	Benjamini	FDR
GOTERM_BP_FAT	GO:0034381~lipoprotein particle clearance	9	1.624549	6.65E-08	549	16	13528	13.86066	2.39E-04	7.19E-07	1.22E-04

Category	Term	Count	%	PValue	List Total	Pop Hits	Pop Total	Fold Enrichment	Bonferroni	Benjamini	FDR
GOTERM_MF_FAT	GO:0008034~lipoprotein binding	9	1.624549	5.83E-05	523	35	12983	6.383338	0.04439	7.32E-04	0.089118
GOTERM_MF_FAT	GO:0008035~high-density lipoprotein binding	3	0.541516	0.029623	523	7	12983	10.6389	1	0.147308	36.87032

Annotation Cluster 86	Enrichment Score: 4.309742284467744										
Category	Term	Count	%	PValue	List Total	Pop Hits	Pop Total	Fold Enrichment	Bonferroni	Benjamini	FDR
GOTERM_BP_FAT	GO:0051130~positive regulation of cellular component organization	32	5.776173	7.82E-12	549	181	13528	4.356449	2.81E-08	1.34E-10	1.44E-08
GOTERM_BP_FAT	GO:0010638~positive regulation of organelle organization	18	3.249097	2.82E-08	549	83	13528	5.343867	1.01E-04	3.18E-07	5.19E-05
GOTERM_BP_FAT	GO:0044087~regulation of cellular component biogenesis	21	3.790614	1.12E-06	549	142	13528	3.644116	0.004002	1.05E-05	0.002053
GOTERM_BP_FAT	GO:0033043~regulation of organelle organization	26	4.693141	2.45E-06	549	217	13528	2.952398	0.008739	2.17E-05	0.004494
GOTERM_BP_FAT	GO:0051495~positive regulation of cytoskeleton organization	9	1.624549	3.95E-04	549	45	13528	4.928233	0.75731	0.002521	0.722338
GOTERM_BP_FAT	GO:0051492~regulation of stress fiber formation	6	1.083032	7.76E-04	549	19	13528	7.781421	0.938296	0.00467	1.415976
GOTERM_BP_FAT	GO:0051496~positive regulation of stress fiber formation	5	0.902527	0.001017	549	12	13528	10.26715	0.973961	0.005982	1.850479
GOTERM_BP_FAT	GO:0032231~regulation of actin filament bundle formation	6	1.083032	0.00127	549	21	13528	7.040333	0.989531	0.007374	2.307272
GOTERM_BP_FAT	GO:0032233~positive regulation of actin filament bundle formation	5	0.902527	0.001927	549	14	13528	8.800416	0.999011	0.01082	3.480288
GOTERM_BP_FAT	GO:0051493~regulation of cytoskeleton organization	14	2.527076	0.003498	549	136	13528	2.536591	0.999997	0.018778	6.23221
GOTERM_BP_FAT	GO:0032956~regulation of actin cytoskeleton organization	10	1.805054	0.009842	549	89	13528	2.76867	1	0.048608	16.61018
GOTERM_BP_FAT	GO:0032970~regulation of actin filament-based process	10	1.805054	0.0121	549	92	13528	2.678388	1	0.058768	20.03458

Annotation Cluster 87	Enrichment Score: 4.252364611489874										
Category	Term	Count	%	PValue	List Total	Pop Hits	Pop Total	Fold Enrichment	Bonferroni	Benjamini	FDR
GOTERM_BP_FAT	GO:0043408~regulation of MAPKKK cascade	25	4.512635	7.65E-12	549	109	13528	5.651644	2.74E-08	1.33E-10	1.40E-08
GOTERM_BP_FAT	GO:0080135~regulation of cellular response to stress	15	2.707581	6.75E-05	549	103	13528	3.588519	0.215066	4.86E-04	0.123904
GOTERM_BP_FAT	GO:0070302~regulation of stress-activated protein kinase signaling pathway	12	2.166065	9.41E-05	549	69	13528	4.28542	0.286463	6.64E-04	0.172658
GOTERM_BP_FAT	GO:0046328~regulation of JNK cascade	11	1.98556	2.63E-04	549	65	13528	4.170043	0.611141	0.001725	0.482426
GOTERM_BP_FAT	GO:0043507~positive regulation of JUN kinase activity	5	0.902527	0.039031	549	32	13528	3.850182	1	0.166333	51.865
GOTERM_BP_FAT	GO:0043506~regulation of JUN kinase activity	5	0.902527	0.061319	549	37	13528	3.329887	1	0.244913	68.71825

Annotation Cluster 88	Enrichment Score: 4.2392572868266605										
Category	Term	Count	%	PValue	List Total	Pop Hits	Pop Total	Fold Enrichment	Bonferroni	Benjamini	FDR
GOTERM_BP_FAT	GO:0046890~regulation of lipid biosynthetic process	13	2.34657	1.46E-07	549	45	13528	7.118559	5.25E-04	1.53E-06	2.69E-04
GOTERM_BP_FAT	GO:0019218~regulation of steroid metabolic process	9	1.624549	7.64E-05	549	36	13528	6.160291	0.239579	5.44E-04	0.140126

GOTERM_BP_FAT	GO:0050810~regulation of steroid biosynthetic process	5	0.902527	0.017124	549	25	13528	4.928233	1	0.08042	27.18208

Annotation Cluster 89 Enrichment Score: 4.09179697062315

Category	Term	Count	%	PValue	List Total	Pop Hits	Pop Total	Fold Enrichment	Bonferroni	Benjamini	FDR
GOTERM_BP_FAT	GO:0052547~regulation of peptidase activity	15	2.707581	8.37E-06	549	86	13528	4.297878	0.02959	6.84E-05	0.015377
GOTERM_BP_FAT	GO:0043281~regulation of caspase activity	14	2.527076	1.55E-05	549	79	13528	4.366789	0.054238	1.22E-04	0.028547
GOTERM_BP_FAT	GO:0052548~regulation of endopeptidase activity	14	2.527076	2.35E-05	549	82	13528	4.207028	0.080886	1.81E-04	0.043174
GOTERM_BP_FAT	GO:0006919~activation of caspase activity	10	1.805054	2.87E-04	549	54	13528	4.563179	0.642506	0.001868	0.525265
GOTERM_BP_FAT	GO:0043280~positive regulation of caspase activity	10	1.805054	5.66E-04	549	59	13528	4.176469	0.868851	0.003502	1.034673
GOTERM_BP_FAT	GO:0010952~positive regulation of peptidase activity	10	1.805054	5.66E-04	549	59	13528	4.176469	0.868851	0.003502	1.034673

Annotation Cluster 90 Enrichment Score: 4.04249849447222

Category	Term	Count	%	PValue	List Total	Pop Hits	Pop Total	Fold Enrichment	Bonferroni	Benjamini	FDR
GOTERM_BP_FAT	GO:0007259~JAK-STAT cascade	11	1.98556	2.41E-06	549	39	13528	6.950072	0.008606	2.14E-05	0.004425
GOTERM_BP_FAT	GO:0007262~STAT protein nuclear translocation	4	0.722022	2.57E-04	549	4	13528	24.64117	0.601555	0.001684	0.470016
GOTERM_BP_FAT	GO:0007260~tyrosine phosphorylation of STAT protein	4	0.722022	0.001206	549	6	13528	16.42744	0.986831	0.007027	2.192472

Annotation Cluster 91 Enrichment Score: 4.031243560578616

Category	Term	Count	%	PValue	List Total	Pop Hits	Pop Total	Fold Enrichment	Bonferroni	Benjamini	FDR
GOTERM_BP_FAT	GO:0051970~negative regulation of transmission of nerve impulse	9	1.624549	4.05E-06	549	25	13528	8.87082	0.014409	3.49E-05	0.007431
GOTERM_BP_FAT	GO:0051966~regulation of synaptic transmission, glutamatergic	8	1.444043	5.67E-06	549	19	13528	10.37523	0.020149	4.77E-05	0.010421
GOTERM_BP_FAT	GO:0031645~negative regulation of neurological system process	9	1.624549	1.04E-05	549	28	13528	7.920375	0.03676	8.47E-05	0.019173
GOTERM_BP_FAT	GO:0050805~negative regulation of synaptic transmission	7	1.263538	1.39E-04	549	21	13528	8.213722	0.391743	9.57E-04	0.254216
GOTERM_BP_FAT	GO:0001659~temperature homeostasis	7	1.263538	1.39E-04	549	21	13528	8.213722	0.391743	9.57E-04	0.254216
GOTERM_BP_FAT	GO:0051967~negative regulation of synaptic transmission, glutamatergic	4	0.722022	0.002048	549	7	13528	14.08067	0.999359	0.011441	3.694588
GOTERM_BP_FAT	GO:0043266~regulation of potassium ion transport	4	0.722022	0.00641	549	10	13528	9.856466	1	0.032875	11.13836

Annotation Cluster 92 Enrichment Score: 4.027581405855782

Category	Term	Count	%	PValue	List Total	Pop Hits	Pop Total	Fold Enrichment	Bonferroni	Benjamini	FDR
GOTERM_BP_FAT	GO:0001503~ossification	16	2.888087	6.09E-05	549	115	13528	3.428336	0.196222	4.42E-04	0.111772
GOTERM_BP_FAT	GO:0001501~skeletal system development	29	5.234657	1.03E-04	549	319	13528	2.240106	0.307907	7.17E-04	0.188252
GOTERM_BP_FAT	GO:0060348~bone development	16	2.888087	1.32E-04	549	123	13528	3.205355	0.377853	9.18E-04	0.242684

Annotation Cluster 93	Enrichment Score: 4.02197397074524										
Category	Term	Count	%	PValue	List Total	Pop Hits	Pop Total	Fold Enrichment	Bonferroni	Benjamini	FDR
GOTERM_BP_FAT	GO:0048871~multicellular organismal homeostasis	17	3.068592	2.49E-07	549	85	13528	4.928233	8.92E-04	2.51E-06	4.57E-04
GOTERM_BP_FAT	GO:0001894~tissue homeostasis	10	1.805054	9.24E-04	549	63	13528	3.911296	0.963699	0.005502	1.683374
GOTERM_BP_FAT	GO:0060249~anatomical structure homeostasis	12	2.166065	0.003736	549	106	13528	2.789566	0.999999	0.019989	6.643655

Annotation Cluster 94	Enrichment Score: 3.9947767632404467										
Category	Term	Count	%	PValue	List Total	Pop Hits	Pop Total	Fold Enrichment	Bonferroni	Benjamini	FDR
GOTERM_BP_FAT	GO:0043550~regulation of lipid kinase activity	6	1.083032	2.28E-05	549	10	13528	14.7847	0.078497	1.76E-04	0.041846
GOTERM_BP_FAT	GO:0043551~regulation of phosphoinositide 3-kinase activity	5	0.902527	3.74E-05	549	6	13528	20.5343	0.125666	2.81E-04	0.068733
GOTERM_BP_FAT	GO:0043552~positive regulation of phosphoinositide 3-kinase activity	5	0.902527	3.74E-05	549	6	13528	20.5343	0.125666	2.81E-04	0.068733
GOTERM_BP_FAT	GO:0030032~lamellipodium assembly	5	0.902527	0.003285	549	16	13528	7.700364	0.999993	0.017697	5.863647

Annotation Cluster 95	Enrichment Score: 3.989399797249202										
Category	Term	Count	%	PValue	List Total	Pop Hits	Pop Total	Fold Enrichment	Bonferroni	Benjamini	FDR
GOTERM_MF_FAT	GO:0008009~chemokine activity	10	1.805054	7.47E-05	523	46	12983	5.396542	0.056502	9.23E-04	0.114139
GOTERM_MF_FAT	GO:0005125~cytokine activity	21	3.790614	1.15E-04	523	195	12983	2.673364	0.085864	0.00138	0.176128
GOTERM_MF_FAT	GO:0042379~chemokine receptor binding	10	1.805054	1.25E-04	523	49	12983	5.066141	0.092834	0.001475	0.191129

Annotation Cluster 96	Enrichment Score: 3.9505417659338553										
Category	Term	Count	%	PValue	List Total	Pop Hits	Pop Total	Fold Enrichment	Bonferroni	Benjamini	FDR
GOTERM_BP_FAT	GO:0045923~positive regulation of fatty acid metabolic process	10	1.805054	5.23E-08	549	21	13528	11.73389	1.88E-04	5.70E-07	9.61E-05
GOTERM_BP_FAT	GO:0046889~positive regulation of lipid biosynthetic process	7	1.263538	1.39E-04	549	21	13528	8.213722	0.391743	9.57E-04	0.254216
GOTERM_BP_FAT	GO:0042304~regulation of fatty acid biosynthetic process	5	0.902527	0.002544	549	15	13528	8.213722	0.999892	0.014003	4.570779
GOTERM_BP_FAT	GO:0045723~positive regulation of fatty acid biosynthetic process	4	0.722022	0.008551	549	11	13528	8.960424	1	0.04287	14.58998

Annotation Cluster 97	Enrichment Score: 3.94611355356877										
Category	Term	Count	%	PValue	List Total	Pop Hits	Pop Total	Fold Enrichment	Bonferroni	Benjamini	FDR
GOTERM_BP_FAT	GO:0032880~regulation of protein localization	26	4.693141	2.48E-10	549	138	13528	4.642538	8.89E-07	3.71E-09	4.55E-07
GOTERM_BP_FAT	GO:0070201~regulation of establishment of protein localization	24	4.33213	4.83E-10	549	121	13528	4.887504	1.73E-06	6.88E-09	8.87E-07
GOTERM_BP_FAT	GO:0051223~regulation of protein transport	22	3.971119	5.00E-09	549	114	13528	4.755313	1.79E-05	6.34E-08	9.18E-06
GOTERM_BP_FAT	GO:0051222~positive regulation of protein transport	14	2.527076	2.33E-06	549	67	13528	5.1489	0.008331	2.08E-05	0.004283

GOTERM_BP_FAT	GO:0050708~regulation of protein secretion	13	2.34657	2.81E-06	549	58	13528	5.52302	0.010018	2.47E-05	0.005155
GOTERM_BP_FAT	GO:0046822~regulation of nucleocytoplasmic transport	11	1.98556	9.89E-05	549	58	13528	4.673325	0.298765	6.94E-04	0.181546
GOTERM_BP_FAT	GO:0033157~regulation of intracellular protein transport	10	1.805054	2.48E-04	549	53	13528	4.649277	0.588752	0.001629	0.453898
GOTERM_BP_FAT	GO:0032386~regulation of intracellular transport	11	1.98556	5.47E-04	549	71	13528	3.817645	0.859268	0.003387	0.998933
GOTERM_BP_FAT	GO:0032388~positive regulation of intracellular transport	7	1.263538	0.001112	549	30	13528	5.749605	0.981519	0.006511	2.022618
GOTERM_BP_FAT	GO:0050714~positive regulation of protein secretion	8	1.444043	0.001168	549	41	13528	4.808032	0.984862	0.006824	2.122645
GOTERM_BP_FAT	GO:0033158~regulation of protein import into nucleus, translocation	4	0.722022	0.004625	549	9	13528	10.95163	1	0.024368	8.160688
GOTERM_BP_FAT	GO:0042306~regulation of protein import into nucleus	7	1.263538	0.008197	549	44	13528	3.920185	1	0.041359	14.02934
GOTERM_BP_FAT	GO:0046824~positive regulation of nucleocytoplasmic transport	4	0.722022	0.05748	549	22	13528	4.480212	1	0.231858	66.28324
GOTERM_BP_FAT	GO:0042993~positive regulation of transcription factor import into nucleus	3	0.541516	0.095146	549	13	13528	5.686423	1	0.353229	84.05715
GOTERM_BP_FAT	GO:0042307~positive regulation of protein import into nucleus	3	0.541516	0.149469	549	17	13528	4.348441	1	0.494187	94.88599
GOTERM_BP_FAT	GO:0042990~regulation of transcription factor import into nucleus	3	0.541516	0.344529	549	30	13528	2.464117	1	0.812542	99.95724
Annotation Cluster 98	Enrichment Score: 3.9055374877140334										
Category	Term	Count	%	PValue	List Total	Pop Hits	Pop Total	Fold Enrichment	Bonferroni	Benjamini	FDR
GOTERM_BP_FAT	GO:0008016~regulation of heart contraction	16	2.888087	3.70E-07	549	77	13528	5.120242	0.001327	3.66E-06	6.80E-04
GOTERM_BP_FAT	GO:0003073~regulation of systemic arterial blood pressure	11	1.98556	8.05E-07	549	35	13528	7.744366	0.002885	7.68E-06	0.001479
GOTERM_BP_FAT	GO:0003044~regulation of systemic arterial blood pressure mediated by a chemical signal	9	1.624549	7.73E-06	549	27	13528	8.213722	0.027347	6.34E-05	0.014196
GOTERM_BP_FAT	GO:0002027~regulation of heart rate	8	1.444043	1.73E-05	549	22	13528	8.960424	0.060075	1.35E-04	0.031715
GOTERM_BP_FAT	GO:0001990~regulation of systemic arterial blood pressure by hormone	7	1.263538	1.02E-04	549	20	13528	8.624408	0.307613	7.18E-04	0.188035
GOTERM_BP_FAT	GO:0050886~endocrine process	7	1.263538	2.41E-04	549	23	13528	7.499485	0.578159	0.001585	0.440936
GOTERM_BP_FAT	GO:0010460~positive regulation of heart rate	5	0.902527	4.60E-04	549	10	13528	12.32058	0.808125	0.002897	0.841685
GOTERM_BP_FAT	GO:0045823~positive regulation of heart contraction	5	0.902527	0.001927	549	14	13528	8.800416	0.999011	0.01082	3.480288
GOTERM_BP_FAT	GO:0002016~regulation of blood volume by renin-angiotensin	4	0.722022	0.003178	549	8	13528	12.32058	0.999989	0.017178	5.678468
GOTERM_BP_FAT	GO:0001976~neurological system process involved in regulation of systemic arterial blood pressure	4	0.722022	0.004625	549	9	13528	10.95163	1	0.024368	8.160688
GOTERM_BP_FAT	GO:0003081~regulation of systemic arterial blood pressure by renin-angiotensin	4	0.722022	0.008551	549	11	13528	8.960424	1	0.04287	14.58998
Annotation Cluster 99	Enrichment Score: 3.8925629937989106										
Category	Term	Count	%	PValue	List Total	Pop Hits	Pop Total	Fold Enrichment	Bonferroni	Benjamini	FDR
GOTERM_CC_FAT	GO:0033267~axon part	13	2.34657	1.28E-06	520	54	12782	5.917593	4.97E-04	1.16E-05	0.001774

GOTERM_CC_FAT	GO:0043679~nerve terminal	9	1.624549	9.54E-05	520	37	12782	5.979106	0.036416	7.27E-04	0.132383
GOTERM_CC_FAT	GO:0043195~terminal button	5	0.902527	0.017252	520	25	12782	4.916154	0.998852	0.077431	21.47501

Annotation Cluster 100 Enrichment Score: 3.7369947031186803

Category	Term	Count	%	PValue	List Total	Pop Hits	Pop Total	Fold Enrichment	Bonferroni	Benjamini	FDR
GOTERM_BP_FAT	GO:0048762~mesenchymal cell differentiation	13	2.34657	6.47E-07	549	51	13528	6.281081	0.00232	6.26E-06	0.001189
GOTERM_BP_FAT	GO:0014031~mesenchymal cell development	13	2.34657	6.47E-07	549	51	13528	6.281081	0.00232	6.26E-06	0.001189
GOTERM_BP_FAT	GO:0060485~mesenchyme development	13	2.34657	8.11E-07	549	52	13528	6.160291	0.002905	7.72E-06	0.001489
GOTERM_BP_FAT	GO:0014032~neural crest cell development	8	1.444043	2.94E-04	549	33	13528	5.973616	0.651246	0.00191	0.53787
GOTERM_BP_FAT	GO:0014033~neural crest cell differentiation	8	1.444043	2.94E-04	549	33	13528	5.973616	0.651246	0.00191	0.53787
GOTERM_BP_FAT	GO:0001755~neural crest cell migration	6	1.083032	0.001964	549	23	13528	6.42813	0.999136	0.011012	3.546871
GOTERM_BP_FAT	GO:0001837~epithelial to mesenchymal transition	5	0.902527	0.005179	549	18	13528	6.844768	1	0.027019	9.095605
GOTERM_BP_FAT	GO:0001667~ameboidal cell migration	6	1.083032	0.015994	549	37	13528	3.995865	1	0.075473	25.62931
GOTERM_BP_FAT	GO:0014829~vascular smooth muscle contraction	3	0.541516	0.048817	549	9	13528	8.213722	1	0.201909	60.11391

Annotation Cluster 101 Enrichment Score: 3.7301293584219635

Category	Term	Count	%	PValue	List Total	Pop Hits	Pop Total	Fold Enrichment	Bonferroni	Benjamini	FDR
GOTERM_BP_FAT	GO:0046649~lymphocyte activation	29	5.234657	8.28E-09	549	199	13528	3.590924	2.97E-05	1.01E-07	1.52E-05
GOTERM_BP_FAT	GO:0042110~T cell activation	18	3.249097	1.27E-05	549	126	13528	3.520167	0.044612	1.01E-04	0.023363
GOTERM_BP_FAT	GO:0070661~leukocyte proliferation	10	1.805054	5.48E-05	549	44	13528	5.600265	0.178452	3.99E-04	0.100588
GOTERM_BP_FAT	GO:0032943~mononuclear cell proliferation	10	1.805054	5.48E-05	549	44	13528	5.600265	0.178452	3.99E-04	0.100588
GOTERM_BP_FAT	GO:0046651~lymphocyte proliferation	9	1.624549	2.40E-04	549	42	13528	5.28025	0.577935	0.001587	0.440666
GOTERM_BP_FAT	GO:0042098~T cell proliferation	6	1.083032	0.004124	549	27	13528	5.475815	1	0.02198	7.309068
GOTERM_BP_FAT	GO:0042100~B cell proliferation	4	0.722022	0.013954	549	13	13528	7.581897	1	0.066807	22.74595
GOTERM_BP_FAT	GO:0043393~regulation of protein binding	3	0.541516	0.329561	549	29	13528	2.549086	1	0.79679	99.93527

Annotation Cluster 102 Enrichment Score: 3.7207651209449235

Category	Term	Count	%	PValue	List Total	Pop Hits	Pop Total	Fold Enrichment	Bonferroni	Benjamini	FDR
GOTERM_MF_FAT	GO:0008083~growth factor activity	22	3.971119	1.91E-06	523	161	12983	3.392112	0.001485	3.30E-05	0.002919
GOTERM_BP_FAT	GO:0051781~positive regulation of cell division	9	1.624549	1.39E-04	549	39	13528	5.686423	0.393669	9.62E-04	0.255835
GOTERM_BP_FAT	GO:0051302~regulation of cell division	9	1.624549	5.36E-04	549	47	13528	4.718521	0.853997	0.003329	0.980293

Category	Term	Count	%	PValue	List Total	Pop Hits	Pop Total	Fold Enrichment	Bonferroni	Benjamini	FDR
GOTERM_MF_FAT	GO:0005172~vascular endothelial growth factor receptor binding	3	0.541516	0.009172	523	4	12983	18.61807	0.999237	0.059018	13.14657
Annotation Cluster 103	Enrichment Score: 3.677366879007439										
Category	Term	Count	%	PValue	List Total	Pop Hits	Pop Total	Fold Enrichment	Bonferroni	Benjamini	FDR
GOTERM_BP_FAT	GO:0030100~regulation of endocytosis	13	2.34657	4.91E-06	549	61	13528	5.251396	0.01745	4.18E-05	0.009013
GOTERM_BP_FAT	GO:0060627~regulation of vesicle-mediated transport	16	2.888087	6.75E-06	549	96	13528	4.106861	0.023917	5.59E-05	0.012393
GOTERM_BP_FAT	GO:0048259~regulation of receptor-mediated endocytosis	6	1.083032	3.23E-04	549	16	13528	9.240437	0.685617	0.002087	0.590693
GOTERM_BP_FAT	GO:0045806~negative regulation of endocytosis	5	0.902527	0.002544	549	15	13528	8.213722	0.999892	0.014003	4.570779
GOTERM_BP_FAT	GO:0048261~negative regulation of receptor-mediated endocytosis	3	0.541516	0.015098	549	5	13528	14.7847	1	0.071837	24.37517
Annotation Cluster 104	Enrichment Score: 3.3911598967029626										
Category	Term	Count	%	PValue	List Total	Pop Hits	Pop Total	Fold Enrichment	Bonferroni	Benjamini	FDR
GOTERM_BP_FAT	GO:0060284~regulation of cell development	25	4.512635	2.92E-06	549	205	13528	3.00502	0.010437	2.57E-05	0.005372
GOTERM_BP_FAT	GO:0051960~regulation of nervous system development	20	3.610108	2.98E-04	549	192	13528	2.566788	0.656594	0.001934	0.54574
GOTERM_BP_FAT	GO:0010720~positive regulation of cell development	10	1.805054	0.001785	549	69	13528	3.571183	0.998355	0.010091	3.228526
GOTERM_BP_FAT	GO:0050767~regulation of neurogenesis	14	2.527076	0.017514	549	166	13528	2.078171	1	0.081766	27.71113
Annotation Cluster 105	Enrichment Score: 3.3904511237337744										
Category	Term	Count	%	PValue	List Total	Pop Hits	Pop Total	Fold Enrichment	Bonferroni	Benjamini	FDR
GOTERM_BP_FAT	GO:0032368~regulation of lipid transport	15	2.707581	2.18E-12	549	30	13528	12.32058	7.80E-09	3.98E-11	4.00E-09
GOTERM_BP_FAT	GO:0055088~lipid homeostasis	16	2.888087	8.87E-10	549	51	13528	7.730562	3.18E-06	1.24E-08	1.63E-06
GOTERM_BP_FAT	GO:0034381~lipoprotein particle clearance	9	1.624549	6.65E-08	549	16	13528	13.86066	2.39E-04	7.19E-07	1.22E-04
GOTERM_BP_FAT	GO:0046486~glycerolipid metabolic process	24	4.33213	1.52E-07	549	162	13528	3.650543	5.45E-04	1.59E-06	2.79E-04
GOTERM_BP_FAT	GO:0032371~regulation of sterol transport	9	1.624549	5.64E-07	549	20	13528	11.08852	0.002021	5.48E-06	0.001036
GOTERM_BP_FAT	GO:0032374~regulation of cholesterol transport	9	1.624549	5.64E-07	549	20	13528	11.08852	0.002021	5.48E-06	0.001036
GOTERM_BP_FAT	GO:0034367~macromolecular complex remodeling	9	1.624549	5.64E-07	549	20	13528	11.08852	0.002021	5.48E-06	0.001036
GOTERM_BP_FAT	GO:0034369~plasma lipoprotein particle remodeling	9	1.624549	5.64E-07	549	20	13528	11.08852	0.002021	5.48E-06	0.001036
GOTERM_BP_FAT	GO:0034368~protein-lipid complex remodeling	9	1.624549	5.64E-07	549	20	13528	11.08852	0.002021	5.48E-06	0.001036
GOTERM_BP_FAT	GO:0030301~cholesterol transport	11	1.98556	1.42E-06	549	37	13528	7.325752	0.005082	1.32E-05	0.002609
GOTERM_BP_FAT	GO:0015918~sterol transport	11	1.98556	1.42E-06	549	37	13528	7.325752	0.005082	1.32E-05	0.002609
GOTERM_BP_FAT	GO:0055092~sterol homeostasis	11	1.98556	1.86E-06	549	38	13528	7.132969	0.006644	1.68E-05	0.003413

GOTERM_BP_FAT	GO:0042632~cholesterol homeostasis	11	1.98556	1.86E-06	549	38	13528	7.132969	0.006644	1.68E-05	0.003413
GOTERM_BP_FAT	GO:0006638~neutral lipid metabolic process	12	2.166065	3.87E-06	549	50	13528	5.91388	0.01378	3.36E-05	0.007104
GOTERM_BP_FAT	GO:0033344~cholesterol efflux	8	1.444043	5.67E-06	549	19	13528	10.37523	0.020149	4.77E-05	0.010421
GOTERM_BP_FAT	GO:0006641~triglyceride metabolic process	11	1.98556	6.28E-06	549	43	13528	6.303554	0.02227	5.23E-05	0.01153
GOTERM_BP_FAT	GO:0006639~acylglycerol metabolic process	11	1.98556	2.16E-05	549	49	13528	5.53169	0.074622	1.67E-04	0.039699
GOTERM_BP_FAT	GO:0033700~phospholipid efflux	6	1.083032	2.28E-05	549	10	13528	14.7847	0.078497	1.76E-04	0.041846
GOTERM_BP_FAT	GO:0043691~reverse cholesterol transport	7	1.263538	2.43E-05	549	16	13528	10.78051	0.083548	1.86E-04	0.044659
GOTERM_BP_FAT	GO:0006662~glycerol ether metabolic process	11	1.98556	3.13E-05	549	51	13528	5.314761	0.106056	2.37E-04	0.057384
GOTERM_BP_FAT	GO:0018904~organic ether metabolic process	11	1.98556	4.43E-05	549	53	13528	5.114204	0.147065	3.27E-04	0.08141
GOTERM_BP_FAT	GO:0034377~plasma lipoprotein particle assembly	6	1.083032	6.69E-05	549	12	13528	12.32058	0.213459	4.83E-04	0.122858
GOTERM_BP_FAT	GO:0065005~protein-lipid complex assembly	6	1.083032	6.69E-05	549	12	13528	12.32058	0.213459	4.83E-04	0.122858
GOTERM_CC_FAT	GO:0042627~chylomicron	6	1.083032	6.76E-05	520	12	12782	12.29038	0.025967	5.37E-04	0.093909
GOTERM_BP_FAT	GO:0019218~regulation of steroid metabolic process	9	1.624549	7.64E-05	549	36	13528	6.160291	0.239579	5.44E-04	0.140126
GOTERM_CC_FAT	GO:0034385~triglyceride-rich lipoprotein particle	7	1.263538	1.04E-04	520	20	12782	8.603269	0.039535	7.75E-04	0.143946
GOTERM_CC_FAT	GO:0034361~very-low-density lipoprotein particle	7	1.263538	1.04E-04	520	20	12782	8.603269	0.039535	7.75E-04	0.143946
GOTERM_BP_FAT	GO:0032488~Cdc42 protein signal transduction	4	0.722022	2.57E-04	549	4	13528	24.64117	0.601555	0.001684	0.470016
GOTERM_BP_FAT	GO:0006642~triglyceride mobilization	4	0.722022	2.57E-04	549	4	13528	24.64117	0.601555	0.001684	0.470016
GOTERM_CC_FAT	GO:0034363~intermediate-density lipoprotein particle	4	0.722022	2.58E-04	520	4	12782	24.58077	0.095581	0.001761	0.358115
GOTERM_CC_FAT	GO:0032994~protein-lipid complex	8	1.444043	4.36E-04	520	35	12782	5.618462	0.15615	0.002873	0.604461
GOTERM_CC_FAT	GO:0034358~plasma lipoprotein particle	8	1.444043	4.36E-04	520	35	12782	5.618462	0.15615	0.002873	0.604461
GOTERM_BP_FAT	GO:0007266~Rho protein signal transduction	8	1.444043	7.28E-04	549	38	13528	5.187614	0.926612	0.004417	1.328409
GOTERM_MF_FAT	GO:0005496~steroid binding	10	1.805054	9.83E-04	523	64	12983	3.878764	0.535068	0.008665	1.492593
GOTERM_MF_FAT	GO:0017127~cholesterol transporter activity	5	0.902527	9.88E-04	523	12	12983	10.34337	0.537019	0.008615	1.500728
GOTERM_BP_FAT	GO:0008203~cholesterol metabolic process	12	2.166065	0.001199	549	92	13528	3.214065	0.986496	0.006998	2.179894
GOTERM_BP_FAT	GO:0042159~lipoprotein catabolic process	4	0.722022	0.001206	549	6	13528	16.42744	0.986831	0.007027	2.192472
GOTERM_BP_FAT	GO:0034384~high-density lipoprotein particle clearance	4	0.722022	0.001206	549	6	13528	16.42744	0.986831	0.007027	2.192472
GOTERM_BP_FAT	GO:0042157~lipoprotein metabolic process	11	1.98556	0.001408	549	80	13528	3.38816	0.993622	0.008107	2.554846
GOTERM_BP_FAT	GO:0015914~phospholipid transport	7	1.263538	0.001871	549	33	13528	5.226914	0.998792	0.010558	3.381562
GOTERM_MF_FAT	GO:0015248~sterol transporter activity	5	0.902527	0.001874	523	14	12983	8.865747	0.76803	0.015589	2.828334

GOTERM_BP_FAT	GO:0045940~positive regulation of steroid metabolic process	5	0.902527	0.001927	549	14	13528	8.800416	0.999011	0.01082	3.480288
GOTERM_BP_FAT	GO:0034372~very-low-density lipoprotein particle remodeling	4	0.722022	0.002048	549	7	13528	14.08067	0.999359	0.011441	3.694588
GOTERM_BP_FAT	GO:0010873~positive regulation of cholesterol esterification	4	0.722022	0.002048	549	7	13528	14.08067	0.999359	0.011441	3.694588
GOTERM_MF_FAT	GO:0070325~lipoprotein receptor binding	5	0.902527	0.002475	523	15	12983	8.274697	0.854896	0.019907	3.719395
GOTERM_BP_FAT	GO:0042304~regulation of fatty acid biosynthetic process	5	0.902527	0.002544	549	15	13528	8.213722	0.999892	0.014003	4.570779
GOTERM_BP_FAT	GO:0016125~sterol metabolic process	12	2.166065	0.002559	549	101	13528	2.927663	0.999898	0.014064	4.597335
GOTERM_BP_FAT	GO:0070328~triglyceride homeostasis	4	0.722022	0.003178	549	8	13528	12.32058	0.999989	0.017178	5.678468
GOTERM_BP_FAT	GO:0010872~regulation of cholesterol esterification	4	0.722022	0.003178	549	8	13528	12.32058	0.999989	0.017178	5.678468
GOTERM_MF_FAT	GO:0015485~cholesterol binding	5	0.902527	0.004048	523	17	12983	7.301203	0.957571	0.030213	6.016224
GOTERM_BP_FAT	GO:0051004~regulation of lipoprotein lipase activity	5	0.902527	0.00416	549	17	13528	7.247402	1	0.022102	7.369469
GOTERM_BP_FAT	GO:0034370~triglyceride-rich lipoprotein particle remodeling	4	0.722022	0.004625	549	9	13528	10.95163	1	0.024368	8.160688
GOTERM_BP_FAT	GO:0034371~chylomicron remodeling	3	0.541516	0.004782	549	3	13528	24.64117	1	0.025151	8.426804
GOTERM_MF_FAT	GO:0032934~sterol binding	5	0.902527	0.006186	523	19	12983	6.532656	0.992044	0.042614	9.055222
GOTERM_BP_FAT	GO:0034375~high-density lipoprotein particle remodeling	4	0.722022	0.011062	549	12	13528	8.213722	1	0.054129	18.47696
GOTERM_CC_FAT	GO:0034362~low-density lipoprotein particle	4	0.722022	0.01404	520	13	12782	7.563314	0.995914	0.064119	17.83292
GOTERM_MF_FAT	GO:0060228~phosphatidylcholine-sterol O-acyltransferase activator activity	3	0.541516	0.014881	523	5	12983	14.89446	0.999992	0.085927	20.49442
GOTERM_BP_FAT	GO:0034380~high-density lipoprotein particle assembly	3	0.541516	0.015098	549	5	13528	14.7847	1	0.071837	24.37517
GOTERM_BP_FAT	GO:0034382~chylomicron remnant clearance	3	0.541516	0.015098	549	5	13528	14.7847	1	0.071837	24.37517
GOTERM_BP_FAT	GO:0010901~regulation of very-low-density lipoprotein particle remodeling	3	0.541516	0.015098	549	5	13528	14.7847	1	0.071837	24.37517
GOTERM_CC_FAT	GO:0034364~high-density lipoprotein particle	5	0.902527	0.017252	520	25	12782	4.916154	0.998852	0.077431	21.47501
GOTERM_BP_FAT	GO:0030300~regulation of intestinal cholesterol absorption	3	0.541516	0.022043	549	6	13528	12.32058	1	0.100612	33.59233
GOTERM_BP_FAT	GO:0010896~regulation of triglyceride catabolic process	3	0.541516	0.022043	549	6	13528	12.32058	1	0.100612	33.59233
GOTERM_BP_FAT	GO:0032375~negative regulation of cholesterol transport	3	0.541516	0.030042	549	7	13528	10.5605	1	0.13214	42.88908
GOTERM_BP_FAT	GO:0032372~negative regulation of sterol transport	3	0.541516	0.030042	549	7	13528	10.5605	1	0.13214	42.88908
GOTERM_BP_FAT	GO:0044242~cellular lipid catabolic process	8	1.444043	0.033952	549	76	13528	2.593807	1	0.147048	46.97267
GOTERM_BP_FAT	GO:0019433~triglyceride catabolic process	3	0.541516	0.038996	549	8	13528	9.240437	1	0.16639	51.83347
GOTERM_CC_FAT	GO:0034366~spherical high-density lipoprotein particle	3	0.541516	0.039163	520	8	12782	9.217788	1	0.149457	42.59067
GOTERM_BP_FAT	GO:0046464~acylglycerol catabolic process	3	0.541516	0.048817	549	9	13528	8.213722	1	0.201909	60.11391

GOTERM_BP_FAT	GO:0046461~neutral lipid catabolic process	3	0.541516	0.048817	549	9	13528	8.213722	1	0.201909	60.11391
GOTERM_BP_FAT	GO:0044269~glycerol ether catabolic process	3	0.541516	0.048817	549	9	13528	8.213722	1	0.201909	60.11391
GOTERM_BP_FAT	GO:0034374~low-density lipoprotein particle remodeling	3	0.541516	0.048817	549	9	13528	8.213722	1	0.201909	60.11391
GOTERM_BP_FAT	GO:0046503~glycerolipid catabolic process	3	0.541516	0.082655	549	12	13528	6.160291	1	0.31498	79.49239
GOTERM_MF_FAT	GO:0005319~lipid transporter activity	6	1.083032	0.108635	523	63	12983	2.364199	1	0.415176	82.78016
GOTERM_BP_FAT	GO:0008202~steroid metabolic process	13	2.34657	0.119406	549	202	13528	1.585818	1	0.421261	90.32159
GOTERM_CC_FAT	GO:0005788~endoplasmic reticulum lumen	5	0.902527	0.409491	520	80	12782	1.536298	1	0.763794	99.93362
GOTERM_BP_FAT	GO:0042158~lipoprotein biosynthetic process	4	0.722022	0.419111	549	58	13528	1.699391	1	0.879435	99.99535
Annotation Cluster 106	Enrichment Score: 3.3174161198947405										
Category	Term	Count	%	PValue	List Total	Pop Hits	Pop Total	Fold Enrichment	Bonferroni	Benjamini	FDR
GOTERM_MF_FAT	GO:0010843~promoter binding	14	2.527076	2.98E-07	523	57	12983	6.097145	2.32E-04	5.96E-06	4.56E-04
GOTERM_MF_FAT	GO:0003690~double-stranded DNA binding	11	1.98556	0.005542	523	97	12983	2.815103	0.98682	0.038938	8.149253
GOTERM_MF_FAT	GO:0043566~structure-specific DNA binding	11	1.98556	0.067523	523	145	12983	1.883207	1	0.2885	65.67819
Annotation Cluster 107	Enrichment Score: 3.313785640020695										
Category	Term	Count	%	PValue	List Total	Pop Hits	Pop Total	Fold Enrichment	Bonferroni	Benjamini	FDR
GOTERM_BP_FAT	GO:0033280~response to vitamin D	8	1.444043	1.43E-06	549	16	13528	12.32058	0.005127	1.33E-05	0.002632
GOTERM_BP_FAT	GO:0001890~placenta development	11	1.98556	1.33E-04	549	60	13528	4.517547	0.379288	9.20E-04	0.243863
GOTERM_BP_FAT	GO:0046697~decidualization	4	0.722022	0.013954	549	13	13528	7.581897	1	0.066807	22.74595
GOTERM_BP_FAT	GO:0001893~maternal placenta development	4	0.722022	0.020907	549	15	13528	6.570978	1	0.09599	32.16016
Annotation Cluster 108	Enrichment Score: 3.305173240086562										
Category	Term	Count	%	PValue	List Total	Pop Hits	Pop Total	Fold Enrichment	Bonferroni	Benjamini	FDR
GOTERM_CC_FAT	GO:0030139~endocytic vesicle	15	2.707581	6.87E-08	520	59	12782	6.249348	2.67E-05	7.86E-07	9.54E-05
GOTERM_CC_FAT	GO:0012506~vesicle membrane	15	2.707581	0.003353	520	151	12782	2.441798	0.729267	0.018493	4.558845
GOTERM_CC_FAT	GO:0030666~endocytic vesicle membrane	6	1.083032	0.010026	520	33	12782	4.469231	0.980156	0.048408	13.06252
GOTERM_CC_FAT	GO:0030659~cytoplasmic vesicle membrane	12	2.166065	0.026059	520	139	12782	2.122081	0.999965	0.110164	30.70457
Annotation Cluster 109	Enrichment Score: 3.2848110398658537										
Category	Term	Count	%	PValue	List Total	Pop Hits	Pop Total	Fold Enrichment	Bonferroni	Benjamini	FDR
GOTERM_BP_FAT	GO:0010869~regulation of receptor biosynthetic process	6	1.083032	1.18E-05	549	9	13528	16.42744	0.041402	9.48E-05	0.021646

GOTERM_BP_FAT	GO:0010871~negative regulation of receptor biosynthetic process	5	0.902527	1.29E-05	549	5	13528	24.64117	0.045197	1.02E-04	0.023677
GOTERM_BP_FAT	GO:0010745~negative regulation of foam cell differentiation	6	1.083032	6.69E-05	549	12	13528	12.32058	0.213459	4.83E-04	0.122858
GOTERM_BP_FAT	GO:0010888~negative regulation of lipid storage	5	0.902527	0.001017	549	12	13528	10.26715	0.973961	0.005982	1.850479
GOTERM_BP_FAT	GO:0045715~negative regulation of low-density lipoprotein receptor biosynthetic process	3	0.541516	0.004782	549	3	13528	24.64117	1	0.025151	8.426804
GOTERM_BP_FAT	GO:0045714~regulation of low-density lipoprotein receptor biosynthetic process	3	0.541516	0.009307	549	4	13528	18.48087	1	0.046271	15.77949
GOTERM_BP_FAT	GO:0010887~negative regulation of cholesterol storage	3	0.541516	0.022043	549	6	13528	12.32058	1	0.100612	33.59233

Annotation Cluster 110 Enrichment Score: 3.269709181818027

Category	Term	Count	%	PValue	List Total	Pop Hits	Pop Total	Fold Enrichment	Bonferroni	Benjamini	FDR
GOTERM_BP_FAT	GO:0051952~regulation of amine transport	14	2.527076	1.87E-10	549	33	13528	10.45383	6.71E-07	2.84E-09	3.43E-07
GOTERM_BP_FAT	GO:0050433~regulation of catecholamine secretion	10	1.805054	1.77E-08	549	19	13528	12.96903	6.34E-05	2.05E-07	3.25E-05
GOTERM_BP_FAT	GO:0032309~icosanoid secretion	8	1.444043	4.94E-08	549	11	13528	17.92085	1.77E-04	5.40E-07	9.07E-05
GOTERM_BP_FAT	GO:0050482~arachidonic acid secretion	8	1.444043	4.94E-08	549	11	13528	17.92085	1.77E-04	5.40E-07	9.07E-05
GOTERM_BP_FAT	GO:0051954~positive regulation of amine transport	8	1.444043	4.61E-07	549	14	13528	14.08067	0.001654	4.52E-06	8.48E-04
GOTERM_BP_FAT	GO:0042220~response to cocaine	8	1.444043	5.67E-06	549	19	13528	10.37523	0.020149	4.77E-05	0.010421
GOTERM_BP_FAT	GO:0014073~response to tropane	8	1.444043	5.67E-06	549	19	13528	10.37523	0.020149	4.77E-05	0.010421
GOTERM_BP_FAT	GO:0051580~regulation of neurotransmitter uptake	5	0.902527	3.74E-05	549	6	13528	20.5343	0.125666	2.81E-04	0.068733
GOTERM_BP_FAT	GO:0051590~positive regulation of neurotransmitter transport	5	0.902527	3.74E-05	549	6	13528	20.5343	0.125666	2.81E-04	0.068733
GOTERM_BP_FAT	GO:0032409~regulation of transporter activity	9	1.624549	3.90E-05	549	33	13528	6.720318	0.130539	2.91E-04	0.071592
GOTERM_BP_FAT	GO:0014059~regulation of dopamine secretion	6	1.083032	4.04E-05	549	11	13528	13.44064	0.134876	3.00E-04	0.074151
GOTERM_BP_FAT	GO:0051588~regulation of neurotransmitter transport	8	1.444043	5.78E-05	549	26	13528	7.581897	0.187378	4.21E-04	0.106176
GOTERM_BP_FAT	GO:0001975~response to amphetamine	7	1.263538	7.43E-05	549	19	13528	9.078324	0.233861	5.32E-04	0.136297
GOTERM_BP_FAT	GO:0034762~regulation of transmembrane transport	8	1.444043	7.54E-05	549	27	13528	7.301086	0.236932	5.39E-04	0.13835
GOTERM_BP_FAT	GO:0032410~negative regulation of transporter activity	6	1.083032	1.58E-04	549	14	13528	10.5605	0.432952	0.001084	0.290035
GOTERM_BP_FAT	GO:0048148~behavioral response to cocaine	5	0.902527	1.64E-04	549	8	13528	15.40073	0.44411	0.00112	0.30018
GOTERM_BP_FAT	GO:0007195~inhibition of adenylate cyclase activity by dopamine receptor signaling pathway	4	0.722022	2.57E-04	549	4	13528	24.64117	0.601555	0.001684	0.470016
GOTERM_BP_FAT	GO:0050709~negative regulation of protein secretion	6	1.083032	3.23E-04	549	16	13528	9.240437	0.685617	0.002087	0.590693
GOTERM_BP_FAT	GO:0034765~regulation of ion transmembrane transport	7	1.263538	3.94E-04	549	25	13528	6.899526	0.756678	0.002521	0.721017
GOTERM_BP_FAT	GO:0032225~regulation of synaptic transmission, dopaminergic	5	0.902527	4.60E-04	549	10	13528	12.32058	0.808125	0.002897	0.841685

GOTERM_BP_FAT	GO:0022898~regulation of transmembrane transporter activity	7	1.263538	4.95E-04	549	26	13528	6.63416	0.830553	0.003099	0.90477
GOTERM_BP_FAT	GO:0006576~biogenic amine metabolic process	13	2.34657	5.34E-04	549	97	13528	3.302424	0.852913	0.003322	0.976545
GOTERM_BP_FAT	GO:0051940~regulation of catecholamine uptake during transmission of nerve impulse	4	0.722022	6.22E-04	549	5	13528	19.71293	0.892632	0.003814	1.135995
GOTERM_BP_FAT	GO:0051584~regulation of dopamine uptake	4	0.722022	6.22E-04	549	5	13528	19.71293	0.892632	0.003814	1.135995
GOTERM_BP_FAT	GO:0051224~negative regulation of protein transport	8	1.444043	0.001003	549	40	13528	4.928233	0.972632	0.005911	1.825455
GOTERM_BP_FAT	GO:0032412~regulation of ion transmembrane transporter activity	6	1.083032	0.0024	549	24	13528	6.160291	0.999819	0.013253	4.31674
GOTERM_BP_FAT	GO:0032413~negative regulation of ion transmembrane transporter activity	4	0.722022	0.003178	549	8	13528	12.32058	0.999989	0.017178	5.678468
GOTERM_BP_FAT	GO:0001964~startle response	5	0.902527	0.003285	549	16	13528	7.700364	0.999993	0.017697	5.863647
GOTERM_BP_FAT	GO:0051925~regulation of calcium ion transport via voltage-gated calcium channel activity	4	0.722022	0.004625	549	9	13528	10.95163	1	0.024368	8.160688
GOTERM_MF_FAT	GO:0001591~dopamine receptor activity, coupled via Gi/Go	3	0.541516	0.004711	523	3	12983	24.82409	0.97475	0.033798	6.969135
GOTERM_BP_FAT	GO:0034776~response to histamine	3	0.541516	0.004782	549	3	13528	24.64117	1	0.025151	8.426804
GOTERM_BP_FAT	GO:0007212~dopamine receptor signaling pathway	5	0.902527	0.005179	549	18	13528	6.844768	1	0.027019	9.095605
GOTERM_BP_FAT	GO:0008344~adult locomotory behavior	8	1.444043	0.005852	549	54	13528	3.650543	1	0.03018	10.21819
GOTERM_BP_FAT	GO:0042417~dopamine metabolic process	5	0.902527	0.007691	549	20	13528	6.160291	1	0.038956	13.2203
GOTERM_BP_FAT	GO:0032415~regulation of sodium:hydrogen antiporter activity	3	0.541516	0.009307	549	4	13528	18.48087	1	0.046271	15.77949
GOTERM_BP_FAT	GO:0051927~negative regulation of calcium ion transport via voltage-gated calcium channel activity	3	0.541516	0.009307	549	4	13528	18.48087	1	0.046271	15.77949
GOTERM_BP_FAT	GO:0001956~positive regulation of neurotransmitter secretion	3	0.541516	0.009307	549	4	13528	18.48087	1	0.046271	15.77949
GOTERM_BP_FAT	GO:0034311~diol metabolic process	6	1.083032	0.011271	549	34	13528	4.348441	1	0.055055	18.79305
GOTERM_BP_FAT	GO:0006584~catecholamine metabolic process	6	1.083032	0.011271	549	34	13528	4.348441	1	0.055055	18.79305
GOTERM_BP_FAT	GO:0009712~catechol metabolic process	6	1.083032	0.011271	549	34	13528	4.348441	1	0.055055	18.79305
GOTERM_BP_FAT	GO:0018958~phenol metabolic process	6	1.083032	0.012723	549	35	13528	4.2242	1	0.061386	20.955
GOTERM_MF_FAT	GO:0004952~dopamine receptor activity	3	0.541516	0.014881	523	5	12983	14.89446	0.999992	0.085927	20.49442
GOTERM_BP_FAT	GO:0006575~cellular amino acid derivative metabolic process	14	2.527076	0.017514	549	166	13528	2.078171	1	0.081766	27.71113
GOTERM_BP_FAT	GO:0010155~regulation of proton transport	3	0.541516	0.030042	549	7	13528	10.5605	1	0.13214	42.88908
GOTERM_BP_FAT	GO:0007270~nerve-nerve synaptic transmission	5	0.902527	0.035241	549	31	13528	3.974382	1	0.151917	48.25705
GOTERM_MF_FAT	GO:0035240~dopamine binding	3	0.541516	0.048157	523	9	12983	8.274697	1	0.219677	52.9965
GOTERM_BP_FAT	GO:0046928~regulation of neurotransmitter secretion	4	0.722022	0.051127	549	21	13528	4.693555	1	0.21014	61.85627
GOTERM_BP_FAT	GO:0060134~prepulse inhibition	3	0.541516	0.059419	549	10	13528	7.39235	1	0.238358	67.53433

GOTERM_MF_FAT	GO:0043178~alcohol binding	4	0.722022	0.077234	523	25	12983	3.971855	1	0.318967	70.75652
GOTERM_BP_FAT	GO:0050707~regulation of cytokine secretion	3	0.541516	0.344529	549	30	13528	2.464117	1	0.812542	99.95724
Annotation Cluster 111	Enrichment Score: 3.246989578787373										
Category	Term	Count	%	PValue	List Total	Pop Hits	Pop Total	Fold Enrichment	Bonferroni	Benjamini	FDR
GOTERM_BP_FAT	GO:0051056~regulation of small GTPase mediated signal transduction	24	4.33213	2.40E-04	549	252	13528	2.346778	0.577704	0.001589	0.440387
GOTERM_BP_FAT	GO:0046578~regulation of Ras protein signal transduction	21	3.790614	3.46E-04	549	210	13528	2.464117	0.711259	0.002232	0.633987
GOTERM_BP_FAT	GO:0035023~regulation of Rho protein signal transduction	12	2.166065	0.002182	549	99	13528	2.986808	0.999605	0.01215	3.932527
Annotation Cluster 112	Enrichment Score: 3.229877738095089										
Category	Term	Count	%	PValue	List Total	Pop Hits	Pop Total	Fold Enrichment	Bonferroni	Benjamini	FDR
GOTERM_BP_FAT	GO:0006351~transcription, DNA-dependent	26	4.693141	3.51E-04	549	292	13528	2.194076	0.716052	0.002258	0.642502
GOTERM_BP_FAT	GO:0032774~RNA biosynthetic process	26	4.693141	4.30E-04	549	296	13528	2.164427	0.785917	0.002724	0.786069
GOTERM_BP_FAT	GO:0006366~transcription from RNA polymerase II promoter	21	3.790614	0.001355	549	234	13528	2.211387	0.99229	0.007829	2.460147
Annotation Cluster 113	Enrichment Score: 3.1952374641943684										
Category	Term	Count	%	PValue	List Total	Pop Hits	Pop Total	Fold Enrichment	Bonferroni	Benjamini	FDR
GOTERM_BP_FAT	GO:0042063~gliogenesis	12	2.166065	5.35E-05	549	65	13528	4.549138	0.17449	3.90E-04	0.098128
GOTERM_BP_FAT	GO:0010001~glial cell differentiation	10	1.805054	2.48E-04	549	53	13528	4.649277	0.588752	0.001629	0.453898
GOTERM_BP_FAT	GO:0021782~glial cell development	5	0.902527	0.019606	549	26	13528	4.738686	1	0.0907	30.48577
Annotation Cluster 114	Enrichment Score: 3.155687111606034										
Category	Term	Count	%	PValue	List Total	Pop Hits	Pop Total	Fold Enrichment	Bonferroni	Benjamini	FDR
GOTERM_BP_FAT	GO:0045927~positive regulation of growth	13	2.34657	8.42E-05	549	80	13528	4.004189	0.260818	5.99E-04	0.154609
GOTERM_BP_FAT	GO:0045793~positive regulation of cell size	9	1.624549	6.21E-04	549	48	13528	4.620219	0.892312	0.003815	1.13449
GOTERM_BP_FAT	GO:0030307~positive regulation of cell growth	7	1.263538	0.00652	549	42	13528	4.106861	1	0.033385	11.3192
Annotation Cluster 115	Enrichment Score: 3.151448740802228										
Category	Term	Count	%	PValue	List Total	Pop Hits	Pop Total	Fold Enrichment	Bonferroni	Benjamini	FDR
GOTERM_BP_FAT	GO:0043933~macromolecular complex subunit organization	54	9.747292	1.18E-05	549	710	13528	1.874117	0.041377	9.50E-05	0.021633
GOTERM_BP_FAT	GO:0006461~protein complex assembly	42	7.581227	1.78E-05	549	505	13528	2.049364	0.061965	1.39E-04	0.032745
GOTERM_BP_FAT	GO:0070271~protein complex biogenesis	42	7.581227	1.78E-05	549	505	13528	2.049364	0.061965	1.39E-04	0.032745
GOTERM_BP_FAT	GO:0065003~macromolecular complex assembly	49	8.844765	7.09E-05	549	665	13528	1.815665	0.224634	5.10E-04	0.130175

GOTERM_BP_FAT	GO:0007172~signal complex assembly	6	1.083032	3.23E-04	549	16	13528	9.240437	0.685617	0.002087	0.590693
GOTERM_BP_FAT	GO:0043623~cellular protein complex assembly	15	2.707581	0.006155	549	162	13528	2.281589	1	0.031674	10.71867
GOTERM_BP_FAT	GO:0034622~cellular macromolecular complex assembly	16	2.888087	0.30955	549	318	13528	1.239807	1	0.773394	99.88891
GOTERM_BP_FAT	GO:0034621~cellular macromolecular complex subunit organization	17	3.068592	0.376249	549	357	13528	1.173389	1	0.843137	99.98281

Annotation Cluster 116	Enrichment Score: 3.1120447073933626										
Category	Term	Count	%	PValue	List Total	Pop Hits	Pop Total	Fold Enrichment	Bonferroni	Benjamini	FDR
GOTERM_BP_FAT	GO:0009895~negative regulation of catabolic process	13	2.34657	1.27E-08	549	37	13528	8.657707	4.55E-05	1.52E-07	2.33E-05
GOTERM_BP_FAT	GO:0042176~regulation of protein catabolic process	11	1.98556	3.73E-05	549	52	13528	5.212554	0.125272	2.81E-04	0.068502
GOTERM_BP_FAT	GO:0030162~regulation of proteolysis	10	1.805054	2.48E-04	549	53	13528	4.649277	0.588752	0.001629	0.453898
GOTERM_BP_FAT	GO:0042177~negative regulation of protein catabolic process	5	0.902527	0.00416	549	17	13528	7.247402	1	0.022102	7.369469
GOTERM_BP_FAT	GO:0051043~regulation of membrane protein ectodomain proteolysis	4	0.722022	0.013954	549	13	13528	7.581897	1	0.066807	22.74595
GOTERM_BP_FAT	GO:0045862~positive regulation of proteolysis	5	0.902527	0.017124	549	25	13528	4.928233	1	0.08042	27.18208
GOTERM_BP_FAT	GO:0045732~positive regulation of protein catabolic process	5	0.902527	0.022298	549	27	13528	4.563179	1	0.101598	33.9094
GOTERM_BP_FAT	GO:0051044~positive regulation of membrane protein ectodomain proteolysis	3	0.541516	0.048817	549	9	13528	8.213722	1	0.201909	60.11391

Annotation Cluster 117	Enrichment Score: 3.093703785142868										
Category	Term	Count	%	PValue	List Total	Pop Hits	Pop Total	Fold Enrichment	Bonferroni	Benjamini	FDR
GOTERM_BP_FAT	GO:0033135~regulation of peptidyl-serine phosphorylation	6	1.083032	1.58E-04	549	14	13528	10.5605	0.432952	0.001084	0.290035
GOTERM_BP_FAT	GO:0001776~leukocyte homeostasis	8	1.444043	5.16E-04	549	36	13528	5.475815	0.843218	0.003217	0.944176
GOTERM_BP_FAT	GO:0033138~positive regulation of peptidyl-serine phosphorylation	4	0.722022	0.00641	549	10	13528	9.856466	1	0.032875	11.13836

Annotation Cluster 118	Enrichment Score: 3.0122275084151466										
Category	Term	Count	%	PValue	List Total	Pop Hits	Pop Total	Fold Enrichment	Bonferroni	Benjamini	FDR
GOTERM_BP_FAT	GO:0046903~secretion	37	6.6787	5.02E-09	549	300	13528	3.039077	1.80E-05	6.30E-08	9.22E-06
GOTERM_BP_FAT	GO:0003001~generation of a signal involved in cell-cell signaling	13	2.34657	1.53E-04	549	85	13528	3.768649	0.422586	0.001054	0.280787
GOTERM_BP_FAT	GO:0046879~hormone secretion	9	1.624549	3.95E-04	549	45	13528	4.928233	0.75731	0.002521	0.722338
GOTERM_BP_FAT	GO:0009914~hormone transport	9	1.624549	7.16E-04	549	49	13528	4.525928	0.923513	0.004362	1.307511
GOTERM_BP_FAT	GO:0032940~secretion by cell	20	3.610108	7.60E-04	549	207	13528	2.380789	0.934639	0.004582	1.386906
GOTERM_BP_FAT	GO:0002790~peptide secretion	7	1.263538	0.005782	549	41	13528	4.207028	1	0.029907	10.10138
GOTERM_BP_FAT	GO:0030072~peptide hormone secretion	6	1.083032	0.019782	549	39	13528	3.790949	1	0.091369	30.71515

GOTERM_BP_FAT	GO:0010817~regulation of hormone levels	13	2.34657	0.019787	549	151	13528	2.121425	1	0.091272	30.72092
GOTERM_BP_FAT	GO:0015833~peptide transport	7	1.263538	0.021398	549	54	13528	3.194225	1	0.097907	32.78265
GOTERM_BP_FAT	GO:0030073~insulin secretion	4	0.722022	0.094311	549	27	13528	3.650543	1	0.350965	83.7848
Annotation Cluster 119	Enrichment Score: 2.9800942094851646										
Category	Term	Count	%	PValue	List Total	Pop Hits	Pop Total	Fold Enrichment	Bonferroni	Benjamini	FDR
GOTERM_BP_FAT	GO:0048872~homeostasis of number of cells	15	2.707581	4.85E-05	549	100	13528	3.696175	0.159542	3.56E-04	0.088948
GOTERM_BP_FAT	GO:0001776~leukocyte homeostasis	8	1.444043	5.16E-04	549	36	13528	5.475815	0.843218	0.003217	0.944176
GOTERM_BP_FAT	GO:0002260~lymphocyte homeostasis	7	1.263538	7.56E-04	549	28	13528	6.160291	0.933593	0.004563	1.378892
GOTERM_BP_FAT	GO:0043029~T cell homeostasis	6	1.083032	0.00127	549	21	13528	7.040333	0.989531	0.007374	2.307272
GOTERM_BP_FAT	GO:0070227~lymphocyte apoptosis	4	0.722022	0.00641	549	10	13528	9.856466	1	0.032875	11.13836
GOTERM_BP_FAT	GO:0001782~B cell homeostasis	4	0.722022	0.008551	549	11	13528	8.960424	1	0.04287	14.58998
Annotation Cluster 120	Enrichment Score: 2.9598648256328017										
Category	Term	Count	%	PValue	List Total	Pop Hits	Pop Total	Fold Enrichment	Bonferroni	Benjamini	FDR
GOTERM_BP_FAT	GO:0032890~regulation of organic acid transport	7	1.263538	3.63E-05	549	17	13528	10.14636	0.122111	2.74E-04	0.066657
GOTERM_BP_FAT	GO:0032891~negative regulation of organic acid transport	5	0.902527	4.60E-04	549	10	13528	12.32058	0.808125	0.002897	0.841685
GOTERM_BP_FAT	GO:0010748~negative regulation of plasma membrane long-chain fatty acid transport	3	0.541516	0.009307	549	4	13528	18.48087	1	0.046271	15.77949
GOTERM_BP_FAT	GO:0010746~regulation of plasma membrane long-chain fatty acid transport	3	0.541516	0.009307	549	4	13528	18.48087	1	0.046271	15.77949
Annotation Cluster 121	Enrichment Score: 2.8942472985510315										
Category	Term	Count	%	PValue	List Total	Pop Hits	Pop Total	Fold Enrichment	Bonferroni	Benjamini	FDR
GOTERM_BP_FAT	GO:0043277~apoptotic cell clearance	5	0.902527	3.74E-05	549	6	13528	20.5343	0.125666	2.81E-04	0.068733
GOTERM_MF_FAT	GO:0008034~lipoprotein binding	9	1.624549	5.83E-05	523	35	12983	6.383338	0.04439	7.32E-04	0.089118
GOTERM_MF_FAT	GO:0030228~lipoprotein receptor activity	5	0.902527	0.002475	523	15	12983	8.274697	0.854896	0.019907	3.719395
GOTERM_MF_FAT	GO:0030169~low-density lipoprotein binding	5	0.902527	0.008964	523	21	12983	5.910498	0.999101	0.05868	12.8676
GOTERM_MF_FAT	GO:0005041~low-density lipoprotein receptor activity	3	0.541516	0.069794	523	11	12983	6.770207	1	0.295355	66.93448
Annotation Cluster 122	Enrichment Score: 2.814571221140054										
Category	Term	Count	%	PValue	List Total	Pop Hits	Pop Total	Fold Enrichment	Bonferroni	Benjamini	FDR
GOTERM_BP_FAT	GO:0032429~regulation of phospholipase A2 activity	4	0.722022	6.22E-04	549	5	13528	19.71293	0.892632	0.003814	1.135995
GOTERM_BP_FAT	GO:0032430~positive regulation of phospholipase A2 activity	4	0.722022	6.22E-04	549	5	13528	19.71293	0.892632	0.003814	1.135995

GOTERM_BP_FAT	GO:0032431~activation of phospholipase A2 activity	3	0.541516	0.009307	549	4	13528	18.48087	1	0.046271	15.77949

Annotation Cluster 123	Enrichment Score: 2.7514006334529606										
Category	Term	Count	%	PValue	List Total	Pop Hits	Pop Total	Fold Enrichment	Bonferroni	Benjamini	FDR
GOTERM_BP_FAT	GO:0003014~renal system process	9	1.624549	1.04E-05	549	28	13528	7.920375	0.03676	8.47E-05	0.019173
GOTERM_BP_FAT	GO:0030147~natriuresis	5	0.902527	8.46E-05	549	7	13528	17.60083	0.261624	6.00E-04	0.155166
GOTERM_BP_FAT	GO:0030146~diuresis	4	0.722022	0.003178	549	8	13528	12.32058	0.999989	0.017178	5.678468
GOTERM_BP_FAT	GO:0030104~water homeostasis	5	0.902527	0.003285	549	16	13528	7.700364	0.999993	0.017697	5.863647
GOTERM_BP_FAT	GO:0003091~renal water homeostasis	4	0.722022	0.00641	549	10	13528	9.856466	1	0.032875	11.13836
GOTERM_BP_FAT	GO:0050891~multicellular organismal water homeostasis	4	0.722022	0.008551	549	11	13528	8.960424	1	0.04287	14.58998
GOTERM_BP_FAT	GO:0007588~excretion	8	1.444043	0.008652	549	58	13528	3.398781	1	0.043311	14.75082
GOTERM_BP_FAT	GO:0007589~body fluid secretion	5	0.902527	0.022298	549	27	13528	4.563179	1	0.101598	33.9094

Annotation Cluster 124	Enrichment Score: 2.746806917962088										
Category	Term	Count	%	PValue	List Total	Pop Hits	Pop Total	Fold Enrichment	Bonferroni	Benjamini	FDR
GOTERM_BP_FAT	GO:0001936~regulation of endothelial cell proliferation	9	1.624549	3.06E-05	549	32	13528	6.930328	0.104013	2.33E-04	0.056216
GOTERM_BP_FAT	GO:0001938~positive regulation of endothelial cell proliferation	6	1.083032	2.29E-04	549	15	13528	9.856466	0.560785	0.001525	0.420361
GOTERM_BP_FAT	GO:0030949~positive regulation of vascular endothelial growth factor receptor signaling pathway	3	0.541516	0.030042	549	7	13528	10.5605	1	0.13214	42.88908
GOTERM_BP_FAT	GO:0030947~regulation of vascular endothelial growth factor receptor signaling pathway	3	0.541516	0.048817	549	9	13528	8.213722	1	0.201909	60.11391

Annotation Cluster 125	Enrichment Score: 2.721119216512646										
Category	Term	Count	%	PValue	List Total	Pop Hits	Pop Total	Fold Enrichment	Bonferroni	Benjamini	FDR
GOTERM_BP_FAT	GO:0010741~negative regulation of protein kinase cascade	9	1.624549	7.64E-05	549	36	13528	6.160291	0.239579	5.44E-04	0.140126
GOTERM_BP_FAT	GO:0043409~negative regulation of MAPKKK cascade	5	0.902527	0.00416	549	17	13528	7.247402	1	0.022102	7.369469
GOTERM_BP_FAT	GO:0046329~negative regulation of JNK cascade	4	0.722022	0.00641	549	10	13528	9.856466	1	0.032875	11.13836
GOTERM_BP_FAT	GO:0070303~negative regulation of stress-activated protein kinase signaling pathway	4	0.722022	0.00641	549	10	13528	9.856466	1	0.032875	11.13836

Annotation Cluster 126	Enrichment Score: 2.70807407801857										
Category	Term	Count	%	PValue	List Total	Pop Hits	Pop Total	Fold Enrichment	Bonferroni	Benjamini	FDR
GOTERM_BP_FAT	GO:0001701~in utero embryonic development	20	3.610108	9.53E-05	549	176	13528	2.800132	0.289489	6.70E-04	0.17483
GOTERM_BP_FAT	GO:0043009~chordate embryonic development	24	4.33213	0.008438	549	331	13528	1.786671	1	0.042373	14.41123

GOTERM_BP_FAT	GO:0009792~embryonic development ending in birth or egg hatching	24	4.33213	0.009345	549	334	13528	1.770623	1	0.046326	15.83783
Annotation Cluster 127	Enrichment Score: 2.7028166133316787										
Category	Term	Count	%	PValue	List Total	Pop Hits	Pop Total	Fold Enrichment	Bonferroni	Benjamini	FDR
GOTERM_BP_FAT	GO:0015758~glucose transport	7	1.263538	9.21E-04	549	29	13528	5.947868	0.963268	0.005492	1.677434
GOTERM_BP_FAT	GO:0008645~hexose transport	7	1.263538	0.001112	549	30	13528	5.749605	0.981519	0.006511	2.022618
GOTERM_BP_FAT	GO:0015749~monosaccharide transport	7	1.263538	0.001332	549	31	13528	5.564134	0.991624	0.007709	2.418786
GOTERM_BP_FAT	GO:0008643~carbohydrate transport	8	1.444043	0.011321	549	61	13528	3.231628	1	0.055217	18.86804
Annotation Cluster 128	Enrichment Score: 2.677255776084738										
Category	Term	Count	%	PValue	List Total	Pop Hits	Pop Total	Fold Enrichment	Bonferroni	Benjamini	FDR
GOTERM_BP_FAT	GO:0032095~regulation of response to food	5	0.902527	1.64E-04	549	8	13528	15.40073	0.44411	0.00112	0.30018
GOTERM_BP_FAT	GO:0032107~regulation of response to nutrient levels	5	0.902527	0.001422	549	13	13528	9.477371	0.993921	0.008157	2.578776
GOTERM_BP_FAT	GO:0032104~regulation of response to extracellular stimulus	5	0.902527	0.001422	549	13	13528	9.477371	0.993921	0.008157	2.578776
GOTERM_BP_FAT	GO:0032108~negative regulation of response to nutrient levels	4	0.722022	0.002048	549	7	13528	14.08067	0.999359	0.011441	3.694588
GOTERM_BP_FAT	GO:0032105~negative regulation of response to extracellular stimulus	4	0.722022	0.002048	549	7	13528	14.08067	0.999359	0.011441	3.694588
GOTERM_BP_FAT	GO:0032098~regulation of appetite	4	0.722022	0.003178	549	8	13528	12.32058	0.999989	0.017178	5.678468
GOTERM_BP_FAT	GO:0032099~negative regulation of appetite	3	0.541516	0.009307	549	4	13528	18.48087	1	0.046271	15.77949
GOTERM_BP_FAT	GO:0032096~negative regulation of response to food	3	0.541516	0.009307	549	4	13528	18.48087	1	0.046271	15.77949
Annotation Cluster 129	Enrichment Score: 2.6720906902875727										
Category	Term	Count	%	PValue	List Total	Pop Hits	Pop Total	Fold Enrichment	Bonferroni	Benjamini	FDR
GOTERM_BP_FAT	GO:0051348~negative regulation of transferase activity	13	2.34657	4.86E-04	549	96	13528	3.336825	0.824866	0.003046	0.888021
GOTERM_BP_FAT	GO:0006469~negative regulation of protein kinase activity	12	2.166065	7.49E-04	549	87	13528	3.398781	0.932052	0.004532	1.367313
GOTERM_BP_FAT	GO:0033673~negative regulation of kinase activity	12	2.166065	9.98E-04	549	90	13528	3.285489	0.972196	0.005904	1.817523
GOTERM_BP_FAT	GO:0043407~negative regulation of MAP kinase activity	5	0.902527	0.056419	549	36	13528	3.422384	1	0.228745	65.57949
Annotation Cluster 130	Enrichment Score: 2.667902813653496										
Category	Term	Count	%	PValue	List Total	Pop Hits	Pop Total	Fold Enrichment	Bonferroni	Benjamini	FDR
GOTERM_BP_FAT	GO:0002763~positive regulation of myeloid leukocyte differentiation	7	1.263538	5.26E-05	549	18	13528	9.582676	0.17196	3.86E-04	0.096563
GOTERM_BP_FAT	GO:0045649~regulation of macrophage differentiation	4	0.722022	0.008551	549	11	13528	8.960424	1	0.04287	14.58998
GOTERM_BP_FAT	GO:0045651~positive regulation of macrophage differentiation	3	0.541516	0.022043	549	6	13528	12.32058	1	0.100612	33.59233

Annotation Cluster 131	Enrichment Score: 2.6313559839848133										
Category	Term	Count	%	PValue	List Total	Pop Hits	Pop Total	Fold Enrichment	Bonferroni	Benjamini	FDR
GOTERM_BP_FAT	GO:0048511~rhythmic process	26	4.693141	4.55E-11	549	128	13528	5.005237	1.63E-07	7.29E-10	8.36E-08
GOTERM_BP_FAT	GO:0042698~ovulation cycle	13	2.34657	1.35E-05	549	67	13528	4.781122	0.0474	1.07E-04	0.024859
GOTERM_BP_FAT	GO:0003006~reproductive developmental process	25	4.512635	1.67E-04	549	262	13528	2.351256	0.451437	0.001143	0.306953
GOTERM_BP_FAT	GO:0022602~ovulation cycle process	11	1.98556	1.76E-04	549	62	13528	4.37182	0.468727	0.001193	0.323299
GOTERM_BP_FAT	GO:0001894~tissue homeostasis	10	1.805054	9.24E-04	549	63	13528	3.911296	0.963699	0.005502	1.683374
GOTERM_BP_FAT	GO:0045137~development of primary sexual characteristics	14	2.527076	0.001911	549	127	13528	2.716349	0.998953	0.010765	3.452293
GOTERM_BP_FAT	GO:0008406~gonad development	13	2.34657	0.001914	549	112	13528	2.860135	0.998963	0.010763	3.456874
GOTERM_BP_FAT	GO:0008585~female gonad development	9	1.624549	0.004139	549	64	13528	3.465164	1	0.022027	7.33477
GOTERM_BP_FAT	GO:0048608~reproductive structure development	13	2.34657	0.005071	549	126	13528	2.542342	1	0.026539	8.914197
GOTERM_BP_FAT	GO:0046660~female sex differentiation	9	1.624549	0.006561	549	69	13528	3.214065	1	0.033543	11.38613
GOTERM_BP_FAT	GO:0046545~development of primary female sexual characteristics	9	1.624549	0.006561	549	69	13528	3.214065	1	0.033543	11.38613
GOTERM_BP_FAT	GO:0007548~sex differentiation	14	2.527076	0.008383	549	151	13528	2.284611	1	0.042164	14.32526
GOTERM_BP_FAT	GO:0046546~development of primary male sexual characteristics	8	1.444043	0.015745	549	65	13528	3.032759	1	0.074428	25.28196
GOTERM_BP_FAT	GO:0030728~ovulation	4	0.722022	0.017234	549	14	13528	7.040333	1	0.080606	27.33166
GOTERM_BP_FAT	GO:0008584~male gonad development	7	1.263538	0.018041	549	52	13528	3.31708	1	0.083923	28.41914
GOTERM_BP_FAT	GO:0001541~ovarian follicle development	6	1.083032	0.024115	549	41	13528	3.606024	1	0.109227	36.12819
GOTERM_BP_FAT	GO:0046661~male sex differentiation	8	1.444043	0.028026	549	73	13528	2.700402	1	0.124633	40.66929
GOTERM_BP_FAT	GO:0043129~surfactant homeostasis	3	0.541516	0.059419	549	10	13528	7.39235	1	0.238358	67.53433
GOTERM_BP_FAT	GO:0048875~chemical homeostasis within a tissue	3	0.541516	0.059419	549	10	13528	7.39235	1	0.238358	67.53433
GOTERM_BP_FAT	GO:0001542~ovulation from ovarian follicle	3	0.541516	0.082655	549	12	13528	6.160291	1	0.31498	79.49239
GOTERM_BP_FAT	GO:0007292~female gamete generation	6	1.083032	0.122689	549	65	13528	2.274569	1	0.428571	90.9633

Annotation Cluster 132	Enrichment Score: 2.596144838333753										
Category	Term	Count	%	PValue	List Total	Pop Hits	Pop Total	Fold Enrichment	Bonferroni	Benjamini	FDR
GOTERM_BP_FAT	GO:0009743~response to carbohydrate stimulus	10	1.805054	6.43E-04	549	60	13528	4.106861	0.900323	0.003927	1.173611
GOTERM_BP_FAT	GO:0034284~response to monosaccharide stimulus	8	1.444043	0.00265	549	47	13528	4.194241	0.999926	0.01449	4.755725
GOTERM_BP_FAT	GO:0009746~response to hexose stimulus	8	1.444043	0.00265	549	47	13528	4.194241	0.999926	0.01449	4.755725

GOTERM_BP_FAT	GO:0009749~response to glucose stimulus	7	1.263538	0.009143	549	45	13528	3.83307	1	0.045595	15.52253

Annotation Cluster 133	Enrichment Score: 2.582259606953184										
Category	Term	Count	%	PValue	List Total	Pop Hits	Pop Total	Fold Enrichment	Bonferroni	Benjamini	FDR
GOTERM_BP_FAT	GO:0043255~regulation of carbohydrate biosynthetic process	9	1.624549	8.79E-07	549	21	13528	10.5605	0.003148	8.30E-06	0.001614
GOTERM_BP_FAT	GO:0006006~glucose metabolic process	14	2.527076	0.009317	549	153	13528	2.254747	1	0.046252	15.79387
GOTERM_BP_FAT	GO:0019318~hexose metabolic process	14	2.527076	0.048728	549	192	13528	1.796752	1	0.201799	60.04539
GOTERM_BP_FAT	GO:0005996~monosaccharide metabolic process	14	2.527076	0.117485	549	222	13528	1.553947	1	0.416189	89.92643

Annotation Cluster 134	Enrichment Score: 2.5452318356555748										
Category	Term	Count	%	PValue	List Total	Pop Hits	Pop Total	Fold Enrichment	Bonferroni	Benjamini	FDR
GOTERM_BP_FAT	GO:0050871~positive regulation of B cell activation	10	1.805054	4.44E-06	549	33	13528	7.46702	0.015793	3.80E-05	0.00815
GOTERM_BP_FAT	GO:0002819~regulation of adaptive immune response	12	2.166065	1.24E-05	549	56	13528	5.28025	0.0434	9.88E-05	0.022714
GOTERM_BP_FAT	GO:0002697~regulation of immune effector process	16	2.888087	1.27E-05	549	101	13528	3.903551	0.044657	1.01E-04	0.023387
GOTERM_BP_FAT	GO:0002822~regulation of adaptive immune response based on somatic recombination of immune receptors built from immunoglobulin superfamily domains	11	1.98556	6.18E-05	549	55	13528	4.928233	0.198974	4.48E-04	0.113526
GOTERM_BP_FAT	GO:0002700~regulation of production of molecular mediator of immune response	9	1.624549	2.02E-04	549	41	13528	5.409036	0.514994	0.001357	0.369786
GOTERM_BP_FAT	GO:0002703~regulation of leukocyte mediated immunity	10	1.805054	7.27E-04	549	61	13528	4.039535	0.92645	0.004421	1.327293
GOTERM_BP_FAT	GO:0050714~positive regulation of protein secretion	8	1.444043	0.001168	549	41	13528	4.808032	0.984862	0.006824	2.122645
GOTERM_BP_FAT	GO:0002706~regulation of lymphocyte mediated immunity	9	1.624549	0.001385	549	54	13528	4.106861	0.993056	0.007984	2.51239
GOTERM_BP_FAT	GO:0002889~regulation of immunoglobulin mediated immune response	6	1.083032	0.0024	549	24	13528	6.160291	0.999819	0.013253	4.31674
GOTERM_BP_FAT	GO:0002712~regulation of B cell mediated immunity	6	1.083032	0.0024	549	24	13528	6.160291	0.999819	0.013253	4.31674
GOTERM_BP_FAT	GO:0002637~regulation of immunoglobulin production	6	1.083032	0.003474	549	26	13528	5.686423	0.999996	0.018682	6.191788
GOTERM_BP_FAT	GO:0045911~positive regulation of DNA recombination	4	0.722022	0.004625	549	9	13528	10.95163	1	0.024368	8.160688
GOTERM_BP_FAT	GO:0045830~positive regulation of isotype switching	4	0.722022	0.004625	549	9	13528	10.95163	1	0.024368	8.160688
GOTERM_BP_FAT	GO:0002821~positive regulation of adaptive immune response	6	1.083032	0.0076	549	31	13528	4.769258	1	0.038557	13.07425
GOTERM_BP_FAT	GO:0002699~positive regulation of immune effector process	7	1.263538	0.008197	549	44	13528	3.920185	1	0.041359	14.02934
GOTERM_BP_FAT	GO:0045191~regulation of isotype switching	4	0.722022	0.013954	549	13	13528	7.581897	1	0.066807	22.74595
GOTERM_BP_FAT	GO:0002824~positive regulation of adaptive immune response based on somatic recombination of immune receptors built from immunoglobulin superfamily domains	5	0.902527	0.031675	549	30	13528	4.106861	1	0.138243	44.62906
GOTERM_BP_FAT	GO:0002705~positive regulation of leukocyte mediated immunity	5	0.902527	0.04728	549	34	13528	3.623701	1	0.196745	58.9136

GOTERM_BP_FAT	GO:0002708~positive regulation of lymphocyte mediated immunity	5	0.902527	0.04728	549	34	13528	3.623701	1	0.196745	58.9136
GOTERM_BP_FAT	GO:0051023~regulation of immunoglobulin secretion	3	0.541516	0.048817	549	9	13528	8.213722	1	0.201909	60.11391
GOTERM_BP_FAT	GO:0000018~regulation of DNA recombination	4	0.722022	0.086309	549	26	13528	3.790949	1	0.326541	80.94206
GOTERM_BP_FAT	GO:0002711~positive regulation of T cell mediated immunity	3	0.541516	0.12155	549	15	13528	4.928233	1	0.426152	90.74532
GOTERM_BP_FAT	GO:0002709~regulation of T cell mediated immunity	3	0.541516	0.20828	549	21	13528	3.520167	1	0.618644	98.62828

Annotation Cluster 135 Enrichment Score: 2.5396165243281583

Category	Term	Count	%	PValue	List Total	Pop Hits	Pop Total	Fold Enrichment	Bonferroni	Benjamini	FDR
GOTERM_BP_FAT	GO:0045727~positive regulation of translation	7	1.263538	2.41E-04	549	23	13528	7.499485	0.578159	0.001585	0.440936
GOTERM_BP_FAT	GO:0010608~posttranscriptional regulation of gene expression	20	3.610108	9.58E-04	549	211	13528	2.335656	0.967816	0.005682	1.743954
GOTERM_BP_FAT	GO:0006417~regulation of translation	10	1.805054	0.104402	549	137	13528	1.798625	1	0.379777	86.80058

Annotation Cluster 136 Enrichment Score: 2.510678990423444

Category	Term	Count	%	PValue	List Total	Pop Hits	Pop Total	Fold Enrichment	Bonferroni	Benjamini	FDR
GOTERM_BP_FAT	GO:0030818~negative regulation of cAMP biosynthetic process	4	0.722022	0.002048	549	7	13528	14.08067	0.999359	0.011441	3.694588
GOTERM_BP_FAT	GO:0030815~negative regulation of cAMP metabolic process	4	0.722022	0.002048	549	7	13528	14.08067	0.999359	0.011441	3.694588
GOTERM_BP_FAT	GO:0030809~negative regulation of nucleotide biosynthetic process	4	0.722022	0.003178	549	8	13528	12.32058	0.999989	0.017178	5.678468
GOTERM_BP_FAT	GO:0030803~negative regulation of cyclic nucleotide biosynthetic process	4	0.722022	0.003178	549	8	13528	12.32058	0.999989	0.017178	5.678468
GOTERM_BP_FAT	GO:0030800~negative regulation of cyclic nucleotide metabolic process	4	0.722022	0.003178	549	8	13528	12.32058	0.999989	0.017178	5.678468
GOTERM_BP_FAT	GO:0045980~negative regulation of nucleotide metabolic process	4	0.722022	0.00641	549	10	13528	9.856466	1	0.032875	11.13836

Annotation Cluster 137 Enrichment Score: 2.510292558618309

Category	Term	Count	%	PValue	List Total	Pop Hits	Pop Total	Fold Enrichment	Bonferroni	Benjamini	FDR
GOTERM_BP_FAT	GO:0022407~regulation of cell-cell adhesion	7	1.263538	1.84E-04	549	22	13528	7.840371	0.483341	0.001241	0.337532
GOTERM_BP_FAT	GO:0070587~regulation of cell-cell adhesion involved in gastrulation	3	0.541516	0.004782	549	3	13528	24.64117	1	0.025151	8.426804
GOTERM_BP_FAT	GO:0034114~regulation of heterotypic cell-cell adhesion	3	0.541516	0.004782	549	3	13528	24.64117	1	0.025151	8.426804
GOTERM_BP_FAT	GO:0010470~regulation of gastrulation	3	0.541516	0.004782	549	3	13528	24.64117	1	0.025151	8.426804
GOTERM_BP_FAT	GO:0045995~regulation of embryonic development	4	0.722022	0.013954	549	13	13528	7.581897	1	0.066807	22.74595

Annotation Cluster 138 Enrichment Score: 2.5012593407011257

Category	Term	Count	%	PValue	List Total	Pop Hits	Pop Total	Fold Enrichment	Bonferroni	Benjamini	FDR
GOTERM_BP_FAT	GO:0060537~muscle tissue development	14	2.527076	0.001656	549	125	13528	2.759811	0.997376	0.009389	2.99703

GOTERM_BP_FAT	GO:0007517~muscle organ development	19	3.429603	0.002357	549	211	13528	2.218873	0.999789	0.013037	4.240876
GOTERM_BP_FAT	GO:0014706~striated muscle tissue development	13	2.34657	0.003187	549	119	13528	2.691892	0.999989	0.017197	5.693039
GOTERM_BP_FAT	GO:0007519~skeletal muscle tissue development	9	1.624549	0.005007	549	66	13528	3.360159	1	0.026244	8.805976
GOTERM_BP_FAT	GO:0060538~skeletal muscle organ development	9	1.624549	0.005007	549	66	13528	3.360159	1	0.026244	8.805976
Annotation Cluster 139	Enrichment Score: 2.4920580125458245										
Category	Term	Count	%	PValue	List Total	Pop Hits	Pop Total	Fold Enrichment	Bonferroni	Benjamini	FDR
GOTERM_BP_FAT	GO:0031668~cellular response to extracellular stimulus	11	1.98556	2.31E-04	549	64	13528	4.2352	0.563391	0.001533	0.423394
GOTERM_BP_FAT	GO:0042594~response to starvation	7	1.263538	0.00652	549	42	13528	4.106861	1	0.033385	11.3192
GOTERM_BP_FAT	GO:0031669~cellular response to nutrient levels	7	1.263538	0.008197	549	44	13528	3.920185	1	0.041359	14.02934
GOTERM_BP_FAT	GO:0009267~cellular response to starvation	6	1.083032	0.008715	549	32	13528	4.620219	1	0.043556	14.84899
Annotation Cluster 140	Enrichment Score: 2.485745910535345										
Category	Term	Count	%	PValue	List Total	Pop Hits	Pop Total	Fold Enrichment	Bonferroni	Benjamini	FDR
GOTERM_MF_FAT	GO:0004936~alpha-adrenergic receptor activity	4	0.722022	0.00118	523	6	12983	16.54939	0.601425	0.010057	1.790017
GOTERM_MF_FAT	GO:0004938~alpha2-adrenergic receptor activity	3	0.541516	0.004711	523	3	12983	24.82409	0.97475	0.033798	6.969135
GOTERM_MF_FAT	GO:0004935~adrenoceptor activity	4	0.722022	0.006276	523	10	12983	9.929637	0.992586	0.042844	9.180968
Annotation Cluster 141	Enrichment Score: 2.4843159001985886										
Category	Term	Count	%	PValue	List Total	Pop Hits	Pop Total	Fold Enrichment	Bonferroni	Benjamini	FDR
GOTERM_BP_FAT	GO:0051705~behavioral interaction between organisms	10	1.805054	7.56E-06	549	35	13528	7.040333	0.026743	6.22E-05	0.013877
GOTERM_BP_FAT	GO:0007618~mating	6	1.083032	0.004124	549	27	13528	5.475815	1	0.02198	7.309068
GOTERM_BP_FAT	GO:0019098~reproductive behavior	4	0.722022	0.034284	549	18	13528	5.475815	1	0.148221	47.30622
GOTERM_BP_FAT	GO:0007617~mating behavior	3	0.541516	0.108131	549	14	13528	5.28025	1	0.390159	87.77417
Annotation Cluster 142	Enrichment Score: 2.4840307981042127										
Category	Term	Count	%	PValue	List Total	Pop Hits	Pop Total	Fold Enrichment	Bonferroni	Benjamini	FDR
GOTERM_BP_FAT	GO:0045777~positive regulation of blood pressure	8	1.444043	2.40E-05	549	23	13528	8.57084	0.082376	1.84E-04	0.044005
GOTERM_BP_FAT	GO:0032230~positive regulation of synaptic transmission, GABAergic	4	0.722022	0.001206	549	6	13528	16.42744	0.986831	0.007027	2.192472
GOTERM_BP_FAT	GO:0003078~regulation of natriuresis	4	0.722022	0.003178	549	8	13528	12.32058	0.999989	0.017178	5.678468
GOTERM_BP_FAT	GO:0007618~mating	6	1.083032	0.004124	549	27	13528	5.475815	1	0.02198	7.309068
GOTERM_BP_FAT	GO:0044062~regulation of excretion	4	0.722022	0.004625	549	9	13528	10.95163	1	0.024368	8.160688

GOTERM_BP_FAT	GO:0007320~insemination	4	0.722022	0.008551	549	11	13528	8.960424	1	0.04287	14.58998
GOTERM_BP_FAT	GO:0003079~positive regulation of natriuresis	3	0.541516	0.009307	549	4	13528	18.48087	1	0.046271	15.77949
GOTERM_BP_FAT	GO:0042713~sperm ejaculation	3	0.541516	0.015098	549	5	13528	14.7847	1	0.071837	24.37517
GOTERM_BP_FAT	GO:0007620~copulation	4	0.722022	0.020907	549	15	13528	6.570978	1	0.09599	32.16016

Annotation Cluster 143 Enrichment Score: 2.4802843788801896

Category	Term	Count	%	PValue	List Total	Pop Hits	Pop Total	Fold Enrichment	Bonferroni	Benjamini	FDR
GOTERM_BP_FAT	GO:0045737~positive regulation of cyclin-dependent protein kinase activity	5	0.902527	1.64E-04	549	8	13528	15.40073	0.44411	0.00112	0.30018
GOTERM_BP_FAT	GO:0070141~response to UV-A	3	0.541516	0.004782	549	3	13528	24.64117	1	0.025151	8.426804
GOTERM_BP_FAT	GO:0000079~regulation of cyclin-dependent protein kinase activity	8	1.444043	0.005852	549	54	13528	3.650543	1	0.03018	10.21819
GOTERM_BP_FAT	GO:0031659~positive regulation of cyclin-dependent protein kinase activity during G1/S	3	0.541516	0.009307	549	4	13528	18.48087	1	0.046271	15.77949
GOTERM_BP_FAT	GO:0031657~regulation of cyclin-dependent protein kinase activity during G1/S	3	0.541516	0.009307	549	4	13528	18.48087	1	0.046271	15.77949

Annotation Cluster 144 Enrichment Score: 2.4753803658019957

Category	Term	Count	%	PValue	List Total	Pop Hits	Pop Total	Fold Enrichment	Bonferroni	Benjamini	FDR
GOTERM_BP_FAT	GO:0001763~morphogenesis of a branching structure	13	2.34657	3.83E-05	549	74	13528	4.328853	0.12827	2.87E-04	0.070259
GOTERM_BP_FAT	GO:0048754~branching morphogenesis of a tube	11	1.98556	2.63E-04	549	65	13528	4.170043	0.611141	0.001725	0.482426
GOTERM_BP_FAT	GO:0035295~tube development	19	3.429603	0.003696	549	220	13528	2.128101	0.999998	0.019802	6.573387
GOTERM_BP_FAT	GO:0035239~tube morphogenesis	13	2.34657	0.005401	549	127	13528	2.522324	1	0.028081	9.466987
GOTERM_BP_FAT	GO:0001569~patterning of blood vessels	5	0.902527	0.009202	549	21	13528	5.866944	1	0.045821	15.615
GOTERM_BP_FAT	GO:0007389~pattern specification process	10	1.805054	0.759284	549	267	13528	0.92289	1	0.994582	100

Annotation Cluster 145 Enrichment Score: 2.456837991204766

Category	Term	Count	%	PValue	List Total	Pop Hits	Pop Total	Fold Enrichment	Bonferroni	Benjamini	FDR
GOTERM_BP_FAT	GO:0050853~B cell receptor signaling pathway	5	0.902527	2.85E-04	549	9	13528	13.68954	0.640544	0.001862	0.522478
GOTERM_BP_FAT	GO:0050778~positive regulation of immune response	16	2.888087	7.94E-04	549	145	13528	2.719025	0.942104	0.004769	1.448118
GOTERM_BP_FAT	GO:0002757~immune response-activating signal transduction	9	1.624549	0.001075	549	52	13528	4.264817	0.978881	0.006304	1.955662
GOTERM_BP_FAT	GO:0002764~immune response-regulating signal transduction	9	1.624549	0.001761	549	56	13528	3.960187	0.998206	0.009971	3.185494
GOTERM_BP_FAT	GO:0050851~antigen receptor-mediated signaling pathway	7	1.263538	0.001871	549	33	13528	5.226914	0.998792	0.010558	3.381562
GOTERM_BP_FAT	GO:0002429~immune response-activating cell surface receptor signaling pathway	7	1.263538	0.004491	549	39	13528	4.422773	1	0.023739	7.933583
GOTERM_BP_FAT	GO:0002768~immune response-regulating cell surface receptor	7	1.263538	0.00652	549	42	13528	4.106861	1	0.033385	11.3192

Category	Term	Count	%	PValue	List Total	Pop Hits	Pop Total	Fold Enrichment	Bonferroni	Benjamini	FDR
	signaling pathway										
GOTERM_BP_FAT	GO:0002253~activation of immune response	10	1.805054	0.013807	549	94	13528	2.621401	1	0.066209	22.53378
GOTERM_BP_FAT	GO:0050852~T cell receptor signaling pathway	3	0.541516	0.238519	549	23	13528	3.214065	1	0.671924	99.32909
Annotation Cluster 146	Enrichment Score: 2.4410254659324737										
Category	Term	Count	%	PValue	List Total	Pop Hits	Pop Total	Fold Enrichment	Bonferroni	Benjamini	FDR
GOTERM_BP_FAT	GO:0007213~muscarinic acetylcholine receptor signaling pathway	6	1.083032	4.04E-05	549	11	13528	13.44064	0.134876	3.00E-04	0.074151
GOTERM_CC_FAT	GO:0043679~nerve terminal	9	1.624549	9.54E-05	520	37	12782	5.979106	0.036416	7.27E-04	0.132383
GOTERM_CC_FAT	GO:0032279~asymmetric synapse	5	0.902527	2.88E-04	520	9	12782	13.65598	0.105872	0.001928	0.398826
GOTERM_MF_FAT	GO:0016907~G-protein coupled acetylcholine receptor activity	3	0.541516	0.014881	523	5	12983	14.89446	0.999992	0.085927	20.49442
GOTERM_MF_FAT	GO:0004981~muscarinic acetylcholine receptor activity	3	0.541516	0.014881	523	5	12983	14.89446	0.999992	0.085927	20.49442
GOTERM_MF_FAT	GO:0015464~acetylcholine receptor activity	3	0.541516	0.161932	523	18	12983	4.137349	1	0.540441	93.29424
GOTERM_MF_FAT	GO:0042166~acetylcholine binding	3	0.541516	0.205935	523	21	12983	3.546299	1	0.621284	97.06125
Annotation Cluster 147	Enrichment Score: 2.4108635663966966										
Category	Term	Count	%	PValue	List Total	Pop Hits	Pop Total	Fold Enrichment	Bonferroni	Benjamini	FDR
GOTERM_MF_FAT	GO:0060090~molecular adaptor activity	10	1.805054	0.001372	523	67	12983	3.705088	0.65672	0.011555	2.077607
GOTERM_MF_FAT	GO:0005070~SH3/SH2 adaptor activity	8	1.444043	0.003236	523	49	12983	4.052913	0.919928	0.025181	4.836846
GOTERM_MF_FAT	GO:0030674~protein binding, bridging	10	1.805054	0.013188	523	94	12983	2.640861	0.999968	0.08002	18.37841
Annotation Cluster 148	Enrichment Score: 2.312468036552906										
Category	Term	Count	%	PValue	List Total	Pop Hits	Pop Total	Fold Enrichment	Bonferroni	Benjamini	FDR
GOTERM_BP_FAT	GO:0044236~multicellular organismal metabolic process	9	1.624549	9.40E-05	549	37	13528	5.993797	0.286223	6.65E-04	0.172486
GOTERM_BP_FAT	GO:0044243~multicellular organismal catabolic process	7	1.263538	4.95E-04	549	26	13528	6.63416	0.830553	0.003099	0.90477
GOTERM_BP_FAT	GO:0032963~collagen metabolic process	7	1.263538	7.56E-04	549	28	13528	6.160291	0.933593	0.004563	1.378892
GOTERM_BP_FAT	GO:0044259~multicellular organismal macromolecule metabolic process	7	1.263538	0.001332	549	31	13528	5.564134	0.991624	0.007709	2.418786
GOTERM_BP_FAT	GO:0030574~collagen catabolic process	5	0.902527	0.007691	549	20	13528	6.160291	1	0.038956	13.2203
GOTERM_MF_FAT	GO:0004222~metalloendopeptidase activity	7	1.263538	0.240479	523	104	12983	1.670852	1	0.676247	98.51169
GOTERM_MF_FAT	GO:0008237~metallopeptidase activity	7	1.263538	0.750084	523	183	12983	0.949555	1	0.991422	100
Annotation Cluster 149	Enrichment Score: 2.2899831371036927										
Category	Term	Count	%	PValue	List Total	Pop Hits	Pop Total	Fold Enrichment	Bonferroni	Benjamini	FDR

GOTERM_BP_FAT	GO:0000060~protein import into nucleus, translocation	12	2.166065	5.34E-08	549	34	13528	8.696882	1.92E-04	5.79E-07	9.81E-05
GOTERM_BP_FAT	GO:0006606~protein import into nucleus	13	2.34657	1.72E-04	549	86	13528	3.724827	0.459492	0.001165	0.314503
GOTERM_BP_FAT	GO:0051170~nuclear import	13	2.34657	2.14E-04	549	88	13528	3.640172	0.536009	0.001429	0.392379
GOTERM_BP_FAT	GO:0034504~protein localization in nucleus	13	2.34657	3.99E-04	549	94	13528	3.407821	0.761417	0.002547	0.731014
GOTERM_BP_FAT	GO:0017038~protein import	15	2.707581	8.52E-04	549	131	13528	2.821508	0.953068	0.005094	1.554003
GOTERM_BP_FAT	GO:0033365~protein localization in organelle	15	2.707581	0.002417	549	146	13528	2.531627	0.99983	0.013325	4.346366
GOTERM_BP_FAT	GO:0006605~protein targeting	18	3.249097	0.006592	549	215	13528	2.062981	1	0.033604	11.43684
GOTERM_BP_FAT	GO:0006913~nucleocytoplasmic transport	13	2.34657	0.024811	549	156	13528	2.05343	1	0.111817	36.95997
GOTERM_BP_FAT	GO:0046907~intracellular transport	38	6.859206	0.026684	549	657	13528	1.425212	1	0.119251	39.1467
GOTERM_BP_FAT	GO:0051169~nuclear transport	13	2.34657	0.027062	549	158	13528	2.027438	1	0.120709	39.57967
GOTERM_BP_FAT	GO:0034613~cellular protein localization	26	4.693141	0.028197	549	411	13528	1.558809	1	0.125203	40.86128
GOTERM_BP_FAT	GO:0070727~cellular macromolecule localization	26	4.693141	0.030067	549	414	13528	1.547513	1	0.132087	42.91676
GOTERM_BP_FAT	GO:0006886~intracellular protein transport	22	3.971119	0.082188	549	374	13528	1.44948	1	0.313766	79.29994
GOTERM_BP_FAT	GO:0008104~protein localization	43	7.761733	0.151755	549	882	13528	1.201327	1	0.499074	95.13261
GOTERM_BP_FAT	GO:0045184~establishment of protein localization	35	6.31769	0.318386	549	769	13528	1.121509	1	0.784057	99.91231
GOTERM_BP_FAT	GO:0015031~protein transport	34	6.137184	0.357238	549	762	13528	1.099475	1	0.825533	99.97016
Annotation Cluster 150	Enrichment Score: 2.257233580096675										
Category	Term	Count	%	PValue	List Total	Pop Hits	Pop Total	Fold Enrichment	Bonferroni	Benjamini	FDR
GOTERM_BP_FAT	GO:0021954~central nervous system neuron development	7	1.263538	0.001585	549	32	13528	5.390255	0.996614	0.009045	2.870312
GOTERM_BP_FAT	GO:0021953~central nervous system neuron differentiation	7	1.263538	0.005106	549	40	13528	4.312204	1	0.026683	8.973471
GOTERM_BP_FAT	GO:0021955~central nervous system neuron axonogenesis	4	0.722022	0.020907	549	15	13528	6.570978	1	0.09599	32.16016
Annotation Cluster 151	Enrichment Score: 2.234480129997533										
Category	Term	Count	%	PValue	List Total	Pop Hits	Pop Total	Fold Enrichment	Bonferroni	Benjamini	FDR
GOTERM_BP_FAT	GO:0032845~negative regulation of homeostatic process	6	1.083032	0.00127	549	21	13528	7.040333	0.989531	0.007374	2.307272
GOTERM_BP_FAT	GO:0045779~negative regulation of bone resorption	4	0.722022	0.003178	549	8	13528	12.32058	0.999989	0.017178	5.678468
GOTERM_BP_FAT	GO:0046851~negative regulation of bone remodeling	4	0.722022	0.003178	549	8	13528	12.32058	0.999989	0.017178	5.678468
GOTERM_BP_FAT	GO:0034103~regulation of tissue remodeling	5	0.902527	0.006353	549	19	13528	6.484517	1	0.032637	11.04587
GOTERM_BP_FAT	GO:0034104~negative regulation of tissue remodeling	4	0.722022	0.00641	549	10	13528	9.856466	1	0.032875	11.13836

GOTERM_BP_FAT	GO:0045124~regulation of bone resorption	4	0.722022	0.020907	549	15	13528	6.570978	1	0.09599	32.16016
GOTERM_BP_FAT	GO:0046850~regulation of bone remodeling	4	0.722022	0.020907	549	15	13528	6.570978	1	0.09599	32.16016

Annotation Cluster 152	Enrichment Score: 2.2016447952561418										
Category	Term	Count	%	PValue	List Total	Pop Hits	Pop Total	Fold Enrichment	Bonferroni	Benjamini	FDR
GOTERM_MF_FAT	GO:0001530~lipopolysaccharide binding	5	0.902527	9.88E-04	523	12	12983	10.34337	0.537019	0.008615	1.500728
GOTERM_MF_FAT	GO:0001875~lipopolysaccharide receptor activity	3	0.541516	0.014881	523	5	12983	14.89446	0.999992	0.085927	20.49442
GOTERM_MF_FAT	GO:0008329~pattern recognition receptor activity	4	0.722022	0.016891	523	14	12983	7.092598	0.999998	0.094285	22.93883

Annotation Cluster 153	Enrichment Score: 2.1367514510957575										
Category	Term	Count	%	PValue	List Total	Pop Hits	Pop Total	Fold Enrichment	Bonferroni	Benjamini	FDR
GOTERM_BP_FAT	GO:0007179~transforming growth factor beta receptor signaling pathway	10	1.805054	6.43E-04	549	60	13528	4.106861	0.900323	0.003927	1.173611
GOTERM_MF_FAT	GO:0070412~R-SMAD binding	4	0.722022	0.006276	523	10	12983	9.929637	0.992586	0.042844	9.180968
GOTERM_MF_FAT	GO:0046332~SMAD binding	7	1.263538	0.009812	523	46	12983	3.777579	0.999538	0.062002	13.99996
GOTERM_BP_FAT	GO:0060395~SMAD protein signal transduction	3	0.541516	0.022043	549	6	13528	12.32058	1	0.100612	33.59233
GOTERM_BP_FAT	GO:0007178~transmembrane receptor protein serine/threonine kinase signaling pathway	10	1.805054	0.023746	549	103	13528	2.392346	1	0.107767	35.6839

Annotation Cluster 154	Enrichment Score: 2.1292764027299924										
Category	Term	Count	%	PValue	List Total	Pop Hits	Pop Total	Fold Enrichment	Bonferroni	Benjamini	FDR
GOTERM_BP_FAT	GO:0007044~cell-substrate junction assembly	6	1.083032	0.001964	549	23	13528	6.42813	0.999136	0.011012	3.546871
GOTERM_BP_FAT	GO:0034330~cell junction organization	9	1.624549	0.001978	549	57	13528	3.89071	0.999176	0.011068	3.570283
GOTERM_BP_FAT	GO:0034329~cell junction assembly	7	1.263538	0.005782	549	41	13528	4.207028	1	0.029907	10.10138
GOTERM_BP_FAT	GO:0048041~focal adhesion formation	3	0.541516	0.135347	549	16	13528	4.620219	1	0.461408	93.08008

Annotation Cluster 155	Enrichment Score: 2.118826628205427										
Category	Term	Count	%	PValue	List Total	Pop Hits	Pop Total	Fold Enrichment	Bonferroni	Benjamini	FDR
GOTERM_CC_FAT	GO:0014069~postsynaptic density	10	1.805054	0.002216	520	71	12782	3.46208	0.578029	0.012988	3.034199
GOTERM_CC_FAT	GO:0045202~synapse	27	4.873646	0.002705	520	355	12782	1.869523	0.651364	0.015155	3.693009
GOTERM_CC_FAT	GO:0044456~synapse part	19	3.429603	0.011589	520	246	12782	1.898515	0.989268	0.053798	14.94997
GOTERM_CC_FAT	GO:0045211~postsynaptic membrane	11	1.98556	0.048191	520	135	12782	2.002877	1	0.176399	49.64665

Annotation Cluster 156	Enrichment Score: 2.1128862999114766										
Category	Term	Count	%	PValue	List Total	Pop Hits	Pop Total	Fold	Bonferroni	Benjamini	FDR

Category	Term	Count	%	PValue	List Total	Pop Hits	Pop Total	Fold Enrichment	Bonferroni	Benjamini	FDR
GOTERM_MF_FAT	GO:0016455~RNA polymerase II transcription mediator activity	6	1.083032	0.005498	523	29	12983	5.136019	0.986361	0.038987	8.087526
GOTERM_CC_FAT	GO:0016592~Srb-mediator complex	6	1.083032	0.005729	520	29	12782	5.085676	0.893017	0.030154	7.671418
GOTERM_MF_FAT	GO:0016251~general RNA polymerase II transcription factor activity	7	1.263538	0.014556	523	50	12983	3.475373	0.999989	0.084737	20.09117

Annotation Cluster 157	Enrichment Score: 2.1014572327896173										
Category	Term	Count	%	PValue	List Total	Pop Hits	Pop Total	Fold Enrichment	Bonferroni	Benjamini	FDR
GOTERM_BP_FAT	GO:0045621~positive regulation of lymphocyte differentiation	8	1.444043	4.31E-04	549	35	13528	5.632266	0.786861	0.002727	0.788314
GOTERM_BP_FAT	GO:0045582~positive regulation of T cell differentiation	7	1.263538	0.001585	549	32	13528	5.390255	0.996614	0.009045	2.870312
GOTERM_BP_FAT	GO:0046634~regulation of alpha-beta T cell activation	7	1.263538	0.00297	549	36	13528	4.791338	0.999977	0.016108	5.316088
GOTERM_BP_FAT	GO:0045619~regulation of lymphocyte differentiation	9	1.624549	0.003752	549	63	13528	3.520167	0.999999	0.02004	6.669921
GOTERM_BP_FAT	GO:0045580~regulation of T cell differentiation	8	1.444043	0.004245	549	51	13528	3.865281	1	0.022483	7.514746
GOTERM_BP_FAT	GO:0046638~positive regulation of alpha-beta T cell differentiation	5	0.902527	0.007691	549	20	13528	6.160291	1	0.038956	13.2203
GOTERM_BP_FAT	GO:0043372~positive regulation of CD4-positive, alpha beta T cell differentiation	4	0.722022	0.008551	549	11	13528	8.960424	1	0.04287	14.58998
GOTERM_BP_FAT	GO:0046637~regulation of alpha-beta T cell differentiation	5	0.902527	0.017124	549	25	13528	4.928233	1	0.08042	27.18208
GOTERM_BP_FAT	GO:0046635~positive regulation of alpha-beta T cell activation	5	0.902527	0.019606	549	26	13528	4.738686	1	0.0907	30.48577
GOTERM_BP_FAT	GO:0043370~regulation of CD4-positive, alpha beta T cell differentiation	4	0.722022	0.020907	549	15	13528	6.570978	1	0.09599	32.16016
GOTERM_BP_FAT	GO:0045579~positive regulation of B cell differentiation	3	0.541516	0.030042	549	7	13528	10.5605	1	0.13214	42.88908
GOTERM_BP_FAT	GO:0045577~regulation of B cell differentiation	3	0.541516	0.135347	549	16	13528	4.620219	1	0.461408	93.08008

Annotation Cluster 158	Enrichment Score: 2.0993919581373426										
Category	Term	Count	%	PValue	List Total	Pop Hits	Pop Total	Fold Enrichment	Bonferroni	Benjamini	FDR
GOTERM_BP_FAT	GO:0048678~response to axon injury	7	1.263538	1.02E-04	549	20	13528	8.624408	0.307613	7.18E-04	0.188035
GOTERM_BP_FAT	GO:0031103~axon regeneration	3	0.541516	0.059419	549	10	13528	7.39235	1	0.238358	67.53433
GOTERM_BP_FAT	GO:0031102~neuron projection regeneration	3	0.541516	0.082655	549	12	13528	6.160291	1	0.31498	79.49239

Annotation Cluster 159	Enrichment Score: 2.094315109250816										
Category	Term	Count	%	PValue	List Total	Pop Hits	Pop Total	Fold Enrichment	Bonferroni	Benjamini	FDR
GOTERM_MF_FAT	GO:0046625~sphingolipid binding	4	0.722022	0.00118	523	6	12983	16.54939	0.601425	0.010057	1.790017
GOTERM_MF_FAT	GO:0043208~glycosphingolipid binding	3	0.541516	0.009172	523	4	12983	18.61807	0.999237	0.059018	13.14657
GOTERM_MF_FAT	GO:0051861~glycolipid binding	3	0.541516	0.048157	523	9	12983	8.274697	1	0.219677	52.9965

Annotation Cluster 160	Enrichment Score: 2.050941331119064										
Category	Term	Count	%	PValue	List Total	Pop Hits	Pop Total	Fold Enrichment	Bonferroni	Benjamini	FDR
GOTERM_BP_FAT	GO:0051092~positive regulation of NF-kappaB transcription factor activity	8	1.444043	0.001168	549	41	13528	4.808032	0.984862	0.006824	2.122645
GOTERM_BP_FAT	GO:0042035~regulation of cytokine biosynthetic process	10	1.805054	0.002913	549	74	13528	3.329887	0.999972	0.015827	5.217253
GOTERM_BP_FAT	GO:0032675~regulation of interleukin-6 production	7	1.263538	0.00297	549	36	13528	4.791338	0.999977	0.016108	5.316088
GOTERM_BP_FAT	GO:0032755~positive regulation of interleukin-6 production	5	0.902527	0.006353	549	19	13528	6.484517	1	0.032637	11.04587
GOTERM_BP_FAT	GO:0045073~regulation of chemokine biosynthetic process	4	0.722022	0.00641	549	10	13528	9.856466	1	0.032875	11.13836
GOTERM_BP_FAT	GO:0042108~positive regulation of cytokine biosynthetic process	7	1.263538	0.012445	549	48	13528	3.593503	1	0.060241	20.54584
GOTERM_BP_FAT	GO:0070304~positive regulation of stress-activated protein kinase signaling pathway	4	0.722022	0.051127	549	21	13528	4.693555	1	0.21014	61.85627
GOTERM_BP_FAT	GO:0046330~positive regulation of JNK cascade	3	0.541516	0.149469	549	17	13528	4.348441	1	0.494187	94.88599

Annotation Cluster 161	Enrichment Score: 1.9906266157165287										
Category	Term	Count	%	PValue	List Total	Pop Hits	Pop Total	Fold Enrichment	Bonferroni	Benjamini	FDR
GOTERM_BP_FAT	GO:0021799~cerebral cortex radially oriented cell migration	4	0.722022	0.003178	549	8	13528	12.32058	0.999989	0.017178	5.678468
GOTERM_BP_FAT	GO:0022029~telencephalon cell migration	5	0.902527	0.003285	549	16	13528	7.700364	0.999993	0.017697	5.863647
GOTERM_BP_FAT	GO:0021885~forebrain cell migration	5	0.902527	0.00416	549	17	13528	7.247402	1	0.022102	7.369469
GOTERM_BP_FAT	GO:0021537~telencephalon development	9	1.624549	0.004557	549	65	13528	3.411854	1	0.024051	8.046312
GOTERM_BP_FAT	GO:0021819~layer formation in the cerebral cortex	3	0.541516	0.004782	549	3	13528	24.64117	1	0.025151	8.426804
GOTERM_BP_FAT	GO:0021801~cerebral cortex radial glia guided migration	3	0.541516	0.009307	549	4	13528	18.48087	1	0.046271	15.77949
GOTERM_BP_FAT	GO:0021987~cerebral cortex development	6	1.083032	0.009937	549	33	13528	4.480212	1	0.048998	16.75623
GOTERM_BP_FAT	GO:0021795~cerebral cortex cell migration	4	0.722022	0.017234	549	14	13528	7.040333	1	0.080606	27.33166
GOTERM_BP_FAT	GO:0021543~pallium development	6	1.083032	0.037469	549	46	13528	3.214065	1	0.160498	50.40776
GOTERM_BP_FAT	GO:0021761~limbic system development	5	0.902527	0.039031	549	32	13528	3.850182	1	0.166333	51.865
GOTERM_BP_FAT	GO:0021766~hippocampus development	4	0.722022	0.05748	549	22	13528	4.480212	1	0.231858	66.28324

Annotation Cluster 162	Enrichment Score: 1.98770189974953										
Category	Term	Count	%	PValue	List Total	Pop Hits	Pop Total	Fold Enrichment	Bonferroni	Benjamini	FDR
GOTERM_BP_FAT	GO:0051346~negative regulation of hydrolase activity	9	1.624549	6.21E-04	549	48	13528	4.620219	0.892312	0.003815	1.13449
GOTERM_BP_FAT	GO:0043154~negative regulation of caspase activity	4	0.722022	0.034284	549	18	13528	5.475815	1	0.148221	47.30622
GOTERM_BP_FAT	GO:0010466~negative regulation of peptidase activity	4	0.722022	0.051127	549	21	13528	4.693555	1	0.21014	61.85627

Annotation Cluster 163	Enrichment Score: 1.9530480045247804										
Category	Term	Count	%	PValue	List Total	Pop Hits	Pop Total	Fold Enrichment	Bonferroni	Benjamini	FDR
GOTERM_BP_FAT	GO:0010469~regulation of receptor activity	5	0.902527	0.005179	549	18	13528	6.844768	1	0.027019	9.095605
GOTERM_BP_FAT	GO:0042058~regulation of epidermal growth factor receptor signaling pathway	5	0.902527	0.012774	549	23	13528	5.356775	1	0.061543	21.02986
GOTERM_BP_FAT	GO:0007176~regulation of epidermal growth factor receptor activity	4	0.722022	0.020907	549	15	13528	6.570978	1	0.09599	32.16016
Annotation Cluster 164	Enrichment Score: 1.9478834180161857										
Category	Term	Count	%	PValue	List Total	Pop Hits	Pop Total	Fold Enrichment	Bonferroni	Benjamini	FDR
GOTERM_BP_FAT	GO:0006576~biogenic amine metabolic process	13	2.34657	5.34E-04	549	97	13528	3.302424	0.852913	0.003322	0.976545
GOTERM_BP_FAT	GO:0046469~platelet activating factor metabolic process	4	0.722022	6.22E-04	549	5	13528	19.71293	0.892632	0.003814	1.135995
GOTERM_BP_FAT	GO:0006650~glycerophospholipid metabolic process	14	2.527076	9.72E-04	549	118	13528	2.923528	0.969475	0.00576	1.770569
GOTERM_BP_FAT	GO:0042439~ethanolamine and derivative metabolic process	7	1.263538	0.002196	549	34	13528	5.073181	0.999624	0.012207	3.956838
GOTERM_BP_FAT	GO:0006663~platelet activating factor biosynthetic process	3	0.541516	0.009307	549	4	13528	18.48087	1	0.046271	15.77949
GOTERM_BP_FAT	GO:0045017~glycerolipid biosynthetic process	6	1.083032	0.223355	549	80	13528	1.848087	1	0.646048	99.03631
GOTERM_BP_FAT	GO:0046474~glycerophospholipid biosynthetic process	5	0.902527	0.296641	549	68	13528	1.81185	1	0.757472	99.8439
GOTERM_BP_FAT	GO:0008654~phospholipid biosynthetic process	5	0.902527	0.59708	549	102	13528	1.2079	1	0.967452	99.99999
Annotation Cluster 165	Enrichment Score: 1.9073302820681572										
Category	Term	Count	%	PValue	List Total	Pop Hits	Pop Total	Fold Enrichment	Bonferroni	Benjamini	FDR
GOTERM_MF_FAT	GO:0004954~prostanoid receptor activity	4	0.722022	0.006276	523	10	12983	9.929637	0.992586	0.042844	9.180968
GOTERM_MF_FAT	GO:0004953~icosanoid receptor activity	4	0.722022	0.006276	523	10	12983	9.929637	0.992586	0.042844	9.180968
GOTERM_MF_FAT	GO:0004955~prostaglandin receptor activity	3	0.541516	0.048157	523	9	12983	8.274697	1	0.219677	52.9965
Annotation Cluster 166	Enrichment Score: 1.9062482357312076										
Category	Term	Count	%	PValue	List Total	Pop Hits	Pop Total	Fold Enrichment	Bonferroni	Benjamini	FDR
GOTERM_BP_FAT	GO:0001952~regulation of cell-matrix adhesion	7	1.263538	6.15E-04	549	27	13528	6.38845	0.88978	0.003782	1.122724
GOTERM_BP_FAT	GO:0010810~regulation of cell-substrate adhesion	8	1.444043	0.002335	549	46	13528	4.28542	0.999772	0.012936	4.202186
GOTERM_BP_FAT	GO:0010812~negative regulation of cell-substrate adhesion	4	0.722022	0.008551	549	11	13528	8.960424	1	0.04287	14.58998
GOTERM_BP_FAT	GO:0001954~positive regulation of cell-matrix adhesion	3	0.541516	0.048817	549	9	13528	8.213722	1	0.201909	60.11391
GOTERM_BP_FAT	GO:0001953~negative regulation of cell-matrix adhesion	3	0.541516	0.059419	549	10	13528	7.39235	1	0.238358	67.53433
GOTERM_BP_FAT	GO:0010811~positive regulation of cell-substrate adhesion	4	0.722022	0.102603	549	28	13528	3.520167	1	0.374717	86.30513

Annotation Cluster 167	Enrichment Score: 1.889590791276719										
Category	Term	Count	%	PValue	List Total	Pop Hits	Pop Total	Fold Enrichment	Bonferroni	Benjamini	FDR
GOTERM_BP_FAT	GO:0032965~regulation of collagen biosynthetic process	5	0.902527	7.00E-04	549	11	13528	11.20053	0.918877	0.00427	1.27777
GOTERM_BP_FAT	GO:0010712~regulation of collagen metabolic process	5	0.902527	0.001017	549	12	13528	10.26715	0.973961	0.005982	1.850479
GOTERM_BP_FAT	GO:0044246~regulation of multicellular organismal metabolic process	5	0.902527	0.00416	549	17	13528	7.247402	1	0.022102	7.369469
GOTERM_BP_FAT	GO:0032967~positive regulation of collagen biosynthetic process	3	0.541516	0.038996	549	8	13528	9.240437	1	0.16639	51.83347
GOTERM_BP_FAT	GO:0010714~positive regulation of collagen metabolic process	3	0.541516	0.038996	549	8	13528	9.240437	1	0.16639	51.83347
GOTERM_BP_FAT	GO:0044253~positive regulation of multicellular organismal metabolic process	3	0.541516	0.095146	549	13	13528	5.686423	1	0.353229	84.05715
GOTERM_BP_FAT	GO:0030168~platelet activation	4	0.722022	0.138405	549	32	13528	3.080146	1	0.469069	93.51603

Annotation Cluster 168	Enrichment Score: 1.8806030938957043										
Category	Term	Count	%	PValue	List Total	Pop Hits	Pop Total	Fold Enrichment	Bonferroni	Benjamini	FDR
GOTERM_BP_FAT	GO:0016202~regulation of striated muscle tissue development	9	1.624549	8.23E-04	549	50	13528	4.43541	0.94786	0.004927	1.500942
GOTERM_BP_FAT	GO:0048641~regulation of skeletal muscle tissue development	7	1.263538	9.21E-04	549	29	13528	5.947868	0.963268	0.005492	1.677434
GOTERM_BP_FAT	GO:0048634~regulation of muscle development	9	1.624549	9.42E-04	549	51	13528	4.348441	0.966006	0.005601	1.716427
GOTERM_BP_FAT	GO:0051153~regulation of striated muscle cell differentiation	7	1.263538	0.001585	549	32	13528	5.390255	0.996614	0.009045	2.870312
GOTERM_BP_FAT	GO:0048742~regulation of skeletal muscle fiber development	6	1.083032	0.002901	549	25	13528	5.91388	0.99997	0.015786	5.196233
GOTERM_BP_FAT	GO:0051147~regulation of muscle cell differentiation	7	1.263538	0.004491	549	39	13528	4.422773	1	0.023739	7.933583
GOTERM_MF_FAT	GO:0042826~histone deacetylase binding	6	1.083032	0.007365	523	31	12983	4.804663	0.996845	0.049261	10.69204
GOTERM_MF_FAT	GO:0004407~histone deacetylase activity	4	0.722022	0.020494	523	15	12983	6.619758	1	0.111077	27.14855
GOTERM_MF_FAT	GO:0033558~protein deacetylase activity	4	0.722022	0.020494	523	15	12983	6.619758	1	0.111077	27.14855
GOTERM_CC_FAT	GO:0016585~chromatin remodeling complex	8	1.444043	0.024748	520	71	12782	2.769664	0.999942	0.105997	29.39783
GOTERM_BP_FAT	GO:0016575~histone deacetylation	4	0.722022	0.029433	549	17	13528	5.797921	1	0.129764	42.22741
GOTERM_MF_FAT	GO:0019213~deacetylase activity	4	0.722022	0.033631	523	18	12983	5.516465	1	0.163771	40.74264
GOTERM_BP_FAT	GO:0006476~protein amino acid deacetylation	4	0.722022	0.05748	549	22	13528	4.480212	1	0.231858	66.28324
GOTERM_CC_FAT	GO:0000118~histone deacetylase complex	5	0.902527	0.083602	520	41	12782	2.997655	1	0.271959	70.26339
GOTERM_MF_FAT	GO:0003714~transcription corepressor activity	8	1.444043	0.365619	523	145	12983	1.369605	1	0.833131	99.90523
GOTERM_MF_FAT	GO:0016811~hydrolase activity, acting on carbon-nitrogen (but not peptide) bonds, in linear amides	4	0.722022	0.414381	523	58	12983	1.712006	1	0.870399	99.97212

Annotation Cluster 169	Enrichment Score: 1.8187250857676909

Category	Term	Count	%	PValue	List Total	Pop Hits	Pop Total	Fold Enrichment	Bonferroni	Benjamini	FDR
GOTERM_BP_FAT	GO:0007423~sensory organ development	22	3.971119	4.15E-04	549	229	13528	2.367274	0.774704	0.002639	0.760134
GOTERM_BP_FAT	GO:0048592~eye morphogenesis	8	1.444043	0.021274	549	69	13528	2.856947	1	0.097487	32.62637
GOTERM_BP_FAT	GO:0048593~camera-type eye morphogenesis	6	1.083032	0.031683	549	44	13528	3.360159	1	0.138114	44.63826
GOTERM_BP_FAT	GO:0001654~eye development	11	1.98556	0.041918	549	132	13528	2.05343	1	0.177107	54.45339
GOTERM_BP_FAT	GO:0043010~camera-type eye development	9	1.624549	0.068679	549	107	13528	2.072621	1	0.270273	72.9285

Annotation Cluster 170	Enrichment Score: 1.800465784720225										
Category	Term	Count	%	PValue	List Total	Pop Hits	Pop Total	Fold Enrichment	Bonferroni	Benjamini	FDR
GOTERM_BP_FAT	GO:0006690~icosanoid metabolic process	10	1.805054	9.45E-05	549	47	13528	5.242801	0.287571	6.66E-04	0.173452
GOTERM_BP_FAT	GO:0033559~unsaturated fatty acid metabolic process	10	1.805054	1.83E-04	549	51	13528	4.831601	0.480808	0.001234	0.335037
GOTERM_BP_FAT	GO:0046456~icosanoid biosynthetic process	7	1.263538	0.001332	549	31	13528	5.564134	0.991624	0.007709	2.418786
GOTERM_BP_FAT	GO:0006636~unsaturated fatty acid biosynthetic process	7	1.263538	0.002196	549	34	13528	5.073181	0.999624	0.012207	3.956838
GOTERM_BP_FAT	GO:0006631~fatty acid metabolic process	18	3.249097	0.002878	549	198	13528	2.240106	0.999968	0.015685	5.155765
GOTERM_BP_FAT	GO:0043450~alkene biosynthetic process	5	0.902527	0.006353	549	19	13528	6.484517	1	0.032637	11.04587
GOTERM_BP_FAT	GO:0019370~leukotriene biosynthetic process	5	0.902527	0.006353	549	19	13528	6.484517	1	0.032637	11.04587
GOTERM_BP_FAT	GO:0006691~leukotriene metabolic process	5	0.902527	0.010894	549	22	13528	5.600265	1	0.053469	18.2221
GOTERM_BP_FAT	GO:0043449~cellular alkene metabolic process	5	0.902527	0.012774	549	23	13528	5.356775	1	0.061543	21.02986
GOTERM_BP_FAT	GO:0000038~very-long-chain fatty acid metabolic process	4	0.722022	0.039521	549	19	13528	5.187614	1	0.16808	52.31419
GOTERM_BP_FAT	GO:0006633~fatty acid biosynthetic process	8	1.444043	0.040658	549	79	13528	2.495308	1	0.172365	53.34012
GOTERM_BP_FAT	GO:0016053~organic acid biosynthetic process	10	1.805054	0.177653	549	155	13528	1.589753	1	0.557298	97.24589
GOTERM_BP_FAT	GO:0046394~carboxylic acid biosynthetic process	10	1.805054	0.177653	549	155	13528	1.589753	1	0.557298	97.24589
GOTERM_BP_FAT	GO:0008610~lipid biosynthetic process	13	2.34657	0.662184	549	323	13528	0.99175	1	0.982444	100
GOTERM_MF_FAT	GO:0016702~oxidoreductase activity, acting on single donors with incorporation of molecular oxygen, incorporation of two atoms of oxygen	3	0.541516	0.74989	523	66	12983	1.128368	1	0.991578	100
GOTERM_MF_FAT	GO:0016701~oxidoreductase activity, acting on single donors with incorporation of molecular oxygen	3	0.541516	0.757309	523	67	12983	1.111526	1	0.992076	100

Annotation Cluster 171	Enrichment Score: 1.7938591991685429										
Category	Term	Count	%	PValue	List Total	Pop Hits	Pop Total	Fold Enrichment	Bonferroni	Benjamini	FDR
GOTERM_MF_FAT	GO:0005138~interleukin-6 receptor binding	4	0.722022	0.002004	523	7	12983	14.1852	0.790371	0.016484	3.021368

GOTERM_BP_FAT	GO:0042516~regulation of tyrosine phosphorylation of Stat3 protein	5	0.902527	0.00416	549	17	13528	7.247402	1	0.022102	7.369469
GOTERM_BP_FAT	GO:0030278~regulation of ossification	10	1.805054	0.00417	549	78	13528	3.159124	1	0.022121	7.386366
GOTERM_CC_FAT	GO:0005896~interleukin-6 receptor complex	3	0.541516	0.004804	520	3	12782	24.58077	0.846363	0.026038	6.470377
GOTERM_BP_FAT	GO:0042517~positive regulation of tyrosine phosphorylation of Stat3 protein	4	0.722022	0.008551	549	11	13528	8.960424	1	0.04287	14.58998
GOTERM_BP_FAT	GO:0045669~positive regulation of osteoblast differentiation	4	0.722022	0.078613	549	25	13528	3.942587	1	0.302568	77.76794
GOTERM_BP_FAT	GO:0045667~regulation of osteoblast differentiation	5	0.902527	0.095172	549	43	13528	2.865252	1	0.352968	84.06558
GOTERM_BP_FAT	GO:0031016~pancreas development	3	0.541516	0.417425	549	35	13528	2.1121	1	0.878346	99.99509

Annotation Cluster 172 Enrichment Score: 1.7368096797101147

Category	Term	Count	%	PValue	List Total	Pop Hits	Pop Total	Fold Enrichment	Bonferroni	Benjamini	FDR
GOTERM_BP_FAT	GO:0007266~Rho protein signal transduction	8	1.444043	7.28E-04	549	38	13528	5.187614	0.926612	0.004417	1.328409
GOTERM_BP_FAT	GO:0030810~positive regulation of nucleotide biosynthetic process	4	0.722022	0.020907	549	15	13528	6.570978	1	0.09599	32.16016
GOTERM_BP_FAT	GO:0045981~positive regulation of nucleotide metabolic process	4	0.722022	0.020907	549	15	13528	6.570978	1	0.09599	32.16016
GOTERM_BP_FAT	GO:0030801~positive regulation of cyclic nucleotide metabolic process	4	0.722022	0.020907	549	15	13528	6.570978	1	0.09599	32.16016
GOTERM_BP_FAT	GO:0030804~positive regulation of cyclic nucleotide biosynthetic process	4	0.722022	0.020907	549	15	13528	6.570978	1	0.09599	32.16016
GOTERM_BP_FAT	GO:0030816~positive regulation of cAMP metabolic process	3	0.541516	0.070723	549	11	13528	6.720318	1	0.277048	73.99903
GOTERM_BP_FAT	GO:0030819~positive regulation of cAMP biosynthetic process	3	0.541516	0.070723	549	11	13528	6.720318	1	0.277048	73.99903

Annotation Cluster 173 Enrichment Score: 1.663856389111618

Category	Term	Count	%	PValue	List Total	Pop Hits	Pop Total	Fold Enrichment	Bonferroni	Benjamini	FDR
GOTERM_BP_FAT	GO:0032388~positive regulation of intracellular transport	7	1.263538	0.001112	549	30	13528	5.749605	0.981519	0.006511	2.022618
GOTERM_BP_FAT	GO:0033158~regulation of protein import into nucleus, translocation	4	0.722022	0.004625	549	9	13528	10.95163	1	0.024368	8.160688
GOTERM_BP_FAT	GO:0042306~regulation of protein import into nucleus	7	1.263538	0.008197	549	44	13528	3.920185	1	0.041359	14.02934
GOTERM_BP_FAT	GO:0017015~regulation of transforming growth factor beta receptor signaling pathway	6	1.083032	0.019782	549	39	13528	3.790949	1	0.091369	30.71515
GOTERM_BP_FAT	GO:0033160~positive regulation of protein import into nucleus, translocation	3	0.541516	0.030042	549	7	13528	10.5605	1	0.13214	42.88908
GOTERM_BP_FAT	GO:0010718~positive regulation of epithelial to mesenchymal transition	3	0.541516	0.030042	549	7	13528	10.5605	1	0.13214	42.88908
GOTERM_BP_FAT	GO:0010770~positive regulation of cell morphogenesis involved in differentiation	3	0.541516	0.030042	549	7	13528	10.5605	1	0.13214	42.88908
GOTERM_BP_FAT	GO:0007183~SMAD protein complex assembly	3	0.541516	0.038996	549	8	13528	9.240437	1	0.16639	51.83347
GOTERM_BP_FAT	GO:0010717~regulation of epithelial to mesenchymal transition	3	0.541516	0.048817	549	9	13528	8.213722	1	0.201909	60.11391

GOTERM_BP_FAT	GO:0046824~positive regulation of nucleocytoplasmic transport	4	0.722022	0.05748	549	22	13528	4.480212	1	0.231858	66.28324
GOTERM_BP_FAT	GO:0010769~regulation of cell morphogenesis involved in differentiation	6	1.083032	0.201443	549	77	13528	1.920091	1	0.605288	98.39364

Annotation Cluster 174 Enrichment Score: 1.6601218767459671

Category	Term	Count	%	PValue	List Total	Pop Hits	Pop Total	Fold Enrichment	Bonferroni	Benjamini	FDR
GOTERM_BP_FAT	GO:0051146~striated muscle cell differentiation	16	2.888087	2.21E-06	549	88	13528	4.480212	0.007886	1.98E-05	0.004053
GOTERM_BP_FAT	GO:0007519~skeletal muscle tissue development	9	1.624549	0.005007	549	66	13528	3.360159	1	0.026244	8.805976
GOTERM_BP_FAT	GO:0060538~skeletal muscle organ development	9	1.624549	0.005007	549	66	13528	3.360159	1	0.026244	8.805976
GOTERM_BP_FAT	GO:0060056~mammary gland involution	3	0.541516	0.015098	549	5	13528	14.7847	1	0.071837	24.37517
GOTERM_BP_FAT	GO:0060443~mammary gland morphogenesis	3	0.541516	0.022043	549	6	13528	12.32058	1	0.100612	33.59233
GOTERM_CC_FAT	GO:0031594~neuromuscular junction	4	0.722022	0.057801	520	22	12782	4.469231	1	0.203132	56.26778
GOTERM_BP_FAT	GO:0055002~striated muscle cell development	6	1.083032	0.058492	549	52	13528	2.843211	1	0.235271	66.94178
GOTERM_BP_FAT	GO:0055001~muscle cell development	6	1.083032	0.075587	549	56	13528	2.640125	1	0.293034	76.38828
GOTERM_BP_FAT	GO:0050808~synapse organization	6	1.083032	0.100334	549	61	13528	2.423721	1	0.368532	85.65494
GOTERM_BP_FAT	GO:0007528~neuromuscular junction development	3	0.541516	0.193313	549	20	13528	3.696175	1	0.588836	98.0652
GOTERM_BP_FAT	GO:0048741~skeletal muscle fiber development	3	0.541516	0.3145	549	28	13528	2.640125	1	0.779463	99.90266
GOTERM_BP_FAT	GO:0048747~muscle fiber development	3	0.541516	0.417425	549	35	13528	2.1121	1	0.878346	99.99509

Annotation Cluster 175 Enrichment Score: 1.6239331410424787

Category	Term	Count	%	PValue	List Total	Pop Hits	Pop Total	Fold Enrichment	Bonferroni	Benjamini	FDR
GOTERM_BP_FAT	GO:0035239~tube morphogenesis	13	2.34657	0.005401	549	127	13528	2.522324	1	0.028081	9.466987
GOTERM_BP_FAT	GO:0060429~epithelium development	18	3.249097	0.011022	549	227	13528	1.953925	1	0.05401	18.4162
GOTERM_BP_FAT	GO:0002009~morphogenesis of an epithelium	7	1.263538	0.225676	549	101	13528	1.707804	1	0.649114	99.08786

Annotation Cluster 176 Enrichment Score: 1.6180316503983794

Category	Term	Count	%	PValue	List Total	Pop Hits	Pop Total	Fold Enrichment	Bonferroni	Benjamini	FDR
GOTERM_BP_FAT	GO:0010553~negative regulation of specific transcription from RNA polymerase II promoter	14	2.527076	4.26E-09	549	41	13528	8.414057	1.53E-05	5.44E-08	7.83E-06
GOTERM_CC_FAT	GO:0016585~chromatin remodeling complex	8	1.444043	0.024748	520	71	12782	2.769664	0.999942	0.105997	29.39783
GOTERM_BP_FAT	GO:0018205~peptidyl-lysine modification	4	0.722022	0.029433	549	17	13528	5.797921	1	0.129764	42.22741
GOTERM_CC_FAT	GO:0000118~histone deacetylase complex	5	0.902527	0.083602	520	41	12782	2.997655	1	0.271959	70.26339
GOTERM_BP_FAT	GO:0016569~covalent chromatin modification	9	1.624549	0.13918	549	126	13528	1.760083	1	0.470697	93.62235

Category	Term	Count	%	PValue	List Total	Pop Hits	Pop Total	Fold Enrichment	Bonferroni	Benjamini	FDR
GOTERM_BP_FAT	GO:0016570~histone modification	8	1.444043	0.22545	549	122	13528	1.615814	1	0.649114	99.08295
GOTERM_BP_FAT	GO:0016568~chromatin modification	13	2.34657	0.42772	549	274	13528	1.169106	1	0.885978	99.99646
GOTERM_BP_FAT	GO:0006325~chromatin organization	13	2.34657	0.844169	549	378	13528	0.847447	1	0.998721	100
GOTERM_BP_FAT	GO:0051276~chromosome organization	15	2.707581	0.931399	549	485	13528	0.762098	1	0.999926	100
Annotation Cluster 177	Enrichment Score: 1.6078921526912267										
Category	Term	Count	%	PValue	List Total	Pop Hits	Pop Total	Fold Enrichment	Bonferroni	Benjamini	FDR
GOTERM_BP_FAT	GO:0051283~negative regulation of sequestering of calcium ion	4	0.722022	0.017234	549	14	13528	7.040333	1	0.080606	27.33166
GOTERM_BP_FAT	GO:0051282~regulation of sequestering of calcium ion	4	0.722022	0.017234	549	14	13528	7.040333	1	0.080606	27.33166
GOTERM_BP_FAT	GO:0051209~release of sequestered calcium ion into cytosol	4	0.722022	0.017234	549	14	13528	7.040333	1	0.080606	27.33166
GOTERM_BP_FAT	GO:0060402~calcium ion transport into cytosol	4	0.722022	0.039521	549	19	13528	5.187614	1	0.16808	52.31419
GOTERM_BP_FAT	GO:0060401~cytosolic calcium ion transport	4	0.722022	0.045138	549	20	13528	4.928233	1	0.188759	57.18375
Annotation Cluster 178	Enrichment Score: 1.6027198374245903										
Category	Term	Count	%	PValue	List Total	Pop Hits	Pop Total	Fold Enrichment	Bonferroni	Benjamini	FDR
GOTERM_BP_FAT	GO:0045744~negative regulation of G-protein coupled receptor protein signaling pathway	4	0.722022	0.017234	549	14	13528	7.040333	1	0.080606	27.33166
GOTERM_BP_FAT	GO:0002029~desensitization of G-protein coupled receptor protein signaling pathway	3	0.541516	0.030042	549	7	13528	10.5605	1	0.13214	42.88908
GOTERM_BP_FAT	GO:0022401~adaptation of signaling pathway	3	0.541516	0.030042	549	7	13528	10.5605	1	0.13214	42.88908
Annotation Cluster 179	Enrichment Score: 1.5889697127737574										
Category	Term	Count	%	PValue	List Total	Pop Hits	Pop Total	Fold Enrichment	Bonferroni	Benjamini	FDR
GOTERM_BP_FAT	GO:0035303~regulation of dephosphorylation	6	1.083032	0.003474	549	26	13528	5.686423	0.999996	0.018682	6.191788
GOTERM_BP_FAT	GO:0010922~positive regulation of phosphatase activity	3	0.541516	0.030042	549	7	13528	10.5605	1	0.13214	42.88908
GOTERM_BP_FAT	GO:0010921~regulation of phosphatase activity	3	0.541516	0.163867	549	18	13528	4.106861	1	0.525958	96.26244
Annotation Cluster 180	Enrichment Score: 1.5485081920574304										
Category	Term	Count	%	PValue	List Total	Pop Hits	Pop Total	Fold Enrichment	Bonferroni	Benjamini	FDR
GOTERM_BP_FAT	GO:0019827~stem cell maintenance	5	0.902527	0.017124	549	25	13528	4.928233	1	0.08042	27.18208
GOTERM_BP_FAT	GO:0048864~stem cell development	5	0.902527	0.019606	549	26	13528	4.738686	1	0.0907	30.48577
GOTERM_BP_FAT	GO:0048863~stem cell differentiation	5	0.902527	0.039031	549	32	13528	3.850182	1	0.166333	51.865
GOTERM_BP_FAT	GO:0035019~somatic stem cell maintenance	3	0.541516	0.048817	549	9	13528	8.213722	1	0.201909	60.11391
Annotation Cluster 181	Enrichment Score: 1.5341879191475807										

Category	Term	Count	%	PValue	List Total	Pop Hits	Pop Total	Fold Enrichment	Bonferroni	Benjamini	FDR
GOTERM_BP_FAT	GO:0006816~calcium ion transport	17	3.068592	2.04E-04	549	142	13528	2.949999	0.51856	0.001368	0.373551
GOTERM_BP_FAT	GO:0015674~di-, tri-valent inorganic cation transport	19	3.429603	2.85E-04	549	176	13528	2.660126	0.640109	0.001863	0.521862
GOTERM_BP_FAT	GO:0030001~metal ion transport	20	3.610108	0.517268	549	465	13528	1.059835	1	0.938275	99.99984
GOTERM_BP_FAT	GO:0006812~cation transport	21	3.790614	0.732491	549	553	13528	0.93574	1	0.992258	100
GOTERM_BP_FAT	GO:0006811~ion transport	23	4.151625	0.970014	549	768	13528	0.737952	1	0.999996	100
Annotation Cluster 182	Enrichment Score: 1.5281913222384917										
Category	Term	Count	%	PValue	List Total	Pop Hits	Pop Total	Fold Enrichment	Bonferroni	Benjamini	FDR
GOTERM_BP_FAT	GO:0050803~regulation of synapse structure and activity	5	0.902527	0.014848	549	24	13528	5.133576	1	0.070778	24.02281
GOTERM_BP_FAT	GO:0051963~regulation of synaptogenesis	4	0.722022	0.034284	549	18	13528	5.475815	1	0.148221	47.30622
GOTERM_BP_FAT	GO:0050807~regulation of synapse organization	4	0.722022	0.051127	549	21	13528	4.693555	1	0.21014	61.85627
Annotation Cluster 183	Enrichment Score: 1.4786815931720299										
Category	Term	Count	%	PValue	List Total	Pop Hits	Pop Total	Fold Enrichment	Bonferroni	Benjamini	FDR
GOTERM_BP_FAT	GO:0051495~positive regulation of cytoskeleton organization	9	1.624549	3.95E-04	549	45	13528	4.928233	0.75731	0.002521	0.722338
GOTERM_BP_FAT	GO:0051493~regulation of cytoskeleton organization	14	2.527076	0.003498	549	136	13528	2.536591	0.999997	0.018778	6.23221
GOTERM_BP_FAT	GO:0032956~regulation of actin cytoskeleton organization	10	1.805054	0.009842	549	89	13528	2.76867	1	0.048608	16.61018
GOTERM_BP_FAT	GO:0032970~regulation of actin filament-based process	10	1.805054	0.0121	549	92	13528	2.678388	1	0.058768	20.03458
GOTERM_BP_FAT	GO:0031334~positive regulation of protein complex assembly	6	1.083032	0.012723	549	35	13528	4.2242	1	0.061386	20.955
GOTERM_BP_FAT	GO:0043254~regulation of protein complex assembly	9	1.624549	0.029278	549	90	13528	2.464117	1	0.129273	42.05758
GOTERM_BP_FAT	GO:0032273~positive regulation of protein polymerization	4	0.722022	0.078613	549	25	13528	3.942587	1	0.302568	77.76794
GOTERM_BP_FAT	GO:0032271~regulation of protein polymerization	6	1.083032	0.140839	549	68	13528	2.174221	1	0.474614	93.84434
GOTERM_BP_FAT	GO:0030833~regulation of actin filament polymerization	4	0.722022	0.375019	549	54	13528	1.825272	1	0.842239	99.98217
GOTERM_BP_FAT	GO:0008064~regulation of actin polymerization or depolymerization	4	0.722022	0.451494	549	61	13528	1.615814	1	0.901857	99.99838
GOTERM_BP_FAT	GO:0030832~regulation of actin filament length	4	0.722022	0.472667	549	63	13528	1.564518	1	0.915036	99.99921
Annotation Cluster 184	Enrichment Score: 1.4616399619972504										
Category	Term	Count	%	PValue	List Total	Pop Hits	Pop Total	Fold Enrichment	Bonferroni	Benjamini	FDR
GOTERM_BP_FAT	GO:0030218~erythrocyte differentiation	6	1.083032	0.029014	549	43	13528	3.438302	1	0.128326	41.76791
GOTERM_BP_FAT	GO:0043353~enucleate erythrocyte differentiation	3	0.541516	0.030042	549	7	13528	10.5605	1	0.13214	42.88908

GOTERM_BP_FAT	GO:0034101~erythrocyte homeostasis	6	1.083032	0.047287	549	49	13528	3.017286	1	0.19655	58.91916
Annotation Cluster 185	Enrichment Score: 1.4416595162202075										
Category	Term	Count	%	PValue	List Total	Pop Hits	Pop Total	Fold Enrichment	Bonferroni	Benjamini	FDR
GOTERM_BP_FAT	GO:0050905~neuromuscular process	8	1.444043	0.01233	549	62	13528	3.179505	1	0.059776	20.37534
GOTERM_BP_FAT	GO:0035235~ionotropic glutamate receptor signaling pathway	3	0.541516	0.048817	549	9	13528	8.213722	1	0.201909	60.11391
GOTERM_BP_FAT	GO:0007215~glutamate signaling pathway	4	0.722022	0.078613	549	25	13528	3.942587	1	0.302568	77.76794
Annotation Cluster 186	Enrichment Score: 1.4289033543599772										
Category	Term	Count	%	PValue	List Total	Pop Hits	Pop Total	Fold Enrichment	Bonferroni	Benjamini	FDR
GOTERM_BP_FAT	GO:0002274~myeloid leukocyte activation	9	1.624549	4.61E-04	549	46	13528	4.821098	0.80877	0.002898	0.843395
GOTERM_BP_FAT	GO:0002532~production of molecular mediator of acute inflammatory response	3	0.541516	0.030042	549	7	13528	10.5605	1	0.13214	42.88908
GOTERM_BP_FAT	GO:0002275~myeloid cell activation during immune response	3	0.541516	0.163867	549	18	13528	4.106861	1	0.525958	96.26244
GOTERM_BP_FAT	GO:0002366~leukocyte activation during immune response	4	0.722022	0.177723	549	36	13528	2.737907	1	0.557036	97.2502
GOTERM_BP_FAT	GO:0002263~cell activation during immune response	4	0.722022	0.177723	549	36	13528	2.737907	1	0.557036	97.2502
Annotation Cluster 187	Enrichment Score: 1.4165140393402262										
Category	Term	Count	%	PValue	List Total	Pop Hits	Pop Total	Fold Enrichment	Bonferroni	Benjamini	FDR
GOTERM_MF_FAT	GO:0031406~carboxylic acid binding	13	2.34657	0.01332	523	144	12983	2.241064	0.999971	0.079564	18.5453
GOTERM_MF_FAT	GO:0005504~fatty acid binding	5	0.902527	0.064981	523	38	12983	3.266328	1	0.281986	64.21873
GOTERM_MF_FAT	GO:0033293~monocarboxylic acid binding	6	1.083032	0.065036	523	54	12983	2.758232	1	0.280694	64.25056
Annotation Cluster 188	Enrichment Score: 1.3715598438839147										
Category	Term	Count	%	PValue	List Total	Pop Hits	Pop Total	Fold Enrichment	Bonferroni	Benjamini	FDR
GOTERM_BP_FAT	GO:0022415~viral reproductive process	8	1.444043	0.01233	549	62	13528	3.179505	1	0.059776	20.37534
GOTERM_BP_FAT	GO:0016032~viral reproduction	8	1.444043	0.024491	549	71	13528	2.776469	1	0.110714	36.57859
GOTERM_BP_FAT	GO:0019079~viral genome replication	4	0.722022	0.024973	549	16	13528	6.160291	1	0.112376	37.15234
GOTERM_BP_FAT	GO:0019058~viral infectious cycle	5	0.902527	0.095172	549	43	13528	2.865252	1	0.352968	84.06558
GOTERM_BP_FAT	GO:0019048~virus-host interaction	3	0.541516	0.193313	549	20	13528	3.696175	1	0.588836	98.0652
Annotation Cluster 189	Enrichment Score: 1.3156689431835942										
Category	Term	Count	%	PValue	List Total	Pop Hits	Pop Total	Fold Enrichment	Bonferroni	Benjamini	FDR
GOTERM_MF_FAT	GO:0030374~ligand-dependent nuclear receptor transcription	6	1.083032	0.013871	523	36	12983	4.137349	0.999981	0.081496	19.2379

Category	Term	Count	%	PValue	List Total	Pop Hits	Pop Total	Fold Enrichment	Bonferroni	Benjamini	FDR
	coactivator activity										
GOTERM_BP_FAT	GO:0006352~transcription initiation	8	1.444043	0.05086	549	83	13528	2.375052	1	0.209371	61.6581
GOTERM_BP_FAT	GO:0006367~transcription initiation from RNA polymerase II promoter	7	1.263538	0.056851	549	68	13528	2.536591	1	0.230071	65.86745
GOTERM_MF_FAT	GO:0046966~thyroid hormone receptor binding	4	0.722022	0.136178	523	32	12983	3.103011	1	0.486692	89.34573

Annotation Cluster 190	Enrichment Score: 1.311431178116753										
Category	Term	Count	%	PValue	List Total	Pop Hits	Pop Total	Fold Enrichment	Bonferroni	Benjamini	FDR
GOTERM_BP_FAT	GO:0052200~response to host defenses	3	0.541516	0.048817	549	9	13528	8.213722	1	0.201909	60.11391
GOTERM_BP_FAT	GO:0052173~response to defenses of other organism during symbiotic interaction	3	0.541516	0.048817	549	9	13528	8.213722	1	0.201909	60.11391
GOTERM_BP_FAT	GO:0075136~response to host	3	0.541516	0.048817	549	9	13528	8.213722	1	0.201909	60.11391

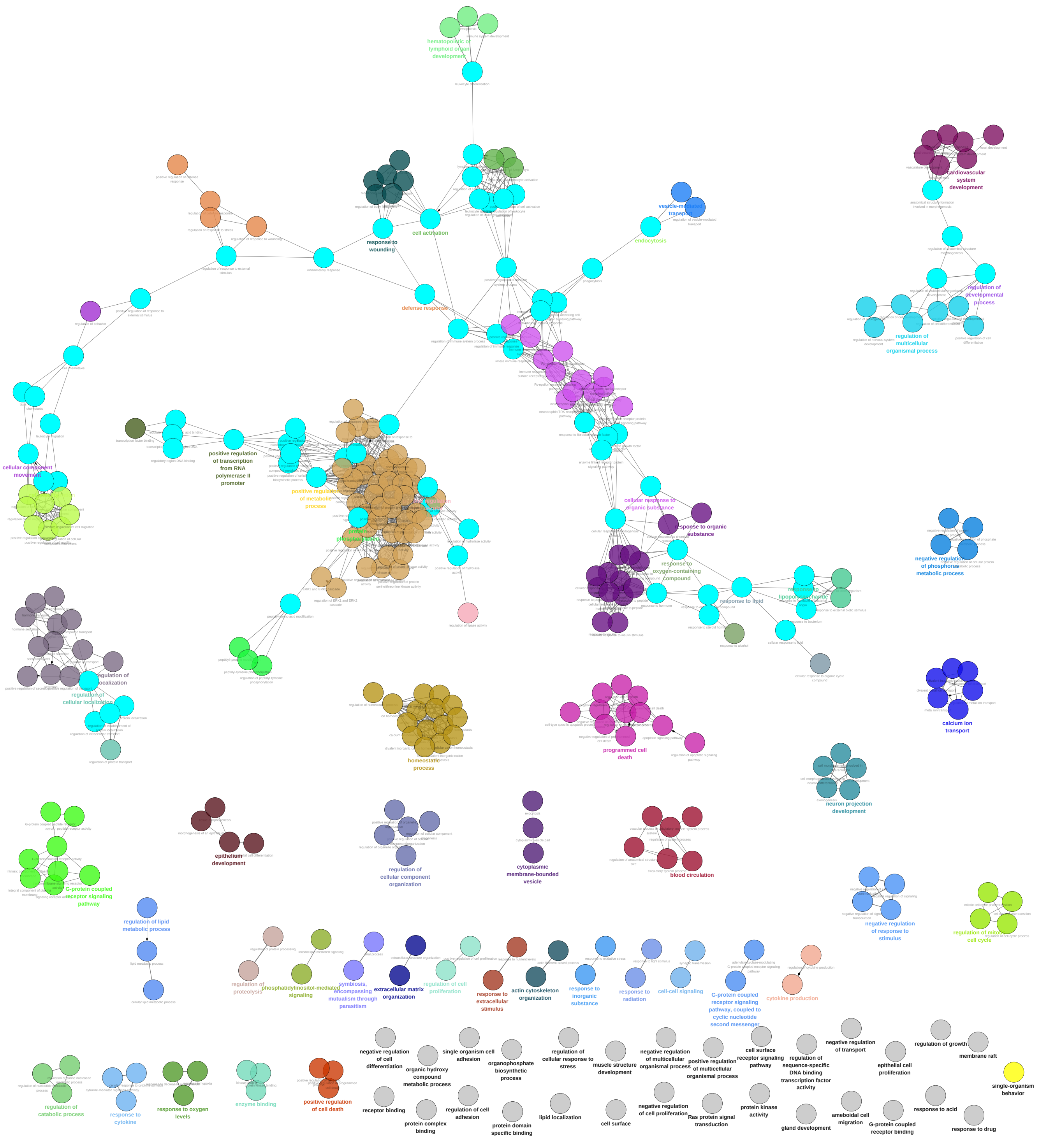

Figure S1. Functional GO groups enriched by genes of stress-related disease/system common module.
